# Mapping Evidence on Integrated 24-Hour Movement Behaviors in Children and Adolescents: A Scoping Review of Reviews

**DOI:** 10.3390/children12030260

**Published:** 2025-02-20

**Authors:** Andressa Ferreira da Silva, Priscila Custódio Martins, Leandro Narciso Santiago, Diego Augusto Santos Silva

**Affiliations:** 1 Núcleo de Pesquisa em Cineantropometria e Desempenho Humano, Departamento de Educação Física, Centro de Desportos, Universidade Federal de Santa Catarina, Florianópolis 88040-900, SC, Brazilleandro.santiago@posgrad.ufsc.br (L.N.S.); 2 Grupo de Estudo e Pesquisa em Promoção da Saúde, Universidade do Extremo Sul Catarinense, Criciúma 88806-000, SC, Brazil

**Keywords:** motor activity, healthy lifestyle, sleep, sedentary behavior, exercise, adolescent behavior, child behavior

## Abstract

Background: There has been a substantial increase in research on the new 24-hour movement paradigm, emphasizing the importance of considering the “whole day” and investigating integrated movement behaviors (physical activity, sedentary behavior, and sleep). This scoping review aims to map the evidence from reviews that have summarized information on integrated 24-hour movement behaviors in children and adolescents. Methods: Eight databases were searched in May 2023, with an update in October 2024. The review followed the PRISMA-ScR framework and the guidelines of the Joanna Briggs Institute Reviewer’s Manual. Results: National 24-hour movement guidelines for children and adolescents exist in only a few countries (Australia, Canada, New Zealand, and South Africa). There is a lack of valid and reliable measurement tools for assessing 24-hour movement. Globally, children and adolescents, with and without disabilities, show low adherence to these guidelines. Reallocating time to moderate-to-vigorous physical activity was beneficial, while other reallocations had mixed results to health. COVID-19 reduced physical activity and increased screen time and sleep. Healthy movement behaviors are positively associated with better health outcomes in children and adolescents. There is a possible relationship between adherence to 24-hour movement behaviors and cognitive function, pollution measures, and eHealth interventions. Inconsistencies were identified in the terms used. Conclusions: High-quality research is needed to develop measurement tools that assess the long-term health impact of 24-hour movement and to create solutions for improving adherence, mainly in countries lacking specific guidelines.

## 1. Introduction

The 21st-century technological revolution has led to an increase in time spent engaging in sedentary behaviors, which has, in turn, led to a decrease in physical activity and adequate sleep [1]. Traditionally, these modifiable lifestyle factors—physical activity, sedentary behavior, and sleep—were investigated in isolation [2]. Consequently, the body of evidence has consolidated the importance of meeting the recommendations for each of these behaviors independently in relation to health and quality of life across different populations [2,3,4,5,6]. However, a holistic perspective is now considered more appropriate for establishing relationships between the daily composition of movement behaviors and health indicators [7], particularly regarding their impact on physical and mental health outcomes [8]. The three movement behaviors—physical activity, sedentary behavior, and sleep—comprise an interdependent and collinear 24-hour daily cycle [2]. In this context, adopting a holistic approach is considered appropriate for elucidating the relationships between 24-hour movement behaviors and health indicators, which are crucial for disease prevention and health promotion throughout a person’s lifespan [7]. Consequently, a new perspective on movement behaviors emerged in 2016, when the Canadian Society for Exercise Physiology launched the “Canadian 24-Hour Movement Guidelines for Children and Adolescents (ages 5 to 17): An Integration of Physical Activity, Sedentary Behavior, and Sleep” [2]. Since then, the scientific literature in this area has been expanding.

Different terms are found in the literature to describe adherence to the 24-hour movement guidelines, mainly due to the novelty of the field and the lack of standardized terminology. While each term has its advantages, the absence of a formal agreement on terminology may hinder the synthesis of new knowledge regarding the combined impact of these three movement behaviors on health [9]. The term “24-hour movement guidelines” is widely used and refers to compliance with each of the movement behavior recommendations established by different national guidelines [2,10,11]. Similarly, the terms “24-hour movement behavior” and “integration of physical activity, sedentary behavior, and sleep” are commonly employed to denote the combination/integration of the three movement behaviors (physical activity, sedentary behavior, and sleep) within the 24-hour daily cycle of children and adolescents. However, these terms may also refer to individual or dual combinations of movement behaviors [2,12]. Additionally, the term “movement behavior” is sometimes used in the literature to refer to the three daily movement behaviors collectively or to adherence to their recommendations, but it is often analyzed in isolation [2]. Given these variations and the existence of other related terms, this scoping review standardizes the use of “24-hour movement”, explicitly focusing on studies that assess physical activity, sedentary behavior, and sleep as an integrated whole within a continuous 24-hour period.

To promote health benefits, the Canadian 24-hour movement guidelines for children and adolescents recommend accumulating at least 60 min per day of moderate-to-vigorous physical activity (including aerobic activities, vigorous physical activities, and muscle- and bone-strengthening activities at least three times per week), engaging in various structured and unstructured light physical activities, reducing prolonged sedentary time—especially screen time (limiting recreational screen use to no more than two hours per day)—and maintaining consistent sleep and wake times (9–11 h of uninterrupted sleep per night for children aged 5–13 years and 8–10 h per night for adolescents aged 14–17 years) [2]. The guidelines also suggest that small modifications to daily movement behaviors can yield significant health benefits, such as maintaining sufficient sleep quantity and quality, spending more time outdoors, and replacing sedentary behaviors and light physical activity with moderate-to-vigorous physical activity [2]. Furthermore, any increase in time allocated to one behavior must be compensated by a reduction in time spent on other behaviors [13], which can result in meeting or failing to meet one recommendation at the expense of another. In this context, several factors can influence the composition of 24-hour movement behaviors, such as technological advances and access to technology, social characteristics [1,14], environmental factors [7,14,15], and cultural diversity [14]. However, adolescents who adopt structured routines and engage in daily health-promoting behaviors tend to report more favorable health trajectories [16].

Systematic reviews on 24-hour movement behaviors have consistently demonstrated that children and adolescents who do not meet the Canadian 24-hour movement guidelines tend to exhibit less favorable health indicators compared to those who do [7,17]. In children, adherence to these guidelines is positively associated with better cognitive function, improved health-related quality of life, and healthier eating habits [7], while in both children and adolescents, adherence is associated with lower body adiposity and obesity [3,7], higher aerobic fitness [7], favorable cardiometabolic health [3], and positive indicators for mental, social, and emotional health [18]. While high levels of physical activity and low levels of sedentary behavior are considered the optimal combination for physical, psychological, and educational outcomes, ensuring adequate sleep duration further enhances these benefits [17]. Despite the described benefits, systematic reviews have reported a low global prevalence of children and adolescents adhering to the 24-hour movement guidelines [1,7]. These studies indicate that only 5% to 11% of children and 2% to 10% of adolescents meet the guidelines [1,7].

Since 2016, an increasing number of countries have adopted the guidelines, leading to a substantial expansion of research on 24-hour movement behaviors, particularly between 2023 and 2024. This has established the field as a critical area of public health research [14,19]. Although significant progress has been made in understanding the importance of concurrently meeting the three movement behavior recommendations, as well as in identifying prevalence rates and associations with specific health outcomes such as obesity and mental health [20,21], the field has yet to reach full consolidation. Several health outcomes, including associations with chronic diseases [13,22], remain underexplored or insufficiently addressed [22]. Additionally, advancements in measurement precision—such as the application of compositional analyses [14]—are still in their early stages, as are investigations into the impact of climate change, academic performance, and variations in time allocation across movement behaviors [22,23,24,25]. Furthermore, research integrating exercise physiology, motor development, and cognitive development [13] remains scarce, particularly in studies examining the combined effects of these three movement behaviors.

Previous reviews have primarily examined whether children and adolescents meet 24-hour movement behavior recommendations and have explored the associations between adherence—either individually or simultaneously—and specific health outcomes [2,3,4,5,6]. However, no prior review has systematically assessed the global progress of 24-hour movement behavior research, particularly in terms of advancements in simultaneous adherence to all three movement behaviors. Therefore, systematically mapping this emerging research area is essential for identifying progress, highlighting existing gaps, and guiding future investigations.

Scoping reviews provide a rigorous and transparent methodological framework for mapping research areas and clarifying the field in terms of research volume, nature, and characteristics [26]. This type of review is instrumental in identifying evidence gaps, summarizing findings concisely, and facilitating the effective application of research outcomes in decision-making processes [26,27]. Given the rapidly growing body of original studies and reviews that, within a short period, have attracted significant interest from both public health research and clinical practice [28], there remains a lack of integration of these findings. This scoping review of reviews aims to address this gap by providing the first comprehensive mapping of the scientific evidence summarizing integrated 24-hour movement behaviors in children and adolescents. Users of these synthesized findings depend on clear and transparent knowledge translation to interpret and apply research outcomes effectively, driving evidence-based improvements within and beyond healthcare settings [29].

## 2. Materials and Methods

### 2.1. Protocol and Registration

This scoping review of reviews was registered on the Open Science Framework (OSF) at https://osf.io/acwj8/ (accessed on 2 May 2023) and adhered to the procedures outlined in the Preferred Reporting Items for Systematic Reviews and Meta-Analyses Extension for Scoping Reviews (PRISMA-ScR) [30] and the recommendations from the Joanna Briggs Institute Reviewer’s Manual [27]. The term “24-hour movement” was selected to refer to the integration of time spent on the three movement behaviors over a 24-hour period.

### 2.2. Search Strategy

A systematic search was conducted in May 2023 and updated in October 2024, across seven databases: MEDLINE (via PubMed), Web of Science, Scopus (via Elsevier), SPORTDiscus (via EBSCOhost), LILACS (via Virtual Health Library), PsycINFO (via American Psychological Association—APA), and CINAHL (via EBSCOhost), and SciELO. The selection of databases was strategically planned to ensure the comprehensiveness and representativeness of the available scientific literature on the topic, considering those commonly used in other reviews on the same subject [3,4,5,6,8,31]. The combination of these databases allowed for a comprehensive and balanced search, including high-quality studies in health, sports sciences, psychology, human behavior, and regional research, which are essential for mapping the evidence on 24-hour movement behaviors in children and adolescents globally. An advanced search tool was employed, utilizing a combination of keywords in English, Portuguese, and Spanish derived from Health Sciences Descriptors (DeCS), Medical Subject Headings (MeSH), and keywords from scientific sources on the topic. Manual searches were also conducted based on the references of the included studies (Table S1).

### 2.3. Eligibility Criteria

The inclusion criteria were as follows: (1) Population—children and adolescents aged five to 19 years, as defined by the World Health Organization for health and nutrition contexts [32] (when the study reported an age range from zero to five years, only the results for five-year-old children were considered, and when mean age values were presented, that mean age had to be between five and nineteen years). This broad age range allows for the inclusion of reviews that address children and/or adolescents and facilitates the identification of changes in movement behaviors during the transition from childhood to adolescence. (2) Concept—studies aimed at summarizing evidence on integrated 24-hour movement. (3) Context—all contexts were considered. (4) Types of studies—systematic reviews, scoping reviews, theoretical trials, or narrative reviews. (5) Studies were included if they were written in Portuguese, English, or Spanish.

The exclusion criteria were as follows: duplicated articles, original articles of any design, abstracts, books, book chapters, expert opinions or viewpoints, articles not available in full text (even after contacting the authors), and studies that investigated 24-hour movement behaviors separately or that only analyzed the integration of two behaviors.

### 2.4. Selection of Evidence Sources

**Figure 1:**
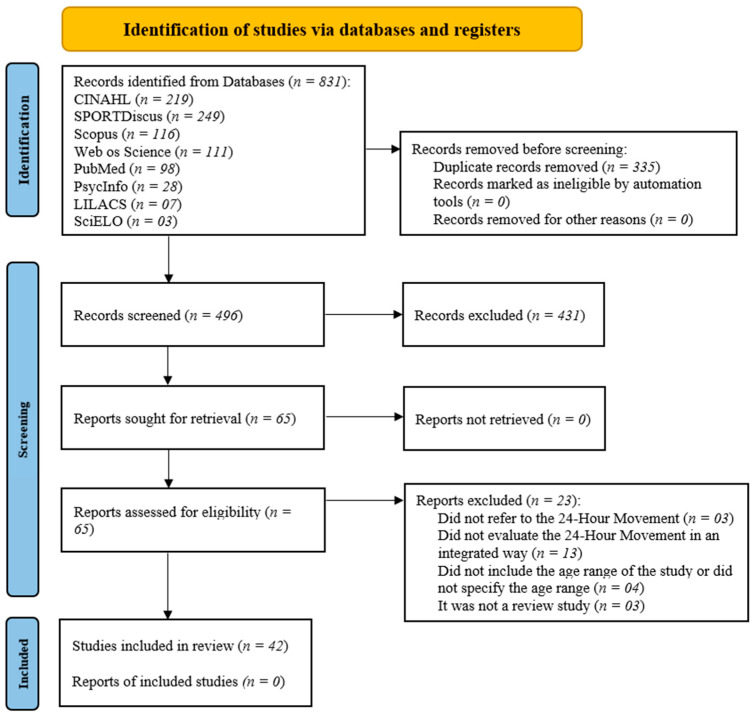
Preferred Reporting Items for Systematic Reviews and Meta-Analyses Extension for Scoping Reviews flow diagram.

Two reviewers (AFS and PCM) independently screened the databases, removed duplicate articles, and excluded those that did not meet the inclusion criteria. In cases of disagreement, a third researcher (DASS) was consulted. The reference lists of the included articles were reviewed, and a list detailing the exclusions was compiled (Table S2), which is summarized in the flow diagram in Figure 1 [33].

Rayyan software (https://rayyan.ai/users/sign_in, accessed on 3 May 2023) [34] (Qatar Computing Research Institute, Doha, Qatar) was used to manage the studies, identify and remove duplicates, and organize the results from each database simultaneously in a blinded system. After this process, the selected articles were exported to the reference manager Zotero^®^, version 5.0 (Roy Rosenzweig Center for History and New Media, Fairfax, VA, USA).

### 2.5. Synthesis of Results

The main characteristics and outcomes of the review studies were synthesized using Microsoft Excel software, version 14.7.7 (Microsoft, 2010, Washington, DC, USA), and the ©Word Clouds website (https://www.wordclouds.com/, accessed on 17 February 2025) to create a word cloud. Therefore, when such results were not presented directly, it was necessary to reformat them using calculations that considered only the specific sample, based on information obtained from the review results, tables, and/or appendix materials. The information was categorized into six categories depending on the aims of the studies: (1) “24-Hour Movement Guidelines”; (2) “Measurement of 24-Hour Movement”; (3) “Adherence to 24-Hour Movement Guidelines”; (4) “Changes in Time Spent in 24-Hour Movement Behaviors”; (5) “Health and 24-Hour Movement”; and (6) “Other 24-Hour Movement Associations” (Table 1 and Tables S3 and S4)”.

## 3. Results

### 3.1. Study Selection Process

The literature search identified 831 publications. After the removal of duplicate studies, 496 remained. Following the screening of titles and abstracts, 76 studies (15.3%) were deemed eligible for full-text review, of which 23 studies (4.6%) were excluded for not meeting the eligibility criteria (Table S2). A total of 42 reviews (8.5%) were ultimately included in the current scoping review [1,3,7,8,11,13,14,15,16,17,18,19,20,21,22,23,24,25,28,35,36,37,38,39,40,41,42,43,44,45,46,47,48,49,50,51,52,53,54,55,56,57] (Figure 1).

### 3.2. Characteristics of the Studies

The characteristics of the 42 included reviews are summarized in Table 1 and Tables S3 and S4, according to their respective aims. Four reviews (9.5%) addressed the aim “24-Hour Movement Guidelines” [11,16,40,43] and six studies (14.3%) reported on the aim “Measurement of 24-Hour Movement” [15,19,21,39,42,46]. Five reviews (11.9%) covered the aim “Adherence to 24-Hour Movement Guidelines” [1,14,44,50,54]. Five reviews (11.9%) discussed the aim “Changes in Time Spent in 24-Hour Movement Behaviors” [13,24,37,38,49]. Nineteen reviews (45.3%) focused on the aim “Health and 24-Hour Movement” [3,7,8,17,18,20,22,28,35,36,41,45,47,48,51,52,53,56]. Three reviews (7.1%) aimed “Other 24-Hour Movement Associations” [23,25,55]. Nine reviews (21.4%) [7,20,28,35,41,47,51,53,57] aimed to both synthesize information about “Health and 24-Hour Movement” and “Adherence to 24-Hour Movement Guidelines” and relate this to the 24-Hour movement guidelines. One review (2.4%) was aimed at “Changes in Time Spent in 24-Hour Movement Behaviors” and “Health and 24-Hour Movement” [13], and one (2.4%) was aimed at “Other 24-Hour Movement Associations” and “Adherence to 24-Hour Movement Guidelines” [23]. Given the robustness of the results presented for the “Health and 24-Hour Movement” aim, this study was classified solely under the main aim.

Regarding the types of review, eighteen systematic reviews (42.9%) [3,7,11,15,17,18,21,22,24,25,39,41,42,46,48,50,53,56], ten systematic reviews with meta-analysis (23.8%) [1,20,23,35,36,37,45,49,54,55], eight scoping reviews (19.0%) [8,13,14,19,40,43,44,52], five narrative reviews (11.9%) [16,28,38,47,51], and one umbrella review (2.4%) [57] were reported. The type of review and the aim researched in each study compiled in this scoping review of reviews are presented in Table S4.

**Figure 2:**
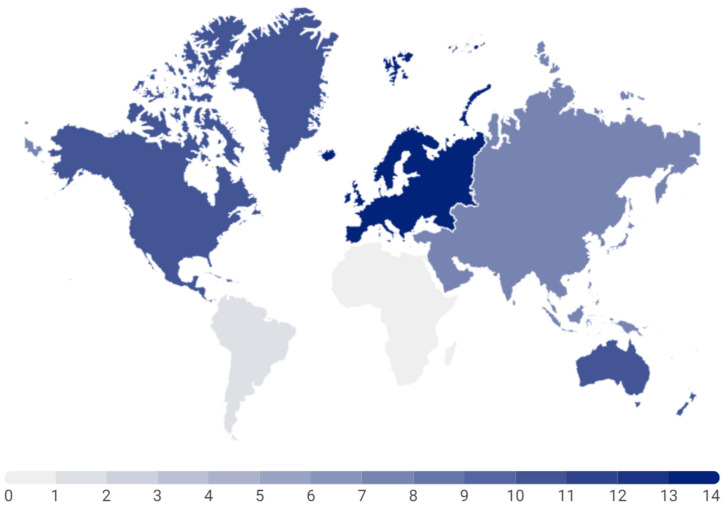
Geographic distribution of mapped reviews, according to the affiliation of the first author (*n* = 42).

The earliest mapped review was the systematic review published in 2016 by Saunders et al. [3], and the most recent reviews were six systematic reviews [25,42,48,50,53,56], three scoping reviews [13,14,19], and one umbrella review [57]—all published in 2024—marking this the year with the highest number of reviews exploring the theme of 24-hour movement in children and adolescents. These reviews covered the aims of “Health and 24-Hour Movement” [20,35,48,53,56,57], “Adherence to 24-Hour Movement Guidelines” [14,50,54], “Measurement of 24-Hour Movement” [19,42], “Changes in Time Spent in 24-Hour Movement Behaviors” (11.9%) [37,49], and “Other 24-Hour Movement Associations” [23,25,55] (Table 1 and Tables S3 and S4). Based on the affiliation of the first author of each review, fourteen reviews were led in Europe (33.3%) [1,11,15,22,28,36,37,38,39,40,42,45,46,51] (Croatia [11], Scotland [22], Spain [1,45], France [28,40,51], the Netherlands [15,46], Portugal [36,39], Slovenia [42], Switzerland [38], and Ireland [37]); ten in North America (23.8%) [3,7,8,16,21,41,47,53,56,57] (Canada [3,7,8,41,47,56] and the United States of America [16,21,53,57]); ten in Oceania (23.8%) [13,17,18,19,23,24,25,43,50,52] (Australia); seven in Asia (16.7%) (China [14,20,35,44,49,54,55]); and one in South America (2.4) (Brazil [48]) (Figure 2).

The age range of the samples from the original studies included in the mapped systematic reviews varied from 0 [7,11,15,25,39] to ≥65 years [7]. In the scoping reviews, it ranged from 0 [8,43] to 17 years [8], and in the narrative reviews, it varied from 0 [16] to 65 years [51]. There was significant variability in the age range of the samples from the original studies used to conduct the reviews. Additionally, three scoping reviews [52], two narrative reviews [28,38], two systematic reviews [53,56], one systematic review and meta-analysis [20], and the umbrella review [57] did not report the specific age range of the studies used for the review but mentioned that the sample or target audience was children and adolescents (Table 1 and Table S3).

**Figure 3:**
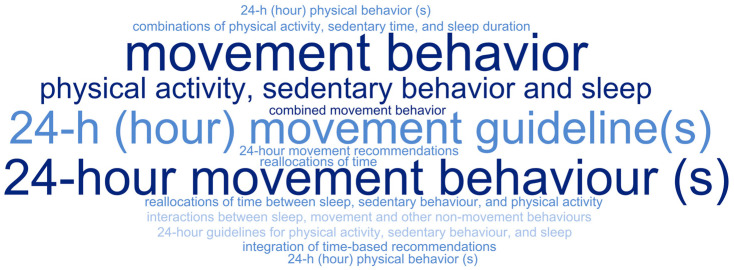
Word cloud with the terms adopted in the titles of the reviews mapped to report the results related to 24-hour movement.

**Figure 4:**
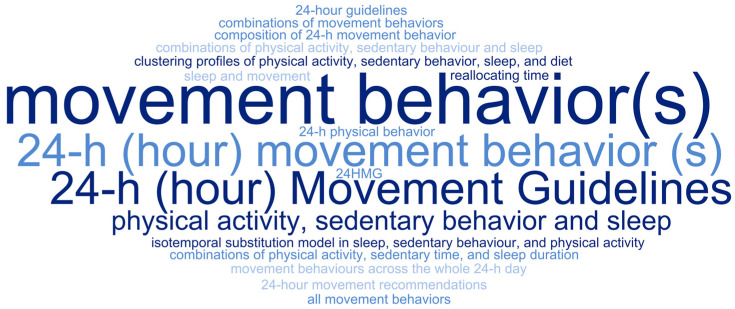
Word cloud with the terms adopted in the objectives of the reviews mapped to report the results related to 24-hour movement.

The reviews analyzed adopted different terms in their titles (Figure 3) and objectives (Figure 4). The terms “24-hour movement behavior(s)” (*n* = 15) and “24-hour movement guideline(s)” (*n* = 9) were the most frequently used in the titles. Meanwhile, the terms “24-hour movement behavior(s)” (*n* = 13) and “movement behavior(s)” (*n* = 7) were most commonly adopted in the objectives of the reviews. Overall, the term “24-hour movement behavior(s)” was the most frequently used, appearing in 28 out of the 42 studies.

Word cloud: 24-hour movement behavior(s) = 15; 24 h (hour) movement guideline(s) = 9; movement behavior = 4; physical activity, sedentary behavior, and sleep = 3; 24 h (hour) physical behavior(s) = 1; 24-hour guidelines for physical activity, sedentary behavior, and sleep = 1; combinations of physical activity, sedentary time, and sleep duration = 1; 24-hour movement recommendations = 1; combinations of physical activity, sedentary behavior, and sleep = 1; 24 h (hour) physical behavior(s) = 1; combined movement behavior = 1; integration of time-based recommendations = 1; interactions between sleep, movement, and other non-movement behaviors = 1; reallocations of time between sleep, sedentary behavior, and physical activity = 1; reallocations of time = 1.

Word cloud: 24 h (hour) movement behavior(s) = 13; movement behavior(s) = 7; 24 h (hour) movement guidelines = 6; physical activity, sedentary behavior and sleep = 2; 24 h physical behavior = 1; 24-hour guidelines = 1; all movement behaviors = 1; combinations of movement behaviors = 1; combinations of physical activity, sedentary behavior, and sleep = 1; combinations of physical activity, sedentary time, and sleep duration = 1; composition of 24 h movement behavior = 1; isotemporal substitution model in sleep, sedentary behavior, and physical activity = 1; movement behaviors across the whole 24 h day = 1; clustering profiles of physical activity, sedentary behavior, sleep, and diet = 1; 24-hour movement recommendations = 1; 24 HMG = 1; reallocating time = 1; sleep and movement = 1.

The research proposals followed by the outcomes of the reviews are presented according to the five aims and are described in Table S4.

### 3.3. Aim: “24-Hour Movement Guidelines”

As a result of the four reviews [11,16,40,43] that addressed the aim “24-Hour Movement Guidelines” in children and adolescents, national 24-hour movement behavior guidelines have been identified in Australia [11,16,43], Canada [11,16], South Africa [11,43], and New Zealand [11]. The Canadian guidelines were the first to integrate recommendations for physical activity, sedentary behavior, and sleep [16]. Nearly all existing recommendations for 24-hour movement behaviors for children and adolescents are based on the Canadian guidelines, as they were formulated in accordance with the currently available scientific evidence [11] (Table S4).

### 3.4. Aim: “Adherence to the 24-Hour Movement Guidelines”

As a result of the 5 reviews [1,14,44,50,54], 203 original studies were included, of which 91 assessed 24-hour movement in children and/or adolescents. Additionally, one systematic review did not report specific results for the combination of the three movement behaviors in children and adolescents [50] (Table S4).

Concerning overall adherence to the 24-hour movement behavior guidelines, adherence was 7.12% in the sample aged 3 to 18 years, with higher rates in children (10.31%) compared to adolescents (2.68%) [1]. One review that focused only on the Chinese population reported that the prevalence of adherence to the 24-hour movement guidelines varied from 0.3% to 26.1% in children and adolescents [14]. In subgroup analyses, overall adherence to the movement guidelines was lower among female children (6.89%) compared to male children (11.05%), but no differences were reported between sexes among adolescents (female: 6.92% and male: 8.61%) [1] (Table S4).

Regarding children and adolescents with disabilities, 16.0% did not meet any of the 24-hour movement behaviors, and overall adherence was 7.0% [54]. Specifically, in the Chinese population, children with autism spectrum disorder had an overall compliance rate of 16.2%, while children and adolescents with intellectual disabilities had a compliance rate of 17.5% [14] (Table S4).

Geographically, lower prevalences of adherence to the 24-hour movement guidelines were identified in children and adolescents from South America and Asia [1]. Meta-regression models demonstrated that overall adherence to the 24-hour movement guidelines was positively associated with the Human Development Index (HDI) of the country [1]. Furthermore, specifically during the COVID-19 pandemic, the prevalence of meeting the 24-hour movement behavior guidelines was below 5.0% [44] (Table S4).

### 3.5. Aim: “Measurement of 24-Hour Movement”

As a result of the six reviews that addressed the aim “Measurement of 24-Hour Movement” in children and adolescents [15,19,21,39,42,46], two systematic reviews identified no questionnaires specifically designed to assess 24-hour movement in an integrated manner [39,42]. Furthermore, two systematic reviews found no valid and reliable questionnaires specifically designed to assess 24-hour movement or electronic ecological momentary assessment methodologies [21,46]. Regarding accelerometer-based methods for assessing 24-hour movement in children, valid cut-off points for the use of accelerometers on the hip and wrist were identified for assessing sedentary behavior, light physical activity, and moderate-to-vigorous physical activity. Additionally, cut-off points for the use of wrist-worn accelerometers to assess sleep in preschoolers were identified, according to a systematic review [15]. In this context, one scoping review indicated that line graphs were the most common data visualization method used to present temporal patterns [19]. Nonetheless, reliable and/or valid accelerometer-based methods for assessing 24-hour movement in an integrated manner still need to be developed [15] (Table S4).

### 3.6. Aim: “Changes in Time Spent in 24-Hour Movement Behaviors”

Five reviews addressed the aim “Changes in Time Spent in 24-Hour Movement Behaviors” among children and/or adolescents: one narrative review [38], one systematic review [24], one scoping review [13], and two systematic reviews with meta-analysis [37,49]. The narrative review did not specify the number of studies in the researched topic [38], and one systematic review with meta-analysis did not present results specifically for the combination of the three movement behaviors in children and/or adolescents [37]. One systematic review with meta-analysis demonstrated that physical activity interventions targeting sedentary behavior and sleep showed no differences between both groups, while screen time interventions targeting physical activity showed mixed results, and in screen time interventions targeting sleep, no significant changes were reported [49]. One systematic review reported that the movement restrictions imposed during the pandemic period led to reduced adherence to the 24-hour movement guidelines among children and adolescents [38]. Furthermore, one scoping review about reallocations time between sleep, sedentary behavior, light physical activity, and moderate-to-vigorous physical activity were similarly associated with health outcomes [13]. In general, cross-sectional results showed that reallocating time to moderate-to-vigorous physical activity from any behavior was favorably associated with health, while reallocating time away from this activity to any other behavior was unfavorably associated with health [13]. Some beneficial associations were observed when reallocating time from sedentary behavior to light physical activity and sleep, although with a lower impact compared to reallocations involving moderate-to-vigorous physical activity [13]. However, many studies found no significant associations, and most longitudinal studies did not identify a relationship between time reallocations and health [13] (Table S4).

### 3.7. Aim: “Health and 24-Hour Movement”

Of the nineteen reviews that addressed the aim “Health and 24-Hour Movement”, nine systematic reviews [3,7,8,17,18,20,22,28,35,36,41,45,47,48,51,52,53,56,57], four systematic reviews with meta-analysis [14,35,36,45], three narrative reviews [28,47,51], two scoping reviews [8,52], and one umbrella review [57] reported studies that assessed 24-hour movement in children and adolescents. However, three systematic reviews failed to map any studies that had evaluated 24-hour movement associated with health indicators in Arabic-speaking countries [18], the impact on mental and physical health outcomes in adolescents diagnosed with type 1 diabetes [22], and the relationships between climate change, 24-hour movement behaviors, and health [56]. Additionally, two studies did not specify the number of studies mapped [28,47] (Table S4).

Until the year 2018, the health outcomes most studied in relation to isotemporal substitutions of movement behaviors in children and adolescents were body adiposity and performance in physical fitness tests [52]. However, since the introduction of the continuous model of 24-hour movement behaviors, an increasing number of studies have examined the relationships between the composition of 24-hour movement behaviors and various health indicators in children and adolescents [28]. As a result of this aim, the authors of several reviews reported that school-aged children and adolescents who exhibited healthier combinations of movement behaviors (reduced sedentary behavior, high levels of physical activity, and prolonged sleep duration) generally showed more desirable measures of body adiposity [35] and obesity/overweight [36,45], a lower risk of high body mass index or body mass index z-scores [35,36,45], lower waist circumference [35,45], a lower prevalence of obesity [7,47], lower body fat [35,45], healthy eating patterns [7,51], health benefits [57], good cardiometabolic health [3,7], good cardiometabolic biomarkers [35], high aerobic fitness [35] and muscular physical fitness [17], high speed [35], high agility [35], good psychological/mental well-being [35], fewer emotional problems [35], good quality of life [35], good indicators of mental health [8,17,41,53], high academic performance [17], adequate executive/cognitive function [17], appropriate social and emotional behaviors [7], lower anxiety [35], and fewer depression symptoms [35] compared with those who reported less healthy combinations of movement behaviors (Table S4).

Regarding children and adolescents with an intellectual disability or attention deficit/hyperactivity disorder, meeting the 24-hour guidelines was not significantly associated with the odds of being overweight or obese but was associated with lower odds of cognitive and social difficulties, as well as a higher flourishing level, compared to meeting none or only one of the guidelines in youths with an intellectual disability or attention deficit/hyperactivity disorder [20]. Reallocating time from physical activity and sedentary behavior to sleep duration decreased body mass index in children and adolescents with autism spectrum disorder [20]. Furthermore, reduced sedentary behavior, high levels of physical activity, and prolonged sleep duration were not the most prevalent clusters among the cluster studies [48]. However, some studies noted a fragility in the quantity and overall quality of the evidence available from research [3,18,28,41,52] (Table S4).

### 3.8. Aim: “Other 24-Hour Movement Associations”

Three reviews, one systematic review [25], and two systematic reviews with meta-analysis [23,55] reported studies whose objectives did not align with any of the previous groupings. Therefore, they were included under the classification “Other 24-Hour Movement Associations”. Two reviews failed to map any studies that had evaluated 24-hour movement associated with the effectiveness of eHealth interventions on 24-hour movement behaviors [55] and the associations between pollution measures (air, water, land, and noise) and 24-hour movement behavior among children from birth to 12 years of age [25] (Table S4).

Concerning the association between adherence to the 24 h movement guidelines and academic-related outcomes in children and adolescents, a meta-analysis suggests a small relationship between adherence to all three recommendations and academic achievement compared to those who did not adhere to any recommendations [23]. Not adhering to the 24-hour movement guidelines was associated with poor executive functions, shifting efficiency, and non-preservative errors, compared to those who adhered to the 24-hour movement guidelines. Additionally, adhering to the 24-hour movement guidelines, when compared to no adherence to any, was positively associated with composite cognition cognitive function at baseline, but not during follow-up, in a longitudinal study [23]. However, due to the limited number of studies included and their low quality, this association should be interpreted with caution [23] (Table S4).

## 4. Discussion

The main findings of this review are as follows: (1) There are national guidelines for 24-hour movement behaviors for children and/or adolescents only in Australia, Canada, New Zealand, and South Africa. (2) There is a notable lack of valid and reliable measurement tools for assessing 24-hour movement behaviors, limiting the accuracy and comparability of research findings in this field. (3) Adherence to the 24-hour movement guidelines is low worldwide, including among preschool populations, healthy children, and adolescents, as well as those with disabilities. (4) Interventions targeting screen time and physical activity yielded mixed results, but reallocating time to moderate-to-vigorous physical activity from any other behavior(s) was consistently associated with health benefits. During the COVID-19 pandemic, there was a decline in this activity, accompanied by an increase in screen time and total sleep duration. (5) School-aged children and adolescents who exhibited healthier combinations of movement behaviors (reduced sedentary behavior, higher levels of physical activity, and extended sleep duration) demonstrated better mental and physical health indicators, including lower adiposity, improved cardiometabolic health, better psychological well-being, and higher cognitive and academic performance; however, some studies highlighted limitations in the quantity and quality of available evidence. (6) There is a possible relationship between adherence to 24-hour movement behaviors and cognitive function, pollution measures, and eHealth interventions. (7) There was variation in the terms used in the titles and objectives of the reviews to report 24-hour movement behaviors, indicating inconsistencies in how this topic was approached academically.

Over the past decade, reviews on 24-hour movement behaviors have primarily focused on summarizing evidence that established relationships between the integration of 24-hour movement behaviors and associations with health indicators [7,8,17,18,22,28,41,47,52]. This focus likely aimed to provide a foundation for guidelines developed for various age groups [2,12,58] and to justify the creation of additional national 24-hour movement guidelines [11]. Therefore, none of the reviews in this research aimed to summarize evidence from studies that investigated the three combined movement behaviors or proposed a suitable term to refer to the integration of the three movement behaviors that comprise the 24-hour day (physical activity, sedentary behavior, and sleep), as has been carried out for the terms individually [12,59,60].

### 4.1. 24-Hour Movement Guidelines

Only four countries have established national 24-hour movement guidelines for children and/or adolescents: Australia, Canada, New Zealand, and South Africa. The development of 24-hour movement guidelines is beneficial for healthcare professionals and educational settings as it helps to frame public health campaign messages and develop national, local, and institutional health policies [11]. However, it is noteworthy that Europe, despite accounting for 33.3% of the academic output of reviews related to 24-hour movement behaviors, as identified in the present review, reported only one review used to develop national 24-hour movement guidelines (i.e., Croatian guidelines) [11]. Parrish et al. [61] identified that European countries tend to follow three international guidelines about movement behaviors: the 2008 European Union Physical Activity Guidelines, which align with the World Health Organization’s (WHO) guideline; the 2012 Nordic Nutrition Recommendations, used by Iceland, Norway, and Sweden; and the WHO’s 2010 Global Recommendations on Physical Activity for Health. In contrast, on the African continent, in Mozambique and Nigeria, national guidelines following the Canadian guidelines were identified, but no documented and updated evidence was found [61]. This discrepancy highlights the variation in the adoption and documentation of health guidelines across different regions, potentially reflecting differences in public health priorities, resources, and the influence of regional health governance. The 2019 update of the WHO guidelines on physical activity, sedentary behavior, and sleep for children under five emphasized the need to consider the overall pattern of activity across the 24-hour period [62]. However, this update was not extended to the 5–17 age group [63]. Despite Canada being a pioneer in replacing national physical activity and sedentary behavior guidelines for children and adolescents with 24-hour movement guidelines, followed by Australia, their respective continents account for less than one-fourth of the mapped reviews. Another significant gap identified in this research is the absence of 24-hour movement guidelines in countries across South America, Central America, and Asia, which is also accompanied by a low number or complete lack of reviews on this topic.

### 4.2. Measurement of 24-Hour Movement

Understanding the temporal and contextual aspects of 24-hour movement behaviors is essential for assessing their health impact and identifying holistic lifestyle patterns [19]. In this context, line graphs are the most frequently used visualization method for presenting temporal patterns throughout the day or across weekdays and weekends in different populations and contexts [19]. However, measuring 24-hour movement requires specific protocols for accelerometers or validated and reliable questionnaires. The use of questionnaires to accurately assess 24-hour movement behaviors in children from zero to five years seems inadequate due to the sporadic and intermittent nature of these behaviors [46]. However, the development of high-quality studies aimed at creating questionnaires and conducting a comprehensive evaluation of their psychometric properties could improve the quality of proxy report questionnaires [46], as well as the outcomes derived from them.

Other reviews mapped in this study, whose primary objective was not specifically to measure 24-hour movement, also reported that movement behaviors were assessed without using a combined or unique measurement approach. These reviews documented studies using a variety of methods, including accelerometers [1,3,7,8,41], self-reported questionnaires [1,3,7,8,41], parent/caregiver reports [1,7,8,41], pedometers [3,7], Global Positioning Systems (GPSs) [8], and smartphone-linked apps [8]. Regarding the use of accelerometers, although specific placements that differentiate some movement behaviors have been identified, specific placements and axes that provide the most accurate measurement of 24-hour movement have not yet been established [15]. Thus, an ideal measurement protocol to assess 24-hour movement in children and adolescents, whether through valid and reliable questionnaires [46], the effective use of accelerometers [15], or the electronic ecological momentary assessments methodology [21], has not yet been identified. Therefore, studies that delve into the development of specific methods or that improve current instruments for the adequate measurement and analysis of combined 24-hour movement behaviors appear to be a necessity in this area [14,15].

### 4.3. Adherence to 24-Hour Movement Guidelines

The variations in prevalence between preschool populations, healthy children, and adolescents, or those with disabilities, may be attributed to the use of different instruments to assess 24-hour movement behaviors across age groups [1,14,44,54]. However, the general prevalence of meeting the 24-hour movement recommendations in these populations is low and declined during the COVID-19 pandemic, with compliance falling below 5%, highlighting the need to mitigate its lasting negative impacts on youth [44]. This generally low prevalence can be attributed to various factors, including the technological revolution of the 21st century, particularly the popularity of electronic devices, which has reduced the time available for engaging in healthy behaviors [1,20]. Additionally, environmental factors, such as rapid urbanization, urban violence, changes in transportation modes, family characteristics, including the lack of social support from friends and family, and increased pressure from work and school, may also be linked to adherence to the guidelines [7,14,38].

In this context, to increase adherence to the 24-hour movement guidelines and to ensure they have a positive impact on public health, approaches are needed that include active dissemination and sensitivity towards the implementation of these guidelines [2]. Such efforts should involve interdisciplinary collaboration and entail changes in policies and service provisions within society [2]. Furthermore, the development of 24-hour movement guidelines tailored to the needs of each country and population, as well as research on the effectiveness of interventions designed to promote all three movement behaviors, could be effective in increasing these prevalences [14,44]. For example, specific impairments or functional limitations associated with certain disabilities may directly affect an individual’s ability to participate in physical activity [54]. Another example is that the 24-hour movement behaviors of LGBTQA+ youth may contribute to health disparities compared to non-LGBTQA+ youth, but the lack of studies and methodological limitations hinder the understanding of these patterns, particularly regarding sedentary time and sleep [50]. Therefore, additional research and interventions are needed to overcome obstacles and promote physical activity by developing accommodating environments, providing inclusive opportunities, implementing educational initiatives [50,54], and enhancing access to recreational facilities, outdoor play, and organized activities, especially amid ongoing public health challenges [44]. These comprehensive strategies could better facilitate the integration of healthy movement behaviors into the daily lives of children and adolescents, potentially reversing current trends in inactivity and sedentary lifestyles [2].

### 4.4. Changes in Time Spent in 24-Hour Movement Behaviors

Any increase in the time dedicated to a movement behavior that comprises the 24-hour day is compensated by a reduction in time spent on another behavior, and how this time is distributed directly impacts health [13,16]. However, reallocating time to moderate-to-vigorous physical activity may bring greater benefits compared to other 24-hour movement behaviors, requiring proportionally larger changes [13]. Moreover, when moderate-to-vigorous physical activity occurs during work, the benefits are not evident, highlighting the importance of the context in which the activity takes place (leisure vs. work) [13]. This may explain the difficulty researchers and public health campaigns face in encouraging sustainable increases in moderate-to-vigorous physical activity [13]. On the other hand, more subtle reallocations, such as reducing sedentary time in favor of light physical activity or increasing sleep duration, can serve as a starting point for behavior changes [13]. In a review about the overflow effects of movement behavior change interventions for children and adolescents, physical activity interventions reduced sedentary behavior, especially when these interventions were effective in increasing physical activity levels [49]. Conversely, interventions focused on sedentary behavior led to increases in time spent standing, with no effects observed on other movement behaviors, reinforcing the importance of a holistic approach based on the 24-hour framework [49]. In this regard, further multi-behavior interventions are needed to explore strategies for achieving optimal health outcomes [49].

Specifically, during the COVID-19 pandemic, there was a reduction in moderate-to-vigorous physical activity, an increase in sedentary behavior—primarily screen time—and an increase in total sleep duration, leading to a reallocation of time among movement behaviors due to restrictions [37,38]. These changes were triggered by various factors, such as adjustments in school schedules and activities [38]. Additionally, limitations on opportunities for participation in structured exercises at sports clubs resulted in the reallocation of physical activity time to sedentary behavior, particularly screen time [38]. The reallocation of time during this period of movement restrictions may have integrated into the lifestyle of children and adolescents, persisting even after the end of pandemic containment measures, with potential long-term negative impacts [38,44].

Additionally, no studies have assessed changes in the time use composition of the three movement behaviors during the school transition, limiting the understanding of these changes in child development [24]. Therefore, while there is growing scientific interest in the associations between time reallocation among 24-hour movement behaviors, gaps remain that need further exploration [52].

### 4.5. Health and 24-Hour Movement

The relationship between movement behaviors and health has been fundamental in developing 24-hour movement guidelines, influencing their creation, revision, and expansion in different countries [2]. Additionally, emerging statistical approaches based on the compositional paradigm have helped identify optimal time distributions for movement behaviors throughout the day, offering valuable insights for refining guidelines and maximizing health benefits [47].

Despite these advances, several gaps still limit definitive conclusions. The predominance of cross-sectional studies [7,8,17,20,22,41,52] and the limited number of longitudinal or intervention-based studies [3,41] hinder the understanding of causality between movement behaviors and health outcomes. Additionally, reliance on self-reported measures [8,20,41,47], and the dichotomization of variables related to 24-hour movement behaviors [17] affect data accuracy, potentially distorting findings. There is also considerable variability in the quality of mapped studies, ranging from very low to high [7,8,18,20,22,24,41,52].

Beyond methodological challenges, low adherence to the 24-hour movement highlights the need to explore environmental, technological, and social factors influencing these behaviors [14]. Rapid urbanization, increased screen time, and evolving lifestyle patterns have contributed to reduced physical activity and prolonged sedentary behavior [25]. From Bronfenbrenner’s bioecological perspective, analyzing how family, school, and societal systems shape these behaviors can offer valuable insights for improving guideline adherence and health outcomes [13,16,64].

To address these gaps, future research should prioritize longitudinal designs and intervention studies [15]. Integrating accelerometry, activity logs, and machine learning can improve measurement accuracy [21]. Expanding studies to underrepresented regions such as Latin America, Africa, and parts of Asia is crucial for making guidelines more inclusive and culturally relevant [11].

Lastly, cross-sectoral strategies—including public health campaigns, school policy changes, and parental education—can be key to increasing adherence and promoting healthier habits from childhood [2,44,47,55]. Combining these strategies with research advancements and improved assessment methods is essential to strengthening the impact of 24-hour movement on child and adolescent health.

### 4.6. Other 24-Hour Movement Associations

Other associations with 24-hour movement behaviors have been investigated, including the relationship between adherence to these behaviors and cognitive function [23]. Indeed, growing evidence suggests that the brain is sensitive to movement behaviors [65]. Structural brain assessments have highlighted that adolescents who meet the 24-hour movement guidelines exhibit greater cortical and subcortical gray matter volumes, which are essential for processing and transmitting information within the brain [66]. Moreover, gray matter density typically increases from birth to early puberty and then declines throughout adolescence. Therefore, failure to adhere to movement guidelines during childhood and adolescence may lead to premature reductions in brain structure, potentially resulting in cognitive impairments that persist throughout life. However, due to the limited number of studies and their heterogeneity, these findings should be interpreted with caution [23].

Additionally, environmental pollution measures (air, water, land, and noise) [25] and eHealth interventions [55] have also been subjects of investigation. Although the available evidence remains limited, it already indicates that environmental factors are important variables to be explored [25]. Regarding eHealth interventions, no study has examined movement behaviors in an integrated manner. However, research focusing on individual movement behaviors has been conducted, yielding promising and effective results for this approach [67,68].

### 4.7. Terms Used to Report 24-Hour Movement Behaviors

The term “24-hour movement behaviors” was the most frequently referenced term in the titles and objectives of 28 reviews. However, the mapped reviews cited at least 22 different terms. No specific cause was identified for the use of each term, such as its frequency in a particular location or year of publication. This discrepancy may be explained by the fact that research in this area began to intensify in 2016, following the release of the Canadian guidelines [2]. Nevertheless, it is important to acknowledge the multidimensional nature of each movement behavior and ensure that these terms accurately reflect the construct of interest. Standardizing terminology would facilitate more consistent reporting, enable better comparisons across studies, and strengthen the overall findings. Consistency in terminology is essential for synthesizing research across studies and for fostering a coherent understanding that can be effectively communicated within the scientific community [3].

### 4.8. Interconnections Among the Review Aims Related to 24-Hour Movement

Low adherence to the 24-hour movement guidelines may be attributed to the lack of standardized, reliable, and validated tools for holistically measuring these behaviors and obtaining results that accurately reflect real-world movement patterns [14,15,19,21,39,42]. This limitation also poses significant challenges for the formulation of public health policies and the implementation of effective public health interventions, especially in lower-resource countries [11,14,43].

The lack of clear protocols for using accelerometers and validated questionnaires within the 24-hour movement paradigm hinders the collection of consistent data on physical activity, sedentary behavior, and sleep patterns. This inconsistency compromises the development and adaptation of national guidelines, limiting their effectiveness in promoting movement behaviors [15,19,21,35,42,46].

Additionally, environmental factors such as rapid urbanization and pollution influence movement behaviors, highlighting the need for a more integrated and contextualized approach [25]. From the perspective of Bronfenbrenner’s bioecological model, understanding how different systems (family, school, society) shape these behaviors can provide valuable insights for developing targeted strategies to enhance adherence to movement recommendations and improve health outcomes [13,16,64]. In this context, comprehensive interventions—such as public health campaigns, modifications to school policies, and educational programs for parents—may be essential in reversing current trends in physical inactivity and sedentary behavior among children and adolescents [1,2,3,8,17,40,44,47,55].

Finally, since 2016, there has been a significant increase in the number of reviews on 24-hour movement in children and adolescents, particularly between 2023 and 2024. However, this growth in scientific research does not appear to be accompanied by the development of new 24-hour movement guidelines, which may negatively impact adherence to recommendations and, consequently, the health outcomes of children and adolescents.

### 4.9. Positive Points

This review presents several strengths, including the comprehensive range of databases consulted and the rigorous peer review process, which adhered to strict methodological criteria. Additionally, although specific languages were pre-designated, language was not used as a filtering criterion, enabling engagement with authors whose reviews were published in languages beyond those initially designated. Another notable strength of this work is the detailed assessment of the methodological quality of the mapped systematic reviews (Table S5), which enhances the overall understanding of the quality of the available evidence. 

### 4.10. Limitations

Among the limitations of this research is the absence of information from grey literature, although such sources may also be constrained by issues related to indexing, reporting, and the potential for result modification [3]. The capacity to translate and contact authors suggests that multiple languages could have been incorporated from the onset of the methodological design. Additionally, the variation in terminology used to describe 24-hour movement behaviors across the reviews complicated broader comparisons between studies and hindered the interpretation of results. Lastly, only studies explicitly designer to analyze 24-hour movement were considered; thus, studies that presented findings related to 24-hour movement but did not explicitly aim to analyze it may not have been included in this review.

### 4.11. Featured Application

These findings underscore the need for: (1) broader implementation of 24-Hour Movement Guidelines as a health promotion strategy in pediatric settings, particularly in low- and middle-income countries, where such guidelines are currently lacking; (2) increased standardization of measurement instruments, including validated protocols for accelerometry and questionnaires, to improve the assessment of 24-Hour Movement Behaviors; and (3) further research investigating the relationships between adherence to 24-Hour Movement Guidelines and cognitive function, environmental pollution measures, and eHealth interventions.

## 5. Conclusions

This review mapped the scientific evidence that summarized key findings regarding 24-hour movement in children and adolescents. We found that, over the past few decades, half of the mapped reviews highlighted the relationship between integrated 24-hour movement behaviors and health indicators, with significant outcomes in two-thirds of the cases, suggesting that healthier integration of the three movement behaviors is positively associated with the overall health of children and adolescents.

Despite this, there was low adherence to the 24-hour movement guidelines; the COVID-19 pandemic exacerbated this situation, and national guidelines that emphasize the importance of children and adolescents meeting the 24-hour movement criteria are observed in few countries. Furthermore, there is a noted lack of measurement instruments with valid and reliable protocols specifically for assessing 24-hour movement, as well as standardization in the use of the term to report the integration of the three movement behaviors. Additionally, the limited development of global 24-hour movement guidelines may reflect the lack of specific strategies to enhance adherence. Future research should explore approaches such as educational campaigns, school and community involvement, and culturally adapted recommendations to facilitate implementation in diverse contexts.

Therefore, high-quality research is needed not only to develop quality assessment protocols to understand the long-term impact of 24-hour movement across different age groups but also to identify and implement effective interventions. This is particularly relevant in countries not mapped in this review and those without established 24-hour movement guidelines, where the lack of structured recommendations may hinder adherence and, consequently, health outcomes in children and adolescents.

## Figures and Tables

**Table 1:** Characteristics of the reviews included, according to the type of review and the aim researched (*n* = 42).

Characteristics	Aim Researched (*n* = 42; 100%)						
	24-Hour Movement Guidelines (*n* = 04; 9.5%)	Measurement of 24-Hour Movement (*n* = 06; 14.3%)	Adherence to 24-Hour Movement Guidelines (*n* = 05; 11.9%)	Changes in Time Spent in 24-Hour Movement Behaviors (*n* = 05; 11.9%)	Health and 24-Hour Movement (*n* = 19; 45.3%)	Other 24-Hour Movement Associations (*n* = 03; 7.1%)	Total Result of 24-Hour Movement Aims (*n* = 42; 100%)
**Type of review**	***n* (%)**	***n* (%)**	***n* (%)**	***n* (%)**	***n* (%)**	***n* (%)**	***n* (%)**
Narrative reviews	01 (2.4)	-	-	01 (2.4)	03 (7.1)	-	05 (11.9)
Systematic reviews	-	05 (11.9)	01 (2.4)	01 (2.4)	09 (21.4)	01 (2.4)	18 (42.9)
Systematic review with meta-analysis	-		02 (4.8)	02 (4.8)	04 (9.5)	02 (4.8)	10 (23.8)
Scoping reviews	03 (4.8)	01 (2.4)	02 (4.8)	01 (2.4)	02 (4.8)	-	08 (19.0)
Umbrella review	-	-	-	-	01 (2.4)	-	01 (2.4)
**Year**	***n* (%)**	***n* (%)**	***n* (%)**	***n* (%)**	***n* (%)**	***n* (%)**	***n* (%)**
2016	-	-	-	-	01 (2.4)	-	01 (2.4)
2017	-	-	-	-	01 (2.4)	-	01 (2.4)
2018	-	-	-	-	01 (2.4)	-	01 (2.4)
2019	01 (2.4)	-	-	-	-	-	01 (2.4)
2020	-	-	-	01 (2.4)	02 (4.8)	-	03 (7.1)
2021	-	-	-	01 (2.4)	01 (2.4)	-	02 (4.8)
2022	01 (2.4)	02 (4.8)	01 (2.4)	-	01 (2.4)	-	05 (11.9)
2023	02 (4.8)	02 (4.8)	01 (2.4)	-	06 (14.3)	-	11 (26.1)
2024	-	02 (4.8)	03 (7.1)	03 (7.1)	06 (14.3)	03 (7.1)	17 (40.5)
**Geographical location**	***n* (%)**	***n* (%)**	***n* (%)**	***n* (%)**	***n* (%)**	***n* (%)**	***n* (%)**
Asia	-	-	03 (7.1)	01 (2.4)	02 (4.8)	01 (2.4)	07 (16.7)
Europe	02 (4.8)	04 (9.5)	01 (2.4)	02 (4.8)	05 (11.9)	-	14 (33.3)
North America	01 (2.4)	01 (2.4)	-	-	08 (19.0)	-	10 (23.8)
Oceania	01 (2.4)	01 (2.4)	01 (2.4)	02 (4.8)	03 (7.1)	02 (4.8)	10 (23.8)
South America	-	-	-	-	01 (2.4)	-	01 (2.4)
**Sample**	***n* (%)**	***n* (%)**	***n* (%)**	***n* (%)**	***n* (%)**	***n* (%)**	***n* (%)**
Children	02 (4.8)	02 (4.8)	-	-	01 (2.4)	01 (2.4)	06 (14.3)
Adolescents	-	02 (4.8)	-	-	01 (2.4)	-	03 (7.1)
Both	02 (4.8)	02 (4.8)	05 (11.9)	05 (11.9)	17 (40.5)	02 (4.8)	33 (78.6)
**Database**	***n* (%)**	***n* (%)**	***n* (%)**	***n* (%)**	***n* (%)**	***n* (%)**	***n* (%)**
1	-	01 (2.4)	-	01 (2.4) *	-	-	02 (4.8)
3	-	03 (7.1)	01 (2.4)	-	02 (4.8)	-	06 (14.3)
4	01 (2.4) *	01 (2.4)	01 (2.4)	01 (2.4)	04 (9.5)	-	08 (19.0)
5	01 (2.4)	01 (2.4)	01 (2.4)	-	03 (7.1)	-	06 (14.3)
6	-	-	-	03 (7.1)	02 (4.8)	02 (4.8)	07 (16.7)
7	01 (2.4) *	-	02 (4.8)	-	02 (4.8)	-	05 (11.9)
8	-	-	-	-	03 (7.1)	01 (2.4)	04 (9.5)
14	-	-	-	-	01 (2.4) *	-	01 (2.4)
Did not specify	01 (2.4)	-	-	-	02 (4.8)	-	03 (7.1)
**Reviews that mapped original articles with results for the 24-hour movement**	***n* (%)**	***n* (%)**	***n* (%)**	***n* (%)**	***n* (%)**	***n* (%)**	***n* (%)**
Reviews that mapped	01 (2.4)	02 (4.8)	04 (9.5)	03 (7.1)	13 (30.9)	01 (2.4)	24 (57.1)
Revisions that did not map	01 (2.4)	03 (7.1)	01 (2.4)	01 (2.4)	04 (9.5)	02 (4.8)	12 (28.6)
Reviews that did not specify the number of studies mapped	02 (4.8)	01 (2.4)	-	01 (2.4)	02 (4.8)	-	06 (14.3)
**Result of reviews**	***n* (%)**	***n* (%)**	***n* (%)**	***n* (%)**	***n* (%)**	***n* (%)**	***n* (%)**
Significant	-	-	01 (2.4)	01 (2.4)	13 (30.9)	01 (2.4)	16 (38.1)
Just reported results	02 (4.8)	01 (2.4)	03 (7.1)	-	03 (7.1)	-	09 (21.4)
Not significant	-	-	-	01 (2.4)	-	-	01 (2.4)
Did not report results	02 (4.8)	05 (11.9)	01 (2.4)	03 (7.1)	03 (7.1)	02 (4.8)	16 (38.1)
**Total original articles mapped in reviews**	***n* (%)**	***n* (%)**	***n* (%)**	***n* (%)**	***n* (%)**	***n* (%)**	***n* (%)**
	05	08	91	8	194	10	316

* Google Scholar was considered gray literature even when the review itself classified it as a database. *n*: absolute value; %: percentage; -: information not reported in the review.

## Data Availability

The original contributions presented in this study are included in the article. Further inquiries can be directed to the corresponding author(s).

## Supplementary Materials

The following supporting information can be downloaded at: https://www.mdpi.com/article/10.3390/children12030260/s1, Table S1: Search strategy in different databases; Table S2: Articles removed according to the exclusion criteria; Table S3: Characteristics of included studies by aim group; Table S4: Results of included studies by aim group; Table S5: Quality and risk of bias in the systematic review studies and systematic reviews with meta-analysis of included studies.

## References

[1] Tapia-Serrano M.A., Sevil-Serrano J., Sánchez-Miguel P.A., López-Gil J.F., Tremblay M.S., García-Hermoso A. (2022). Prevalence of Meeting 24-Hour Movement Guidelines from Pre-School to Adolescence: A Systematic Review and Meta-Analysis Including 387,437 Participants and 23 Countries. J. Sport Health Sci..

[2] Tremblay M.S., Carson V., Chaput J.-P., Connor Gorber S., Dinh T., Duggan M., Faulkner G., Gray C.E., Gruber R., Janson K. (2016). Canadian 24-Hour Movement Guidelines for Children and Youth: An Integration of Physical Activity, Sedentary Behaviour, and Sleep. Appl. Physiol. Nutr. Metab..

[3] Saunders T.J., Gray C.E., Poitras V.J., Chaput J.-P., Janssen I., Katzmarzyk P.T., Olds T., Gorber S.C., Kho M.E., Sampson M. (2016). Combinations of Physical Activity, Sedentary Behaviour and Sleep: Relationships with Health Indicators in School-Aged Children and Youth. Appl. Physiol. Nutr. Metab..

[4] Carson V., Hunter S., Kuzik N., Gray C.E., Poitras V.J., Chaput J.-P., Saunders T.J., Katzmarzyk P.T., Okely A.D., Gorber S.C. (2016). Systematic Review of Sedentary Behaviour and Health Indicators in School-Aged Children and Youth: An Update1. Appl. Physiol. Nutr. Metab..

[5] Chaput J.-P., Gray C.E., Poitras V.J., Carson V., Gruber R., Olds T., Weiss S.K., Gorber S.C., Kho M.E., Sampson M. (2016). Systematic Review of the Relationships between Sleep Duration and Health Indicators in School-Aged Children and Youth. Appl. Physiol. Nutr. Metab..

[6] Poitras V.J., Gray C.E., Borghese M.M., Carson V., Chaput J.-P., Janssen I., Katzmarzyk P.T., Pate R.R., Connor Gorber S., Kho M.E. (2016). Systematic Review of the Relationships between Objectively Measured Physical Activity and Health Indicators in School-Aged Children and Youth. Appl. Physiol. Nutr. Metab..

[7] Rollo S., Antsygina O., Tremblay M. (2020). The Whole Day Matters: Understanding 24-Hour Movement Guideline Adherence and Relationships with Health Indicators across the Lifespan. J. Sport Health Sci..

[8] Lannoy L., Barbeau K., Vanderloo L.M., Goldfield G., Lang J.J., MacLeod O., Tremblay M.S. (2023). Evidence Supporting a Combined Movement Behavior Approach for Children and Youth’s Mental Health–A Scoping Review and Environmental Scan. Ment. Health Phys. Act..

[9] Falck R.S., Davis J.C., Li L., Stamatakis E., Liu-Ambrose T. (2022). Preventing the ‘24-Hour Babel’: The Need for a Consensus on a Consistent Terminology Scheme for Physical Activity, Sedentary Behaviour and Sleep. Br. J. Sports Med..

[10] Okely A.D., Ghersi D., Loughran S.P., Cliff D.P., Shilton T., Jones R.A., Stanley R.M., Sherring J., Toms N., Eckermann S. (2022). A Collaborative Approach to Adopting/Adapting Guidelines. The Australian 24-Hour Movement Guidelines for Children (5–12 Years) and Young People (13–17 Years): An Integration of Physical Activity, Sedentary Behaviour, and Sleep. Int. J. Behav. Nutr. Phys. Act..

[11] Jurakiÿ D., Pedišiÿ Ž. (2019). Croatian 24-Hour Guidelines for Physical Activity, Sedentary Behaviour, and Sleep: A Proposal Based on a Systematic Review of Literature. Medicus.

[12] Tremblay M.S., Chaput J.-P., Adamo K.B., Aubert S., Barnes J.D., Choquette L., Duggan M., Faulkner G., Goldfield G.S., Gray C.E. (2017). Canadian 24-Hour Movement Guidelines for the Early Years (0–4 Years): An Integration of Physical Activity, Sedentary Behaviour, and Sleep. BMC Public Health.

[13] Miatke A., Olds T., Maher C., Fraysse F., Mellow M.L., Smith A.E., Pedisic Z., Grgic J., Dumuid D. (2023). The Association between Reallocations of Time and Health Using Compositional Data Analysis: A Systematic Scoping Review with an Interactive Data Exploration Interface. Int. J. Behav. Nutr. Phys. Act..

[14] Huang J., Memon A.R., Bao R., Fan H., Wang L., Liu Y., Chen S., Li C. (2024). 24-H Movement Behaviours Research in Chinese Population: A Scoping Review. J. Exerc. Sci. Fit..

[15] Lettink A., Altenburg T.M., Arts J., van Hees V.T., Chinapaw M.J.M. (2022). Systematic Review of Accelerometer-Based Methods for 24-h Physical Behavior Assessment in Young Children (0–5 Years Old). Int. J. Behav. Nutr. Phys. Act..

[16] Vidmar A.P., Cáceres N.A., Schneider-Worthington C.R., Shirazipour C., Buman M.P., de la Haye K., Salvy S.-J. (2022). Integration of Time-Based Recommendations with Current Pediatric Health Behavior Guidelines: Implications for Obesity Prevention and Treatment in Youth. Curr. Obes. Rep..

[17] Wilhite K., Booker B., Huang B.-H., Antczak D., Corbett L., Parker P., Noetel M., Rissel C., Lonsdale C., del Pozo Cruz B. (2023). Combinations of Physical Activity, Sedentary Behavior, and Sleep Duration and Their Associations With Physical, Psychological, and Educational Outcomes in Children and Adolescents: A Systematic Review. Am. J. Epidemiol..

[18] Alanazi Y.A., Sousa-Sá E., Chong K.H., Parrish A.-M., Okely A.D. (2021). Systematic Review of the Relationships between 24-Hour Movement Behaviours and Health Indicators in School-Aged Children from Arab-Speaking Countries. Int. J. Environ. Res. Public Health.

[19] Leech R.M., Chappel S.E., Ridgers N.D., Eicher-Miller H.A., Maddison R., McNaughton S.A. (2024). Analytic Methods for Understanding the Temporal Patterning of Dietary and 24-H Movement Behaviors: A Scoping Review. Adv. Nutr. Int. Rev. J..

[20] Huang J., Li X., Li G., Haegele J.A., Zou L., Chen S., Li C. (2024). Prevalence of Meeting 24-Hour Movement Guidelines and Its Associations with Health Indicators in People with Disabilities: A Systematic Review and Meta-Analysis. Disabil. Health J..

[21] Hartson K.R., Huntington-Moskos L., Sears C.G., Genova G., Mathis C., Ford W., Rhodes R.E. (2023). Use of Electronic Ecological Momentary Assessment Methodologies in Physical Activity, Sedentary Behavior, and Sleep Research in Young Adults: Systematic Review. J. Med. Internet Res..

[22] Patience M., Janssen X., Kirk A., McCrory S., Russell E., Hodgson W., Crawford M. (2023). 24-Hour Movement Behaviours (Physical Activity, Sedentary Behaviour and Sleep) Association with Glycaemic Control and Psychosocial Outcomes in Adolescents with Type 1 Diabetes: A Systematic Review of Quantitative and Qualitative Studies. Int. J. Environ. Res. Public Health.

[23] Bao R., Qin H., Memon A.R., Chen S., López-Gil J.F., Liu S., Zou L., Cai Y. (2024). Is Adherence to the 24-h Movement Guidelines Associated with Greater Academic-Related Outcomes in Children and Adolescents? A Systematic Review and Meta-Analysis. Eur. J. Pediatr..

[24] Chong K.H., Parrish A.-M., Cliff D.P., Kemp B.J., Zhang Z., Okely A.D. (2020). Changes in Physical Activity, Sedentary Behaviour and Sleep across the Transition from Primary to Secondary School: A Systematic Review. J. Sci. Med. Sport.

[25] Maddren C.I., Dhamrait G., Elliott K., Toledo-Vargas M., Gryech I., Okely A.D. (2024). Associations between Postnatal Pollution Exposures, 24-h Movement Behaviours, and Motor Development Outcomes among Children (0-12 Years Old): A Systematic Review. Indoor Air.

[26] Arksey H., O’Malley L. (2005). Scoping Studies: Towards a Methodological Framework. Int. J. Soc. Res. Methodol..

[27] Peters M.D., Marnie C., Tricco A.C., Pollock D., Munn Z., Alexander L., McInerney P., Godfrey C.M., Khalil H. (2020). Updated Methodological Guidance for the Conduct of Scoping Reviews. JBI Evid. Synth..

[28] Julian V., Haschke F., Fearnbach N., Gomahr J., Pixner T., Furthner D., Weghuber D., Thivel D. (2022). Effects of Movement Behaviors on Overall Health and Appetite Control: Current Evidence and Perspectives in Children and Adolescents. Curr. Obes. Rep..

[29] Peters M.D.J., Godfrey C., McInerney P., Khalil H., Larsen P., Marnie C., Pollock D., Tricco A.C., Munn Z. (2022). Best Practice Guidance and Reporting Items for the Development of Scoping Review Protocols. JBI Evid. Synth..

[30] McGowan J., Straus S., Moher D., Langlois E.V., O’Brien K.K., Horsley T., Aldcroft A., Zarin W., Garitty C.M., Hempel S. (2020). Reporting Scoping Reviews—PRISMA ScR Extension. J. Clin. Epidemiol..

[31] da Silva Andressa A.F., Carlos C.A.S.A.-J., Pessini J.P., de Moraes Trindade E.B.S., Silva D.A.S. (2022). Suicidal Behaviors and Sedentary Behavior in Adolescents: Systematic Review and Meta-Analysis. Rev. Andal. De Med. Del Deporte.

[32] World Health Organization: Global Nutrition Monitoring Framework: Operational Guidance for Tracking Progress Towards 2025 Targets. https://scholar.google.com/scholar_lookup?title=Global%20nutrition%20monitoring%20framework%3A%20operational%20guidance%20for%20tracking%20progress%20in%20meeting%20targets%20for%202025&publication_year=2017.

[33] Page M.J., Moher D., Bossuyt P.M., Boutron I., Hoffmann T.C., Mulrow C.D., Shamseer L., Tetzlaff J.M., Akl E.A., Brennan S.E. (2021). PRISMA 2020 Explanation and Elaboration: Updated Guidance and Exemplars for Reporting Systematic Reviews. BMJ.

[34] Ouzzani M., Hammady H., Fedorowicz Z., Elmagarmid A. (2016). Rayyan—A Web and Mobile App for Systematic Reviews. Syst. Rev..

[35] Zhao M., Zhang Y., Herold F., Chen J., Hou M., Zhang Z., Gao Y., Sun J., Hossain M.M., Kramer A.F. (2023). Associations between Meeting 24-Hour Movement Guidelines and Myopia among School-Aged Children: A Cross-Sectional Study. Complement. Ther. Clin. Pract..

[36] Marques A., Ramirez-Campillo R., Gouveia É.R., Ferrari G., Tesler R., Marconcin P., Loureiro V., Peralta M., Sarmento H. (2023). 24-h Movement Guidelines and Overweight and Obesity Indicators in Toddlers, Children and Adolescents: A Systematic Review and Meta-Analysis. Sports Med.-Open.

[37] Neville R.D., Hopkins W.G., McArthur B.A., Draper C.E., Madigan S. (2024). Associations Between Changes in 24-Hour Movement Behaviors in Children and Adolescents During the COVID-19 Pandemic: A Systematic Review and Mediation-Based Meta-Analysis. J. Phys. Act. Health.

[38] Ocvirk T., Kovaÿ M., Jurak G. (2021). Vpliv Omejitev Gibanja Za Obvladovanje Širjenja Virusa SARS-CoV-2 Na 24-Urno Gibalno Vedenje in Telesno Zmogljivost Otrok in Mladostnikov./The Impact of Movement Restriction to Control the Spread of SARS-CoV-2 Virus on 24-Hour Movement Behaviour and Physical Fitness of Children and Adolescents. Revija Šport..

[39] Rodrigues B., Encantado J., Carraça E., Martins J., Marques A., Lopes L., Sousa-Sá E., Cliff D., Mendes R., Santos R. (2023). Questionnaires Measuring 24-Hour Movement Behaviors in Childhood and Adolescence: Content Description and Measurement Properties-A Systematic Review. J. Phys. Act. Health.

[40] Rodrigo-Sanjoaquín J., Bois J.E., Aibar Solana A., Lhuisset L., Corral-Abós A., Zaragoza Casterad J. (2023). Are School-Based Interventions Promoting 24-Hour Movement Guidelines among Children? A Scoping Review. Health Educ. J..

[41] Sampasa-Kanyinga H., Colman I., Goldfield G.S., Janssen I., Wang J., Podinic I., Tremblay M.S., Saunders T.J., Sampson M., Chaput J.-P. (2020). Combinations of Physical Activity, Sedentary Time, and Sleep Duration and Their Associations with Depressive Symptoms and Other Mental Health Problems in Children and Adolescents: A Systematic Review. Int. J. Behav. Nutr. Phys. Act..

[42] Šuc A., Einfalt L., Šarabon N., Kastelic K. (2024). Validity and Reliability of Self-Reported Methods for Assessment of 24-h Movement Behaviours: A Systematic Review. Int. J. Behav. Nutr. Phys. Act..

[43] Wenden E.J., Virgara R., Pearce N., Budgeon C., Christian H.E. (2023). Movement Behavior Policies in the Early Childhood Education and Care Setting: An International Scoping Review. Front. Public Health.

[44] Zhang D., Chen S., Lopez-Gil J.F., Hong J., Wang F., Liu Y. (2023). 24-Hour Movement Behaviours Research during the COVID-19 Pandemic: A Systematic Scoping Review. BMC Public Health.

[45] López-Gil J.F., Tapia-Serrano M.A., Sevil-Serrano J., Sánchez-Miguel P.A., García-Hermoso A. (2023). Are 24-Hour Movement Recommendations Associated with Obesity-Related Indicators in the Young Population? A Meta-Analysis. Obesity.

[46] Arts J., Gubbels J.S., Verhoeff A.P., Chinapaw M.J.M., Lettink A., Altenburg T.M. (2022). A Systematic Review of Proxy-Report Questionnaires Assessing Physical Activity, Sedentary Behavior and/or Sleep in Young Children (Aged 0–5 Years). Int. J. Behav. Nutr. Phys. Act..

[47] Chaput J., Saunders J., Carson V. (2017). Interactions between Sleep, Movement and Other Non-Movement Behaviours in the Pathogenesis of Childhood Obesity. Obes. Rev..

[48] de Mello G.T., Minatto G., Costa R.M., Leech R.M., Cao Y., Lee R.E., Silva K.S. (2024). Clusters of 24-Hour Movement Behavior and Diet and Their Relationship with Health Indicators among Youth: A Systematic Review. BMC Public Health.

[49] Feng J., Huang W.Y., Zheng C., Jiao J., Khan A., Nisar M., Wong S.H.-S. (2024). The Overflow Effects of Movement Behaviour Change Interventions for Children and Adolescents: A Systematic Review and Meta-Analysis of Randomised Controlled Trials. Sports Med..

[50] Fortnum K., Gomersall S.R., Ross M.H., Woodforde J., Thomas G., Wen Y.-S., Perales F., Stylianou M. (2024). 24-Hour Movement Behaviors of LGBTQA+ Young People: A Systematic Review. J. Phys. Act. Health.

[51] Fournier E., Luszczki E., Isacco L., Chanseaume-Bussiere E., Gryson C., Chambrier C., Drapeau V., Chaput J.-P., Thivel D. (2023). Toward an Integrated Consideration of 24 h Movement Guidelines and Nutritional Recommendations. Nutrients.

[52] Grgic J., Dumuid D., Bengoechea E.G., Shrestha N., Bauman A., Olds T., Pedisic Z. (2018). Health Outcomes Associated with Reallocations of Time between Sleep, Sedentary Behaviour, and Physical Activity: A Systematic Scoping Review of Isotemporal Substitution Studies. Int. J. Behav. Nutr. Phys. Act..

[53] Groves C.I., Huong C., Porter C.D., Summerville B., Swafford I., Witham B., Hayward M., Kwan M.Y.W., Brown D.M.Y. (2024). Associations between 24-h Movement Behaviors and Indicators of Mental Health and Well-Being across the Lifespan: A Systematic Review. J. Act. Sedentary Sleep Behav..

[54] Hao Y., Zhou X., Razman R., Peng S., Ahmad N.S. (2024). Compliance with the 24-Hour Movement Behaviour Guidelines among Children and Adolescents with Disabilities: A Systematic Review and Meta-Analysis. BMC Public Health.

[55] Jiang S., Ng J.Y.Y., Chong K.H., Peng B., Ha A.S. (2024). Effects of eHealth Interventions on 24-Hour Movement Behaviors among Preschoolers: Systematic Review and Meta-Analysis. J. Med. Internet Res..

[56] Lee E.-Y., Park S., Kim Y.-B., Lee M., Lim H., Ross-White A., Janssen I., Spence J.C., Tremblay M.S. (2024). Exploring the Interplay Between Climate Change, 24-Hour Movement Behavior, and Health: A Systematic Review. J. Phys. Act. Health.

[57] Kracht C.L., Burkart S., Groves C.I., Balbim G.M., Pfledderer C.D., Porter C.D., St Laurent C.W., Johnson E.K., Brown D.M.Y. (2024). 24-Hour Movement Behavior Adherence and Associations with Health Outcomes: An Umbrella Review. J. Act. Sedentary Sleep Behav..

[58] Ross R., Chaput J.-P., Giangregorio L.M., Janssen I., Saunders T.J., Kho M.E., Poitras V.J., Tomasone J.R., El-Kotob R., McLaughlin E.C. (2020). Canadian 24-Hour Movement Guidelines for Adults Aged 18–64 Years and Adults Aged 65 Years or Older: An Integration of Physical Activity, Sedentary Behaviour, and Sleep. Appl. Physiol. Nutr. Metab..

[59] Hirshkowitz M., Whiton K., Albert S.M., Alessi C., Bruni O., DonCarlos L., Hazen N., Herman J., Katz E.S., Kheirandish-Gozal L. (2015). National Sleep Foundation’s Sleep Time Duration Recommendations: Methodology and Results Summary. Sleep Health.

[60] Caspersen C.J., Powell K.E., Christenson G.M. (1985). Physical Activity, Exercise, and Physical Fitness: Definitions and Distinctions for Health-Related Research. Public Health Rep..

[61] Parrish A.-M., Tremblay M.S., Carson S., Veldman S.L.C., Cliff D., Vella S., Chong K.H., Nacher M., Cruz B.d.P., Ellis Y. (2020). Comparing and Assessing Physical Activity Guidelines for Children and Adolescents: A Systematic Literature Review and Analysis. Int. J. Behav. Nutr. Phys. Act..

[62] World Health Organization (2019). Guidelines on Physical Activity, Sedentary Behaviour and Sleep for Children Under 5 Years of Age.

[63] Bull F.C., Al-Ansari S.S., Biddle S., Borodulin K., Buman M.P., Cardon G., Carty C., Chaput J.-P., Chastin S., Chou R. (2020). World Health Organization 2020 Guidelines on Physical Activity and Sedentary Behaviour. Br. J. Sports Med..

[64] Bronfenbrenner U., Morris P.A., Damon W., Lerner R.M. (2007). The Bioecological Model of Human Development. Handbook of Child Psychology.

[65] Lau P.W.C., Song H., Song D., Wang J.-J., Zhen S., Shi L., Yu R. (2024). 24-Hour Movement Behaviors and Executive Functions in Preschoolers: A Compositional and Isotemporal Reallocation Analysis. Child Dev..

[66] Fung H., Yeo B.T., Chen C., Lo J.C., Chee M.W., Ong J.L. (2023). Adherence to 24-Hour Movement Recommendations and Health Indicators in Early Adolescence: Cross-Sectional and Longitudinal Associations in the Adolescent Brain Cognitive Development Study. J. Adolesc. Health.

[67] Sequí-Domínguez I., Cavero-Redondo I., Álvarez-Bueno C., López-Gil J.F., Martínez-Vizcaíno V., Pascual-Morena C. (2024). Effectiveness of eHealth Interventions Promoting Physical Activity in Children and Adolescents: Systematic Review and Meta-Analysis. J. Med. Internet Res..

[68] Zhou P., Li Y., Lau P.W., Yan L., Song H., Shi T.L. (2024). Effectiveness of Parent-Based Electronic Health (eHealth) Intervention on Physical Activity, Dietary Behaviors, and Sleep in Preschoolers: A Systematic Review. J. Exerc. Sci. Fit..

