# Supplementary material for: Mapping Evidence on Integrated 24-Hour Movement Behaviors in Children and Adolescents: A Scoping Review of Reviews
Source: Children (Basel). 2025 Feb 20;12(3):260. doi: 10.3390/children12030260 (PMC11940917; doi:10.3390/children12030260)
Supplement: Supplementary file 1 [file children-12-00260-s001.zip › Table S1 updated on 09-10-2024.pdf]

Table S1. Search strategy in different databases.

1 - ***PubMed via National Library of Medicine (MEDLINE)*** search performed on: 05/03/2023 + Uptade on: 10/29/2024.		
**Block**	**Descriptors**	**Articles**
1	_"preschool*" OR "child*" OR "adolesc*" OR "young*" OR "youth" OR "student*" OR "teena*"_	***All fields***: 5.610.035 + 491.295<br>***Title/abstract*** 2.761.203 + 295.855
2	_(("movement behavio*" OR "24-h*" OR "guidelines" OR "recommendations" OR "combination") AND ("physical activity" OR "inactivity physical")) AND ("screen" OR "sedentary") AND "sleep*")_	***All fields***: 979 + 400<br>***Title/abstract:*** _905 + 383_
1 + 2	_("preschool*" OR "child*" OR "adolesc*" OR "young*" OR "youth" OR "student*" OR "teena*") AND (("movement behavio*" OR "24-h*" OR "guidelines" OR "recommendations" OR "combination") AND ("physical activity" OR "inactivity physical")) AND ("screen" OR "sedentary") AND "sleep*"))_	***All fields***: 704 + 279<br>***Title/abstract***: 589 + 260
("preschool*" OR "child*" OR "adolesc*" OR "young*" OR "youth" OR "student*" OR "teena*") AND ((("movement behavio*" OR "24-h*" OR "guidelines" OR "recommendations" OR "combination") AND ("physical activity" OR "inactivity physical")) AND ("screen" OR "sedentary") AND "sleep*"))<br><br>_Filters applied: Meta-Analysis, Review, Systematic Review_<br><br>_Filters applied uptade: Meta-Analysis, Review, Systematic Review, Child: 6-12 years, Adolescent: 13-18 years, Adult: 19+ years, Preschool Child: 2-5 years, Young Adult: 19-24 years, Adult: 19-44 years._		67 + 31 = **98**

2 - ***Web of Science*** search performed on: 05/03/2023. Uptade on: 10/29/2024.		
**Block**	**Descriptors**	**Articles**
1	_"preschool*" OR "child*" OR "adolesc*" OR "young*" OR "youth" OR "student*" OR "teena*"_	***All fields:*** 6.745.348 + 816.542<br>**Abstract:** 2.841.634 + 378.256<br>***Title:*** 1.923.275 + 196.363
2	_(("movement behavio*" OR "24-h*" OR "guidelines" OR "recommendations" OR "combination") AND ("physical activity" OR "inactivity physical")) AND ("screen" OR "sedentary") AND ("sleep*")_	***All fields:*** 1.211 + 503<br>**Abstract:** 817 + 334<br>***Title:*** 50 + 10
1 + 2	_("preschool*" OR "child*" OR "adolesc*" OR "young*" OR "youth" OR "student*" OR "teena*") AND (("movement behavio*" OR "24-h*" OR "guidelines" OR "recommendations" OR "combination") AND ("physical activity" OR "inactivity physical")) AND ("screen" OR "sedentary") AND ("sleep*"))_	***All fields***: 852 + 355<br>***Title:*** 27 + 6<br>**Abstract:** 537 + 230
("preschool*" OR "child*" OR "adolesc*" OR "young*" OR "youth" OR "student*" OR "teena*") AND (("movement behavio*" OR "24-h*" OR "guidelines" OR "recommendations" OR "combination") AND ("physical activity" OR "inactivity physical")) AND ("screen" OR "sedentary") AND "sleep*"))<br><br>_Filters Applied: Review_<br>_Filters applied uptade: Review article_		69 + 42 = **111**

3 - ***Scopus*** search performed on: 05/03/2023. Uptade on: 10/29/2024.		
**Block**	**Descriptors**	**Articles**
1	*"preschool*" OR "child*" OR "adolesc*" OR "young*" OR "youth" OR "student*" OR "teena*"*	***All fields***: 17.595.335 + 2.200.740<br>***Title/Abstract/Keywords*** : 7.698.325 + 737.888
2	*(("movement behavio*" OR "24-h*" OR "guidelines" OR "recommendations" OR "combination") AND ("physical activity" OR "inactivity physical")) AND ("screen" OR "sedentary") AND ("sleep*")*	***All fields***: 19.963 + 8.864<br>***Title/Abstract/Keywords*** : 1.271 + 522
1 + 2	*("preschool*" OR "child*" OR "adolesc*" OR "young*" OR "youth" OR "student*" OR "teena*") AND (("movement behavio*" OR "24-h*" OR "guidelines" OR "recommendations" OR "combination") AND ("physical activity" OR "inactivity physical")) AND ("screen" OR "sedentary") AND ("sleep*")*	***All fields***: 17.887 + 7.900<br>***Title/Abstract/Keywords*** : 878 + 363
(ALL ("movement behavio*" OR "24-h*" OR "guidelines" OR "recommendations" OR "combination") AND ALL ("physical activity" OR "inactivity physical") AND ALL ("screen" OR "sedentary") AND ALL ("sleep*") AND ALL ("preschool*" OR "child*" OR "adolesc*" OR "young*" OR "youth" OR "student*" OR "teena*"))<br>*Filters Applied: Review*<br><br>*(TITLE-ABS-KEY ("movement behavio*" OR "24-h*" OR "guidelines" OR "recommendations" OR "combination") AND TITLE-ABS-KEY ("physical activity" OR "inactivity physical") AND TITLE-ABS-KEY ("screen" OR "sedentary") AND TITLE-ABS-KEY ("sleep*") AND TITLE-ABS-KEY ("preschool*" OR "child*" OR "adolesc*" OR "young*" OR "youth" OR "student*" OR "teena*")) AND (LIMIT-TO (DOCTYPE , "re"))*<br><br>*Filters Applied: Review*<br>*Filters applied uptade: Review*		3.350 + 1.421<br><br>77 + 39 = **116**

4 - ***SPORTDiscus via EBSCOhost*** search performed on: 05/08/2023. Uptade on: 10/29/2024.		
**Block**	**Descriptors**	**Articles**
1	*"preschool*" OR "child*" OR "adolesc*" OR "young*" OR "youth" OR "student*" OR "teena*"*	***Full Text***: 329.219 + 1.851.850<br>***Title***: 124.688 + 378.220<br>***Abstract***: 219.889 + 713.620
2	*(("movement behavio*" OR "24-h*" OR "guidelines" OR "recommendations" OR "combination") AND ("physical activity" OR "inactivity physical")) AND ("screen" OR "sedentary") AND "sleep*")*	***Full Text***: 18.013 + 10.956<br>***Title:*** 33 + 23<br>***Abstract***: 808 + 780
1 + 2	*("preschool*" OR "child*" OR "adolesc*" OR "young*" OR "youth" OR "student*" OR "teena*") AND (("movement behavio*" OR "24-h*" OR "guidelines" OR "recommendations" OR "combination") AND ("physical activity" OR "inactivity physical")) AND ("screen" OR "sedentary") AND "sleep*"))*	***Full Text***: 16.578 + 801<br>***Title***: 25 + 3<br>***Abstract***: 114 + 45

AB ("movement behavio*" OR "24-h*" OR "guidelines" OR "recommendations" OR "combination") AND AB ("physical activity" OR "inactivity physical") AND AB ("screen" OR "sedentary") AND AB "sleep*" AND AB ("preschool*" OR "child*" OR "adolesc*" OR "young*" OR "youth" OR "student*" OR "teena*")<br><br>Filters Applied: academic Review<br>No filters were applied in the update due to database unavailability, but the term 'review' was added to the full-text search.	114 + 135 = **249**

5 - ***LILACS via Biblioteca Virtual em Saúde*** search performed on: 05/08/2023. Uptade on: 11/01/2024.		
**Block**	**Descriptors**	**Articles**
1	*"preschool*" OR "child*" OR "adolesc*" OR "young*" OR "youth" OR "student*" OR "teena*"*	***Title***: 17.497 + 771<br>***Abstract***: 43.026 + 3.243<br>**Title, abstract, subject:** 199.023 + 6.509
2	*(("movement behavio*" OR "24-h*" OR "guidelines" OR "recommendations" OR "combination") AND ("physical activity" OR "inactivity physical")) AND ("screen" OR "sedentary") AND "sleep*")*	***Title***: 0 + 0<br>***Abstract***: 9 + 0<br>**Title, abstract, subject:** 12 + 0
1 + 2	*("preschool*" OR "child*" OR "adolesc*" OR "young*" OR "youth" OR "student*" OR "teena*") AND (("movement behavio*" OR "24-h*" OR "guidelines" OR "recommendations" OR "combination") AND ("physical activity" OR "inactivity physical")) AND ("screen" OR "sedentary") AND "sleep*"))*	***Title***: 0 + 771<br>***Abstract***: 1 + 0<br>**Title, abstract, subject:** 7 + 0
Filters Applied: none		7 + 0 = **7**

6 - ***Scientific Eletronic Library Online*** (SciELO*)* search performed on: 05/08/2023. Uptade on: 11/01/2024.		
**Block**	**Descriptors**	**Articles**
1	*"preschool*" OR "child*" OR "adolesc*" OR "young*" OR "youth" OR "student*" OR "teena*"*	***Title***: 595 + 202<br>***Abstract***: 22.163 + 4.827<br>**All indexes:** 1.944 + 648
2	*(("movement behavio*" OR "24-h*" OR "guidelines" OR "recommendations" OR "combination") AND ("physical activity" OR "inactivity physical")) AND ("screen" OR "sedentary") AND "sleep*")*	***Title***: 0 + 0<br>***Abstract***: 9 + 0<br>**All indexes:** 0 + 0
1 + 2	*("preschool*" OR "child*" OR "adolesc*" OR "young*" OR "youth" OR "student*" OR "teena*") AND (("movement behavio*" OR "24-h*" OR "guidelines" OR "recommendations" OR "combination") AND ("physical activity" OR "inactivity physical")) AND ("screen" OR "sedentary") AND "sleep*"))*	***Title***: 0 + 0<br>***Abstract***: 3 + 0<br>**All indexes:** 0 + 0
(ab:("preschool*" OR "child*" OR "adolesc*" OR "young*" OR "youth" OR "student*" OR "teena*")) AND (ab:("movement behavio*" OR "24-h*" OR "guidelines" OR "recommendations" OR "combination")) AND (ab:("physical activity" OR "inactivity physical")) AND (ab:("screen" OR "sedentary")) AND (ab:("sleep*"))		3 + 0 = **3**

7 - ***PsycINFO via American Psychological Association (APA)*** search performed on: 05/08/2023. Uptade on: 11/01/2024.		
**Block**	**Descriptors**	**Articles**

1	*"preschool*" OR "child*" OR "adolesc*" OR "young*" OR "youth" OR "student*" OR "teena*"*	***Any fields***: 2.358.074 + 133.241<br>***Title:*** *763,713 + 24.936*<br>***Abstract:*** 1.482.157 + 93.155
2	*(("movement behavio*" OR "24-h*" OR "guidelines" OR "recommendations" OR "combination") AND ("physical activity" OR "inactivity physical")) AND ("screen" OR "sedentary") AND "sleep*")*	***Any fields***: 393 + 117<br>***Title***: 8 + 1<br>***Abstract:*** 285 + 100
1 + 2	*("preschool*" OR "child*" OR "adolesc*" OR "young*" OR "youth" OR "student*" OR "teena*") AND (("movement behavio*" OR "24-h*" OR "guidelines" OR "recommendations" OR "combination") AND ("physical activity" OR "inactivity physical")) AND ("screen" OR "sedentary") AND "sleep*"))*	***Any fields***: 316 + 81<br>***Title:*** 7 + 1<br>***Abstract:*** 204 + 4
"preschool" OR Any Field: "child*" OR Any Field: "adolesc*" OR Any Field: "young*" OR Any Field: "youth" OR Any Field: "student*" OR Any Field: "teena*" AND Any Field: "movement behavio*" OR Any Field: "24-h*" OR Any Field: "guidelines" OR Any Field: "recommendations" OR Any Field: "combination" AND Any Field: "physical activity" OR Any Field: "inactivity physical" AND Any Field: "screen" OR Any Field: "sedentary" AND Any Field: "sleep*"*<br><br>*Filters Applied: methology – literature Review, systematic review*		24 + 4 = **28**

8 - ***Cumulative Index to Nursing and Allied Health Literature (CINAHL), via EBSCOhost***. Busca realizada em: 05/08/2023. Uptade on: 10/29/2024.		
**Block**	**Descriptors**	**Articles**
1	*"preschool*" OR "child*" OR "adolesc*" OR "young*" OR "youth" OR "student*" OR "teena*"*	***Full Texts***: 6.397.320 + 155.394<br>***Title*:** 1.274.386 + 55.060<br>***Abstract:*** 2.119.169 + 51.933
2	*("movement behavio*" OR "24-h*" OR "guidelines" OR "recommendations" OR "combination") AND ("physical activity" OR "inactivity physical")) AND ("screen" OR "sedentary") AND "sleep*")*	***Full Texts***: 18.013 + 125<br>***Title***: 48 + 4<br>***Abstract:*** 808 + 98
1 + 2	*("preschool*" OR "child*" OR "adolesc*" OR "young*" OR "youth" OR "student*" OR "teena*") AND (("movement behavio*" OR "24-h*" OR "guidelines" OR "recommendations" OR "combination") AND ("physical activity" OR "inactivity physical")) AND ("screen" OR "sedentary") AND "sleep*"))*	***Full Texts***: 16.578 + 101<br>***Title:*** 33 + 3<br>***Abstract:*** 546 + 70
AB ("movement behavio" OR "24-h*" OR "guidelines" OR "recommendations" OR "combination") AND AB ("physical activity" OR "inactivity physical") AND AB ("screen" OR "sedentary") AND AB "sleep*" AND AB ("preschool*" OR "child*" OR "adolesc*" OR "young*" OR "youth" OR "student*" OR "teena*")*<br><br>*Filters Applied: academic Review, age – all child (6-12 years), adolescente (13-18 years), child, preschool (2 – 5 years), all infant*<br><br>*AB ("movement behavio*" OR "24-h*" OR "guidelines" OR "recommendations" OR "combination") AND AB ("physical activity" OR "inactivity physical") AND AB ("screen" OR "sedentary") AND AB "sleep*" AND AB ("preschool*" OR "child*" OR "adolesc*" OR "young*" OR "youth" OR "student*" OR "teena*") AND review*		211 + 8 = **219**
