# Supplementary material for: Mapping Evidence on Integrated 24-Hour Movement Behaviors in Children and Adolescents: A Scoping Review of Reviews
Source: Children (Basel). 2025 Feb 20;12(3):260. doi: 10.3390/children12030260 (PMC11940917; doi:10.3390/children12030260)
Supplement: Supplementary file 1 [file children-12-00260-s001.zip › Table S2 Articles removed according to the exclusion criteria 12-02-2025.pdf]

Table S2. Articles removed according to the exclusion criteria.

Author(s) and year	Title	Reason for exclusion
Ahmed et al. (2021)	Global Prevalence of Physical Activity, Sedentary Behaviour, and Sleep of Immigrant Children: a Systematic Review.	Did not evaluate the 24-Hour Movement Behaviors in an integrated way.
Brown et al. (2022)	A scoping review of outcomes commonly reported in obesity prevention interventions aiming to improve obesity-related health behaviors in children to age 5 years.	Did not evaluate the 24-Hour Movement Behaviors in an integrated way.
Byrne et al. (2019)	Brief tools to measure obesity-related behaviours in children under 5 years of age: A systematic review.	Did not include the age group of the study.
Clevenger & Montoye (2023)	Systematic Review of Accelerometer Responsiveness to Change for Measuring Physical Activity, Sedentary Behavior, or Sleep	Did not evaluate the 24-Hour Movement Behaviors in an integrated way.
Ferguson et al. (2021)	Annual, seasonal, cultural and vacation patterns in sleep, sedentary behaviour and physical activity: a systematic review and meta-analysis.	Did not include the age group of the study.
Giurgiu et al. (2022a)	The Assessment of 24-Hr Physical Behavior in Children and Adolescents via Wearables: A Systematic Review of Laboratory Validation Studies.	Did not evaluate the 24-Hour Movement Behaviors in an integrated way.
Giurgiu et al. (2022b)	Assessment of 24-hour physical behaviour in children and adolescents via wearables: a systematic review of free-living validation studies.	Did not evaluate the 24-Hour Movement Behaviors in an integrated way.
Gomahr et al. (2022)	Childhood obesity prevention: what can be achieved?	It was not the objective to summarize evidence about the 24-Hour Movement.
Huang et al. (2021)	Relationships of physical activity and sedentary behaviour with the previous and subsequent nights' sleep in children and youth: A systematic review and meta-analysis.	Did not evaluate the 24-Hour Movement Behaviors in an integrated way.
Huang et al. 2023	To examine the associations of social jetlag or chronotype with physical activity and sedentary behaviour among children and adolescents aged between 3 and 17 years.	
Jensen et al. (2013)	Associations between sleep, dietary intake and physical activity in children: a systematic review.	Did not evaluate the 24-Hour Movement Behaviors in an integrated way.
Katzmarzyk et al. (2019)	International Study of Childhood Obesity, Lifestyle and the Environment (ISCOLE): Contributions to Understanding the Global Obesity Epidemic.	It was not the objective to summarize evidence about the 24-Hour Movement.
Kharel et al. (2022)	Impact of COVID-19 pandemic lockdown on movement behaviours of children and adolescents: a systematic review.	Did not evaluate the 24-Hour Movement Behaviors in an integrated way.
Kim et al. (2021)	Ambient air pollution and movement behaviours: A scoping review.	Did not specify the age range of children and adolescents.

Kim et al. (2017)	The accuracy of the 24-h activity recall method for assessing sedentary behaviour: the physical activity measurement survey (PAMS) project.	It is not a review study.
Lua et al. (2023)	A Narrative Review of Screen Time and Wellbeing among Adolescents before and during the COVID-19 Pandemic: Implications for the Future.	Did not evaluate the 24-Hour Movement Behaviors in an integrated way.
Ng & Ng (2022)	Impact of the COVID-19 pandemic on children's mental health: A systematic review	Did not evaluate the 24-Hour Movement Behaviors in an integrated way.
Parrish et al. (2020)	Comparing and assessing physical activity guidelines for children and adolescents: a systematic literature review and analysis.	Did not refer to 24-Hour Movement Behavior.
Paterson et al. (2021)	Exploring the impact of COVID-19 on the movement behaviors of children and youth: A scoping review of evidence after the first year.	Did not evaluate the 24-Hour Movement Behaviors in an integrated way.
Rico-Gonzalez et al. (2024)	Preschool children's adherence to the 24-hour physical activity guidelines: A systematic review comparing the sexes	It was not the objective to summarize evidence about the 24-Hour Movement Behaviour
Weaver et al. (2022)	Seasonal Shifts in Children's Sedentary Behaviors, Physical Activity, and Sleep: A Systematic Review and meta-Analysis.	Did not refer to 24-Hour Movement Behavior.
Wang & Zang (2022)	International Experience and Enlightenment of Physical Activity Guidelines for Children and Adolescents.	It is not a review study.
Zisis et al. (2021)	Climate change, 24-hour movement behaviors, and health: a mini umbrella review.	Did not specify the age range of children and adolescents.
