# Supplementary material for: Mapping Evidence on Integrated 24-Hour Movement Behaviors in Children and Adolescents: A Scoping Review of Reviews
Source: Children (Basel). 2025 Feb 20;12(3):260. doi: 10.3390/children12030260 (PMC11940917; doi:10.3390/children12030260)
Supplement: Supplementary file 1 [file children-12-00260-s001.zip › Table S3 Characteristics of included studies by dimension group 12-02-2025.pdf]

Table S3. Characteristics of included studies by aims research (*n* = 42).

Author/ Year	Country/ Continent	Title	Type of review	Quantity and names of Databases	Date of searches and period of coverage	Search keywords and combinations	Population / Sample and age range of the sample
**Research aims: 24-HOUR MOVEMENT GUIDELINES**							
Jurakiÿ & Pedišiÿ 2019 [11]	Croatia – Europe	Croatian 24-hour guidelines for physical activity, sedentary behavior, and sleep: A proposal based on a systematic review of literature.	Systematic	Four databases: PubMed/ MEDLINE, Scopus, SPORTDiscus and Web of Science (including Science Citation Index Expanded, Social Sciences Citation Index and Arts & Humanities Citation Index). A search was also carried out using the same keywords using the Google search engine.	May 2019 – Articles and documents, without publishing time constraints.	("physical activity" OR sedentary OR sleep OR movement OR "24-hour*" OR "24 hour*") AND (guidelines OR recommendations)	Children and adolescents Croatians from zero to 17 years old.
Rodrigo-Sanjoaquín et al. 2023 [40]	France – Europe	Are school-based interventions promoting 24-hour movement guidelines among children? A scoping review.	Scoping	Five databases: Pubmed, Scopus, SPORTDiscuss, The Cochrane Library and Web of Science.	Between January 2010 and December 2020 – intervention with a control group measure.	Child* OR student OR pupil OR infant OR childhood OR school* OR schoolchildren OR school children OR schoolage* OR school-age* OR school age* OR primary school OR elementary school OR basic school NOT adolescent* Strategy* OR technique* OR intervention* OR program* OR health prevention OR health prevention program* OR health program* OR health promotion program* OR health promotion intervention OR health education OR health intervention OR school setting OR school based intervention OR school-based intervention OR school based program* OR school-based program* OR school program* OR school health program OR school intervention OR school health intervention (PA OR Total PA OR MVPA OR VPA OR LPA OR total physical activity OR moderate to vigorous physical activity OR vigorous	Childhood and adolescence from five to 12 years old.

						physical activity OR light physical activity OR physical activity OR physical inactivity OR active OR activit* OR sport* OR sports participation OR active transport OR active commuting OR leisure activity OR walking OR aerobic exercise OR outdoor play OR exercis* OR motor behavior * OR movement) AND (sedentary * OR sedentary behavior * OR sedentary time OR sedentary lifestyle OR total sedentary time OR sitting behavior * OR sitting time OR prolonged sitting OR domestic activities OR computer use OR computer time OR media use OR video games OR tablet use OR smartphone use OR mobile phone use OR computer time OR television time OR TV time OR gaming OR screen time OR screen-time OR reading OR TV viewing OR TV child room OR television viewing OR video viewing) AND (sleep* OR sleep behavior * OR sleep duration OR sleep quality OR sleep pattern* OR bedtime OR sleep disturbance OR insomnia)	
Vidmar et al. 2022 [16]	United States of America – North America	Integration of Time-Based Recommendations with Current Pediatric Health Behavior Guidelines: Implications for Obesity Prevention and Treatment in Youth.	Narrative - Literature	-	-	-	Children and adolescents from zero to 18 years old.
Wenden et al. 2023 [43]	Australia – Oceania	Movement behavior policies in the early childhood education and care setting: An international scoping review.	Scoping	Seven databases: EMBASE, CINAHL, Web of Science, Proquest, Scopus, EBSCO and PubMed. A Google search was also performed using the same keyword selections and limited to the first 200 results.	Between July 1, 2021 and October 2021.	A set of keywords he was developed using the Web *of* Science citation search tool to check and adapt Keyword selections and combinations. ALL= (physical activity AND sedentary AND sleep OR screen*) AND ALL= (early years OR preschool OR child* OR kinder*) AND ALL= (policy OR guideline OR recommend* OR	Children from zero to 6 years of age.

						statement OR strategy) AND DOP =2010-01- 01 – 2021-10-31<br><br>Google - "Early Childhood Education and Care"; (Kindergarten or Preschool or Child*), (Physical* or "Screen time" or Sedentary or Sleep), Obesity, (Guideline or Statement or Policy or Recommendation or Strategy).	
**Research aims: MEASUREMENT OF 24-HOUR MOVEMENT**							
Arts et al. 2022 [46]	Holland – Europe	A systematic review of proxy-report questionnaires assessing physical activity, sedentary behavior and/or sleep in young children (aged 0–5 years).	Systematic	Three databases: PubMed, Embase and SPORTDiscus.	January 2020, first search – For physical activity and sedentary behavior questionnaires from December 2015, while for sleep questionnaires there was no lower limit for publication date. Update, of combined behaviors, was completed on January 6, 2021.	Search strategies focused in terms related to children small (for example, infant, toddler, preschooler), proxy reporting measures (e.g., example, questionnaire, proxy-report) and measurement properties (e.g., example, reliability, reproducibility, validity). For activity search physics and behavior sedentary, these terms they were used in combination with terms related to the activity physics (for example, motor activity, exercise) OR behavior sedentary (for example, stationary behavior, screen-time). For the search for sleep, these terms they were used in AND combination with terms related to the sleep (for example, bedtime, nap). Complete search strategies are presented in an additional file in the manuscript.	Children aged zero to five years.
Hartson et al. 2023 [21]	United States of America – North America	Use of Electronic Ecological Momentary Assessment Methodologies in Physical Activity, Sedentary Behavior, and Sleep Research in Young Adults: Systematic Review.	Systematic	Five databases: PubMed, CINAHL, PsycINFO, Embase, and Web of Science.	Conducted in August 2021 – and follow-up search using the same procedures in August 2022.	Terms and synonyms: "physical activity," "sedentary behavior," "sleep," "ecological momentary assessment," and "young adults."	18-25 years; mean age 18-23 years and age range 17-29 years; or a sample escribed as "young adults" or

							“college students” if no age range or mean age was provided.
Leech et al. 2024 [19]	Australia – Oceania	Analytic Methods for Understanding the Temporal Patterning of Dietary and 24-H Movement Behaviors: A Scoping Review.	Scoping	Three databases: Embase, ProQuest, and EBSCOhost (PsycINFO, MEDLINE Complete, SportDiscus).	Was conducted on 3 May, 2021. Identify studies published between 1997 and an updated literature search using the same databases, search, and screening strategies was conducted in July 2022.	Keywords used for the literature search covered four main concepts, including dietary and movement behaviors, behavior context, temporal patterning, and novel analytic methods	Did not specify age.
Lettink et al. 2022 [15]	Netherlands – Europe	Systematic review of accelerometer-based methods for 24-h physical behavior assessment in young children (0–5 years old).	Systematic	One database: PubMed (MEDLINE).	June 2021 – Reliability and validity articles published until June 26, 2021.	Terms related to children small (for example, infant, toddler, preschooler), methods joint in accelerometer (for example, accelerometry/methods, actigraphy) and properties measurement (for example, validity, reliability). These terms they were used in combination with terms related to the behavior physical *(AND)*: behavior sedentary (for example, inactive behavior, stationary behavior, sitting), activity physics (for example, motor activity, tummy time, cycling), *AND* sleep (for e.g., nap, bedtime, night rest). Articles related the animals, a variety of disorders (for example, autism, attention deficit disorder) and diseases they were excluded using the combination *NOT*. † Larger details about your terms used for searching they can to see verified directly at source review.	Children from zero to five years.
Rodrigues et al. 2023 [39]	Portugal – Europe	Questionnaires Measuring 24-Hour Movement Behaviors in Childhood and Adolescence: Content	Scoping	Four databases: PubMed, PsycINFO, SPORTDiscus and EMBASE.	June 2021 – no search time limits.	They were used variations of the terms, “children”, “adolescents”, “sleep”, “sedentary behavior”, “physical activity”, “movement behaviors”, “questionnaire” and	Children from five to 12 years old.

		Description and Measurement Properties- A Systematic Review.				"measurement properties".	
Suc et at., 2024 [42]	Slovenia – Europe	Validity and reliability of self-reported methods for assessment of 24-h movement behaviours: a systematic review.	Systematic	Three databases: PubMed, Scopus, and SPORTDiscus.	Was performed in May 2022, and updated in September 2023.	The primary search query combined terms: movement behaviours, self reported method, and validity/reliability. Complementary information's on Supplementary Table.	Adolescents (aged 12 to 17 years), adults (aged 18 to 64 years), or older adults (aged 65 years and older).

Research aims: ADHERENCE TO 24-HOUR MOVEMENT GUIDELINES

Fortinum et al. 2024 [50]	Australia – Oceania	24-Hour Movement Behaviors of LGBTQA+ Young People: A Systematic Review	Systematic Review.	Seven databases: Cochrane, EMBASE, PsycINFO, PubMed, Scopus, SPORTDiscus, and Web of Science.	From inception to January 2024.	The search strategy was initially developed based on terms related to the targeted population and outcomes of interest. Example: Cochrane (lgb* OR "sexual and gender minority" OR "sexual and gender minorities" OR lesbian* OR gay OR gays OR homosexual* OR bisexual* OR transsex* OR transgender* OR queer* OR intersex* OR "gender dysphoria" OR "gender minority" OR "gender minorities" OR "gender identity" OR "gender identities" OR "gender fluid" OR "gender fluidity" OR "gender nonconforming" OR "gender nonconformity" OR "sexual minority" OR "sexual minorities" OR "sexual orientation" OR "sexual orientations" OR "sexual identity" OR "sexual identities" OR "non heterosexual" OR "non heterosexuals"):ti,ab,kw OR MeSH descriptor: [Sexual and Gender Minorities] explode all trees OR MeSH descriptor: [Gender Identity] explode all trees AND	Participants ≤24 years of age or a mean age ≤ 24 years. Data synthesis: Children and adolescents (ie, aged < 18 years). Young adults (ie, aged 18 – 24 years).

							(Exercis* OR "physical activity" OR “physical activities” OR "resistance training" OR "strength training" OR "endurance training" OR "aerobic training" OR "cardiovascular training" OR sport* OR athlet* OR sedentar* OR "physical inactivity" OR "sitting time" OR "standing time" OR "screen time" OR sleep* OR Insomnia* OR bedtime OR "bed time"):ti,ab,kw OR MeSH descriptor: [Exercise] explode all trees OR MeSH descriptor: [Sports] explode all trees OR MeSH descriptor: [Sleep] explode all trees	
Hao et al. 2024 [54]	China Asia	–	Compliance with the 24-hour movement behaviour guidelines among children and adolescents with disabilities: a systematic review and meta-analysis.	Systematics and meta-analysis	Seven databases: Web of Science, PubMed (NIBI), SPORTDiscus (EBSCO), CINAHL (EBSCO), MEDLINE (EBSCO), Scopus and Psychology and Behavioural Sciences Collection (EBSCO).	Up to May 2023.	1) 24-hour movement behaviour: ‘movement behavior*’ OR ‘24-h*’ OR ‘physical activit*’ OR screen* OR sleep* 2) Children and adolescents: child* OR adolescen* OR youth OR teenager* OR student* OR school OR ‘school*aged’ OR ‘5–17 years’ OR juvenile 3) Disabilities: disab* OR ‘physical disabilit*’ OR ‘developmental disabilit*’ OR ‘developmental delay’ OR ‘learning disabilit*’ OR ‘intellectual disabilit*’ OR ‘mental* retard*’ OR ‘down syndrome’ OR DS OR ‘autism spectrum disorder*’ OR ‘attention deficit hyperactivity disorder*’ OR ADHD OR ‘cerebral palsy’ OR ‘tourette syndrome’ OR ‘neurocognitive disorder*’ OR ‘neurocognitive disabilit*’ OR ‘cognitive impairment*’ OR ‘cognitive dis*’ ‘acquired brain injur*’ OR ‘motor skills disorder*’ OR ‘developmental coordination disorder*’ OR ‘sensory impairment*’ OR ‘emotional impairment*’ OR	Between 5 and 17 years, or with a mean/ median sample age between 5 and 17 years.

						deafness OR blindness OR ‘language disorder*’ OR special<br>4) S1 AND S2 AND S3	
Huang et al. 2024 [14]	China – Asia	24-H movement behaviours research in Chinese population: A scoping review	Scoping	Five databases:<br>Web of Science, PubMed, Scopus, EBSCOhost, and CNKI (Chinese database).	Up to October 2023.	Two main keywords: “24-h movement behaviours” and “Chinese population”.	No specified range age – Chinese population.
Tapia-Serrano et al. 2022 [1]	Spain – Europe	Prevalence of meeting 24-Hour Prevalence of meeting 24-Hour Movement Guidelines from pre-school to adolescence: A systematic review and meta-analysis including 387,437 participants and 23 countries.	Systematics and meta-analysis	Four databases: PubMed, Scopus, Web of Science and Cochrane Database of Systematic Reviews.	From June 16, 2016 to May 20, 2021.	All combinations possible of the following groups of search terms: (a) “early*” OR “preschool*” OR “child*” OR “adolesc*” OR “young*” OR “youth” OR “student*” OR “teena*”; (b) “movement behavior*” OR “24-h*”; (c) “physical activity” OR “screen” OR “sleep*”; (d) “guidelines” OR “recommendations”.<br>You search terms they were adapted for each database in combination with filters specific to the database, as search strategy presented at revision.	Preschoolers to adolescents from 23 countries, among three to 18 years old.
Zhang et al. 2023 [44]	China – Asia	24-Hour movement behaviours research during the COVID-19 pandemic: a systematic scoping review.	Scoping	Three databases: Web of Science, EBSCO, and PubMed	From 1 to 2020 to 30 November 2022.	Several keywords were employed for the literature search in each database: “24-h*”, “24 hour”, “24-hour”, “Movement Behavio*”, “Sleep*”, “Screen”, “Physical Activity”, “Guideline*”, “recommendation*”, “COVID-19” “Coronavirus Disease”, “Coronavirus”, “SARS-CoV-2” and “nCoV”.<br>In the Web of Science, EBSCO, and PubMed, we divided all search terms into three categories: (24-h* OR 24 h OR 24-hour OR Movement Behavio* OR Sleep* OR Screen OR Physical Activity) AND (Guideline* OR recommendation*) AND (COVID-19 OR Coronavirus Disease OR Coronavirus OR SARS-CoV-2 OR nCoV).	People who had COVID-19.

**Research aims: CHANGES IN TIME SPENT IN 24-HOUR MOVEMENT BEHAVIORS**							
Chong et al. 2020 [24]	Australia – Oceania	Changes in physical activity, sedentary behaviour and sleep across the transition from primary to secondary school: A systematic review.	Systematic	Six databases: PsycINFO, PubMed, Scopus, SPORTDiscus, Web of Science and China Academic Journal Network Publishing.	May 2019 – and limited to original articles published from January 1990 to May 31, 2019.	child* OR adolescen* OR youth AND “primary school” OR “elementary school” OR “secondary school” OR “middle school” OR “high school” AND longitudinal OR follow-up OR cohort OR tracking OR transition AND “physical activity*” OR sport OR exercise OR sedentary* OR “sedentary behavior*” OR computer* OR “TV viewing” OR “video gam*” OR “electronic gam*” OR internet OR tablet OR smartphone OR “social media” OR television OR screen-time OR screen-based OR “screen based” OR “non-screen-based” OR sitting OR sleeping* Search strategies they were provided in appendix in the manuscript.	From elementary school (two last years) for high school (two first years) from 10 to 13 years old.
Feng et al. 2024 [49]	China – Asia	The Overflow Effects of Movement Behaviour Change Interventions for Children and Adolescents: A Systematic Review and MetaAnalysis of Randomised Controlled Trials.	Systematic and metaa nalysis.	Six databases: MEDLINE (Ovid), PsycINFO (ProQuest), EMBASE (Ovid), PubMed, Web of Science and SPORTDiscus (EBSCO).	On 7 March, 2022 and updated on 13 May, 2024.	Details of the search strategy are available in Table S1 of the Electronic Supplementary Material.	Aged under 18 years.
Miatke et al. 2024 [13]	Australia – Oceania	The association between reallocations of time and health using compositional data analysis: a systematic scoping review with an interactive data exploration interface.	Scoping	Four databases: MEDLINE (through Ovid), Embase (through Ovid), Scopus, and SPORTDiscus (through EBSCOhost).	Papers published since 2015. And run on the 13th of October 2022.	The search syntax for the MEDLINE platform is outlined is provided in Additional file.	No specified range age - All populations were considered, including clinical populations.
Neville et al. 2024 [37]	Ireland – Europe	Associations Between Changes in 24-Hour Movement Behaviors	Systematic Review and Mediation-Based	Six databases: PubMed, MEDLINE, Embase, PsycINFO, SPORTDiscus, and Web of Science.	Between January 1, 2020 and June 27, 2022.	The broad search concepts included (1) physical activity, sedentary behavior, screen time, and sleep; (2) children or adolescents; and (3) COVID-19.	Children and adolescents, ≥5 to ≤18 y.

		in Children and Adolescents During the COVID-19 Pandemic: A Systematic Review and Mediation-Based Meta-Analysis.	Meta-Analysis.			The full search strategy, including keywords used for each search concept in Embase, is displayed in Supplementary Table.	
Ocvirk, Kovaÿ & Jurak 2021 [38]	Switzerland – Europe	The impact of movement restriction to control the spread of SARS-CoV-2 virus on 24-hour movement behaviour and physical fitness of children and adolescents.	Narratives – Literature	A database ‡: Pubmed. In addition to Google Scholar and UNICEF.	February 2021 – Articles (did not identify the search period)	It was used one combination of following words in English for the research line: "COVID-19, physical activity, sedentary behavior, sleep, children, adolescents, physical performance".	Children and adolescents. No specified range age.

Research aims: HEALTH AND 24-HOUR MOVEMENT

Alanazi et al. 2021 [18]	Australia – Oceania	Systematic review of the relationships between 24-hour movement behaviours and health indicators in school-aged children from arab-speaking countries.	Systematic	Six databases to identify articles in English and French: MEDLINE, EMBASE, SPORTdiscus, CINAHL, PsycINFO and Scopus. Eight bases for identifying articles in Arabic: Saudi Digital Library, ArabBase, Human Index, KSUP, Pan-Arab Academic Journal, e- Marefa and Al Manhal eLibrary. In addition to Google Scholar‡.	January 2021 limited to original articles published between January 1990 and January 2021.	"Physical activity*" OR "Movement behavior*" OR "physical inactivity" OR exercise OR "physical fitness" OR "energy expenditure" OR "sedentary*" OR sit* OR sitting OR lifestyle OR "television view*" OR "tv view*" OR "screen time" OR "electronic media" OR Sleep* OR Bedtime OR "Bed time" OR Nap* OR "Time on bed" OR "Night rest" Child* Arab* OR "Saudi Arabia" OR "United Arab Emirates" OR Bahrain OR Kuwait OR Oman OR Qatar OR Egypt OR Sudan OR Palestine OR Jordan OR Iraq OR Lebanon OR Syria OR Yemen OR Libya OR Morocco OR Tunisia OR Algeria OR Comoros OR Djibouti OR Mauritania OR Somalia.	Children in school age of speaking countries Arabic, apparently healthy, overweight / or obesity, between five and 12 years old, or age average between five and 12 years.
Chaput et al. 2017 [47]	Canada – North America	Interactions between sleep, movement and other non-movement behaviours in the pathogenesis of childhood obesity.	Narrative	-	-	-	Children and adolescents, aged six to 17.
De Melo et al. 2024 [48]	Brazil – South America	Clusters of 24-hour movement behavior and diet and their relationship with health	Systematic	Five databases: PubMed, Scopus, Web of Science, LILACS, and PsycINFO.	Papers published up to and May 2018. And updated at the begin of April 2023.	The search encompassed sets of descriptors associated with behaviors (e.g., diet), person-oriented statistical	Children and/or adolescents aged 19 years

		indicators among youth: a systematic review.				approaches (e.g., 'cluster analysis'), and specifc populations (e.g., adolescents*).	and younger or mean age between this range.
Fournier et al. 2023 [51]	France – Europe	Toward an Integrated Consideration of 24 h Movement Guidelines and Nutritional Recommendations.	Narrative	Three databases: PubMed/MEDLINE, ScienceDirect and MEDLINE. The websites of scientific organizations, such as the WHO, were also searched.	From November 2022 to February 2023.	The principal search terms used were: 24 h movement guidelines, movement behavior, physical activity, sleep, sedentary behaviors, sedentary time, diet, eating habits, dietary guidelines, dietary recommendations, adult, youth, children, and adolescent.	Children and adolescents from three to 18 years old, and adults from 18 to 65 years.
Grgic et al. 2018 [52]	Australia – Oceania	Health outcomes associated with reallocations of time between sleep, sedentary behaviour, and physical activity: a systematic scoping review of isotemporal substitution studies.	Scoping	Eight databases:Academic Search Premier, CINAHL, Health Source: Nursing/Academic Edition, MasterFILE Premier, PsycINFO, PubMed/MEDLINE, Scopus, SPORTDiscus , and Web of Science (including Arts & Humanities Citation Index, Conference Proceedings Citation Index- Science, Conference Proceedings Citation Index- Social Science & Humanities, Science Citation Index Expanded, and Social Sciences Citation Index).	July 2017 – Articles originals. No limitations were applied to publication date.	"physical activity", "physical inactivity", sedentary*, sleep*, sitting, standing, isotemporal, compositional.	Children and adolescents and populations clinics. No specified range age.
Groves et al. 2024 [53]	United States of America – North America	Associations between 24-h movement behaviors and indicators of mental health and well-being across the lifespan: a systematic review.	Systematic	Four databases: MEDLINE, PsycINFO, Embase, and SPORTDiscus.	July 2022 and subsequently updated in February 2023 and August 2023.	Search terms can be found in Additional file of the study.	Not specified. Across the lifespan – Samples of children and youth, ranging from 3 to 17 years of age, and for adults, ranging from 15 to 79 years of age.
Huang et al. 2024 [20]	China – Asia	Prevalence of meeting 24-hour movement guidelines and its associations with health	Systematic and meta-analysis	Six databases: PubMed, PsycINFO, Scopus, Web of Science (core collection), SPORTDiscus, and Embase.	Up to May 31, 2023.	Groups of search terms: (a) disability OR disabilities OR disabled OR impairment OR impaired OR "special needs"; (b) "physical activit*" OR	People with disabilities – Did not specify age.

		indicators in people with disabilities: A systematic review and meta-analysis.				sport* OR exercise OR "active play"; (c) sedentary OR sitting OR inactive OR "screen time" OR screentime; (d) sleep; and (e) ("movement behavio*" OR 24-h*) AND (guideline* OR recommendation*).	
Julian et al. 2022 [28]	France – Europe	Effects of Movement Behaviors on Overall Health and Appetite Control: Current Evidence and Perspectives in Children and Adolescents.	Narrative	-	-	-	Children and adolescents from all over the world. No specified range age.
Kracht et al. 2024 [57]	United States of America – North America	24-hour movement behavior adherence and associations with health outcomes: an umbrella review.	Umbrella review	Eight databases: CINAHL, Medline (EBSCO), PsychINFO, SportDiscus, Scopus, Web of Science, Cochrane Library, and Embase.	The first six databases were searched from inception to October 12th, 2023. Then Cochrane Library and eighth (Embase) were then searched from inception to October 24th, 2023 and October 31st, 2023.	The full search strategies are presented in Appendix.	Without age constraints.
Lannoy et al. 2023 [8]	Canada – North America	Evidence supporting a combined movement behavior approach for children and youth's mental health – A scoping review and environmental scan.	Scoping	Four databases: PsycINFO, MEDLINE, CINAHL and Scopus.	Between June 17, 2022 and July 28, 2022.	The research concepts included: "physical activity", "sleep", "sedentary behavior", "mental health", "children" and "youth". Search terms additional together with the research concepts included you following mental health indicators (well -being and mental illness): emotional health, emotion regulation, executive functioning, mindfulness, psychological adjustment, psychological functioning, optimism, psychosocial, health-related quality of life (HRQoL), resilience, satisfaction with life (or life satisfaction), self-compassion, self-efficacy, self-esteem, self-regulation, socio-emotional, wellbeing, attention-deficit/hyperactivity	Children and adolescents aged zero to 17.

						disorder (ADHD), attention deficit disorder (ADD), autism spectrum disorder (ASD), anxiety, bipolar disorder, conduct disorder, depression (or depressive symptoms), disruptive behavior disorder, distress, eating disorder (or pathology or disordered eating), emotion dysregulation, externalizing behavior, impulsivity, inattention, internalizing behavior, hyperactivity, learning disorders, mood disorder, oppositional defiant disorder and stress.<br>† Due to the complexity of the search strategy, greater details they are presented directly at source review.	
Lee et al. 2024 [56]	Canada – North America	Exploring the Interplay Between Climate Change, 24-Hour Movement Behavior, and Health: A Systematic Review.	Systematic	Seven databases: Ovid MEDLINE, Ovid PsycINFO, EbscoHost SPORTDiscus, Sports Medicine & Education Index, EbscoHost CINAHL, Engineering Village GeoRef, and Ovid EMBASE.	Published in 2000 and onward to only capture the evidence published in the past 25 years. The initial searches were conducted in March 2020 and top-up searches were conducted on April 13, 2023 and April 18, 2024.<br><br>A hand search by the primary investigator (Lee) was also conducted on November 5, 2020, July 30, 2023, and on May 10, 2024 to ensure that the most up-to-date, relevant studies post top-up searches were	Detailed information about search records and strategies are presented in Supplementary Tables.	Did not specify age.

					included in the review.		
Lópes-Gil et al. 2023 [45]	Spain – Europe	Are associated with obesity-related indicators in the young population? A meta-analysis.	Systematic and meta-analysis	Four databases: PubMed, Scopus, Web of Science, and Cochrane Database of Systematic Reviews.	From June 16, 2016, to October 26, 2022.	The research strategy was based on PECOS (Population, Exposure, Comparator, Outcome, and Study design), using the following BMI, “body mass index”, overweight, obes*, “weight status” OR “body composition”, “body fat”, fatness, adiposity, “weight circumference”, “trunk fat mas”, “waist to height ratio”; “movement behavio*”, “24-hour*”; preschool*, early*, child*, young*, adolesc*, student*, youth, teena*; recommendations, guidelines; screen, “physical activity”, sleep*. The specific search terms for each of the previously mentioned databases, in combination with specific filters, are shown in Supporting Information.	Young people aged three to 18 years.
Marques et al. 2023 [36]	Portugal – Europe	24-h Movement Guidelines and Overweight and Obesity Indicators in Toddlers, Children and Adolescents: A Systematic Review and Meta-Analysis.	Systematic and meta-analysis.	Three databases: PubMed, Web of Science and Scopus.	Articles published until 31 December 2022.	Included combinations of: (obes* OR weight* OR overweight OR “body composition” OR fat* OR adiposity OR BMI OR body OR fitness*) AND (“24-Hour Movement” OR “Canadian guideline*” OR “physical activity guideline*” OR “physical activity recommendation*” OR “sedentary behaviour guideline*” OR “sedentary behavior guideline*” OR “sedentary behaviour recommendation*” OR “sedentary behavior recommendation*” OR “sleep guideline*” OR “sleep recommendation*” OR “screen time guideline*” OR “screen time recommendation*”).	Toddlers (1–4 years), children (5–12 years) and adolescents (13–17 years).
Patience et al. 2023 23 [22]	Scotland - Europe	24-Hour Movement Behaviours (Physical Activity, Sedentary Behaviour and Sleep) Association with Glycaemic Control and Psychosocial Outcomes	Systematic	Eight databases: MEDLINE (Ovid), EMBASE (Ovid), Web of Science (Core Collection), APAPsychINFO (EBSCOhost), SPORTDiscus (EBSCOhost), Applied Social Sciences Index and Abstracts (ProQuest),	May 7, 2021- Articles quantitative (experimental or not) published until the search date.	1. Type 1 Diabetes<br>2. Adolescent<br>3. Physical Activity<br>4. Sedentary Behaviour<br>5. Sleep<br>6. 24-hour Movement Behaviour	Adolescents diagnosed with type 1 diabetes aged 11 to 18.

		in Adolescents with Type 1 Diabetes: A Systematic Review of Quantitative and Qualitative Studies.		Sports Medicine and Education Index (ProQuest) Wiley Cochrane Library, OpenGrey and Open Dissertations (EBSCOhost).		7. Health Behaviour or Health Knowledge or Health Attitude or Health Practice (Broader Variety of Phrases to Comprehensively Incorporate both Qualitative and Quantitative Studies)<br>8. HbA1c, CGM Metrics and Quality of Life<br>9. Depressive Symptoms, Anxiety, Stress, Self-Management, Coping, Self-Efficacy, Family Functioning and Social Competence<br>10. 1 and 2 (Population/Sample)<br>11.3 or 4 or 5 or 6 or 7 (Intervention/Exposure/Phenomenon of Interest)<br>12.8 or 9 (Primary and Secondary Outcomes)<br>13.10 and 11 and 12 (Results)<br>For each database, it was developed one search strategy composed per keywords and synonyms, using the PICO and SPIDER frameworks.<br>† Bigger details about the search strategy used in each base can be checked directly at revision.	
Rollo, Antsygina & Tremblay, 2020 [7]	Canada – North America	The whole day matters: Understanding 24-hour movement guideline adherence and relationships with health indicators across the lifespan.	Systematic	Five databases: PsycINFO, PubMed, SPORTDiscus, Web of Science, and Ovid MEDLINE.	Between January 2015 and January 2020 – Studies without design limits.	† The review no presented this information, below is presented the idea search initial published in Prospero.<br>The search strategy was built for PubMed, and the search terms were adapted for use with other databases, in combination with database-specific filters, when available, according to topics:<br>Condition or domain in study - Correlations, adherence and health associations with 24- hour movement behaviors (activity physics, behavior sedentary and sleep) to throughout life.<br>Participants / population - Lifelong; without population restrictions and/or age. Early years (0-4 years); children	All tracks' ages, babies (aged < 1 year) children toddlers (aged 1 to 2 years old) preschoolers (aged 3 to 4 years old) children (aged 5 to 11 years old) young people (aged 12 to 17 years old) adults (aged 18 to 64 years old) and

						and young people (5-17 years old); adults (18-64 years old); elderly (65+ years).<br>Intervention (s), exhibition (s) - Adhesion (meeting vs. no meeting) at 24-hour movement guidelines (activity physics, sleep and behavior sedentary) or composition of 24-hour use movement behavior (activity physics, sleep and behavior sedentary).<br>Comparator (s)/ control - No to meet to the guidelines or to meet just partially to the guidelines.	elderly (≥ 65 years old).
Sampasa-Kanyinga et al. 2020 [41]	Canada – North America	Combinations of physical activity, sedentary time, and sleep duration and their associations with depressive symptoms and other mental health problems in children and adolescents: A systematic review.	Systematic	Four databases: MEDLINE, EMBASE, PsycINFO and SPORTDiscus.	September 30, 2019.<br>September 30, 2019.<br>September 29, 2019, using the Ovid interface.<br>SPORTDiscus - from the beginning until September 29, 2019, using the EBSCO platform.	† The review no presented these information below they are presented the idea search structure initial according to PICOS.<br>Population - children and adolescents Apparently healthy in school age (aged between 5 and 17 years).<br>Intervention - the three movement behaviors (or that is, activity physical activity, sedentary time and sleep duration).<br>Comparator - No he was necessary.<br>Type of study – No there was restriction.	Children and adolescents Apparently healthy in school age from five to 17 years old.
Saunders et al. 2016 [3]	Canada – North America	Combinations of physical activity, sedentary behaviour and sleep: Relationships with health indicators in school-aged children and youth.	Systematic	Five databases: MEDLINE, EMBASE, SPORTdiscus, CINAHL and PsycINFO.	A new search he was carried out using the CINAHL database (EbscoHost) on June 2, 2015, focused in the combinations of 2 or more movement behaviors, without date limit, language and study design.	For each database it was carried out search distinct, for therefore, search strategies complete they are available directly in the Archive Supplementary article.	Children and adolescents aged five to 17.99 years.
Wilhite et al. 2023 [17]	Australia – Oceania	Combinations of Physical Activity, Sedentary Behavior, and Sleep Duration and Their Associations With	Systematic	Seven databases: MEDLINE (National Library of Medicine, Bethesda, Maryland), Cumulative Index to Nursing and Allied Health	June 2020 (no publication date setting).	Any hair result any less two of the movement behaviors combined (activity physics, behavior sedentary status and sleep duration) with some	Children and adolescents, aged 5 to 17.

		Physical, Psychological, and Educational Outcomes in Children and Adolescents: A Systematic Review.		Literature (CINAHL) (EBSCO Industries, Birmingham, Alabama), PsychInfo (American Psychological Association, Washington, DC), SPORTDiscus (EBSCO Industries), PubMed (National Library of Medicine), Excerpta Medica Database (EMBASE) (Elsevier BV, Amsterdam, the Netherlands), and Education Resources Information Center (ERIC) (EBSCO Industries).		result in health physical, psychological and/or educational.	
Zhao et al. 2024 [35]	China – Asia	Association between meeting 24-h movement guidelines and health in children and adolescents aged 5–17 years: a systematic review and meta-analysis.	Systematic and meta-analysis.	Six databases: MEDLINE, EMBASE, PubMed, Web of Science, CINAHL, and SPORTDiscus.	Data restricted to the period from 2016 to 2022.	Using the following search strategy: ((24 h*) OR (movement behavio*) OR (movement guidelin*) OR (physical activit* AND sedentary behavio* AND slee*)). The research terms were customized for each research database.	Aged 5–18 years children (aged 5–12 years) and adolescent (aged 13–18 years).

Research aims: OTHER 24-HOUR MOVEMENT ASSOCIATIONS

Bao et al. 2024 [23]	Callaghan – Australia	Is adherence to the 24-h movement guidelines associated with greater academic-related outcomes in children and adolescents? A systematic review and meta-analysis.	Systematic and Meta-analysis.	Six databases: PubMed, PsycINFO, Scopus, WOS, SPORTDiscus, and EMBASE.	December 12, 2023.	Two themes: 24-h movement guidelines and academic-related outcomes.	Mean age ranged from 9.17 to 15.7 years.
Jiang et al. 2024 [55]	China – Asia	Effects of eHealth Interventions on 24-Hour Movement Behaviors Among Preschoolers: Systematic Review and Meta-Analysis.	Systematic and meta-analysis.	Six databases: PubMed, Ovid, SPORTDiscus, Scopus, Web of Science, and Cochrane Central Register of Controlled Trials.	Inception to February 08, 2023.	Used the search terms: "eHealth," "Physical activity," "Sedentary behavior," "Sleep," "preschooler," and their Medical Subject Headings terms.	Preschool children aged 2 to 6 years.
Maddren et al. 2024 [25]	Australia – Oceania	Associations Between Postnatal Pollution Exposures, 24-h Movement Behaviours, and Motor Development	Systematic	Eigth databases: CINAHL, Embase, ERIC, Global Health, MEDLINE, PsycINFO, Scopus, and Web of Science - Core Collection.	From their inception to May 2023.	A search strategy was developed that included keywords related to pollution, 24-h movement behaviours, and motor development, with a final	Zero to 12 years old.

Outcomes Among Children (0–12 Years Old): A Systematic Review		search strategy for each database implemented.

Note: *Used to define prefixes in searches, † Authors' note. ‡ Google Scholar was considered gray literature even when the review itself classified it as a database.
