# Supplementary material for: Mapping Evidence on Integrated 24-Hour Movement Behaviors in Children and Adolescents: A Scoping Review of Reviews
Source: Children (Basel). 2025 Feb 20;12(3):260. doi: 10.3390/children12030260 (PMC11940917; doi:10.3390/children12030260)
Supplement: Supplementary file 1 [file children-12-00260-s001.zip › Table S4 Results of included studies by dimension group 12-02-2025.pdf]

Table S4. Results of included studies by aims research (*n* = 42).

Author/ Year	Purpose of the review	Number of original studies/documents included in the review and total studies related to the 24-Hour Movement in children and/or adolescents	Main evidence of the review in relation to the 24-Hour Movement	Conclusion of the review
**Research aims: 24-HOUR MOVEMENT GUIDELINES**				
Jurakiÿ & Pedišiÿ, 2019 [11]	Conduct a systematic literature review to identify and analyze available 24-hour guidelines for physical activity, sedentary behavior and sleep, and propose Croatian 24-hour guidelines in accordance with existing findings.	Documents included in the review: eight.<br><br>Studies that evaluated 24-hour Movement in children and/or adolescents: five.	The review presents existing guidelines for children and adolescents (including those who exhibit the three behaviors). They identified the guideline on, Canadian, South African (three to five years) and New Zealand Guidelines for children and adolescents (ages zero to 17). Of the total documents, one included children from zero to five years old, one from three to five years old and three children from five to 17 years old.<br>Almost all existing 24-hour recommendations for children and young people aged zero to 17 are based on Canadian recommendations, developed in accordance with currently available scientific evidence. When drafting the Croatian recommendations, the same model was adopted. World Health Organization recommendations and the specificities of the Croatian context were also considered. The Croatian recommendations were developed for the following age groups: children (0-4 years), children and young people (5-17 years), adults (18-64 years) and elderly people (over 65 years).	Given the fact that the recommendations are based on the best scientific evidence currently available, these were used as a basis for creating proposed recommendations<br>24-hour Croatians for children and young people in the present study. When drafting the recommendations, they were adapted to Croatian specificities.<br>Therefore, it is believed that the proposed Croatian 24-hour recommendations for physical activity, sedentary behavior and sleep habits carried out in this research can help promote a "healthier" time distribution for this population. Furthermore, the proposed Croatian 24-hour recommendations can be used to provide individual advice in general practice clinics, medical clinics and recreational centers, as well as help formulate messages in public health campaigns and develop national, local and institutional health policies.
Rodrigo-Sanjoaquín et al. 2023 [40]	To review and summarize the implementation and effectiveness of school-based interventions targeting	Original studies included in the review: 37	The three movement behaviors were measured in a study, with two months of intervention. This study reported no significant effects of the intervention on any of the three behaviors, but did not perform retesting for sleep.	This is the first scoping review of intervention studies focused on the school environment and 24-hour movement behaviors among children

	24-hour movement behaviors among children aged five to 12 years.	Studies that evaluated 24-hour Movement in children and/or adolescents: none.	Furthermore, the study did not analyze movement behaviors in combination.<br><br>There are few interventions with schoolchildren that include sleep and at least one of the other two movement behaviors simultaneously, physical activity and/or sedentary behavior.	ages five to 12. The main finding was the lack of implementation of school-based interventions targeting 24-hour movement behaviors. Furthermore, there is a lack of interventions in primary schools that include sleep in their intervention programs. More research and more studies are needed to explore this gap in the literature and the effectiveness of 24-hour movement behaviors in the school environment.
Vidmar et al. 2022 [16]	Inform a unifying framework of health behaviors and guide future research on integrating time-based recommendations into current quantity- and quality-based health guidelines for children and adolescents.	Original studies included in the review: not reported.<br><br>Studies that evaluated 24-hour Movement in children and/or adolescents: did not specify.	Sleep and movement/activity guidelines (physical activity and sedentary behavior)<br><br>**Middle Childhood (five to 12 years)**<br>*Movement Guidelines*<br>"Until recently, sleep and movement recommendations for children were conceptualized and formulated relatively independently of each other. (..) physical activity recommendations have focused mainly on activity intensity (moderate to vigorous physical activity, which corresponds to 5% of the day's activities) and duration (minutes per day), while sleep guidelines emphasize sleep duration and, to a lesser extent, sleep quality (...) the majority of a child's day is allocated to sleep (~40%), sedentary (~40%), and light physical activity (~15%)".<br><br>**Adolescence and Emerging Adulthood (12 to 18 years old)**<br>*Movement Guidelines*<br>Although clinical guidelines for physical activity in adolescents share similar themes, the recommendations are delivered as independent entities, with no suggestions for how food, sleep, and activity should coexist within a 24-hour period or interact to contribute to health outcomes health over time.<br><br>"Adolescents and young adults may respond more favorably to action-based recommendations (i.e., sitting less implies moving more) than to guidelines that restrict	Although childhood guidelines do not explicitly conceptualize a 24-hour integrated approach, the recommended time spent on these behaviors adds up to 24 hours a day. In recent decades, a number of conceptual frameworks and theoretically driven interventions have emerged to guide research focusing on the optimal timing of behavior and the composition of health behaviors in childhood and adolescence. Recommendations based on diet quality and quantity, sleep duration (in the absence of guidance on timing) and duration and intensity of physical activity are often difficult to implement in caregivers' daily lives. The addition of routine and schedule components could provide a simplified approach to conceptualizing and communicating how health behaviors are integrated throughout the day. This guidance can be especially beneficial for structuring after school hours, weekend days, winter vacations, and summer vacations, when a lack of organized routines and structured schedules can challenge adherence to health-promoting behaviors. Integrating time-

			frequently preferred activities (e.g., screen time).” Additionally, focusing on the importance of the “whole day” rather than independent behaviors also offers the opportunity to examine how small changes in movement (i.e., stopping sedentary behavior) can benefit cardiometabolic and mental health in adolescence.	based recommendations into current health guidelines for children and adolescents will require well-designed longitudinal studies and controlled trials to address high-priority research questions.
Wenden et al. 2023 [43]	Identify movement behavior policies specific to the early childhood education and care sector, from zero to six years old (prevalence, content, development and implementation of policies).	Studies and documents in review: 43<br><br>Studies that evaluated 24-hour Movement in children and/or adolescents: did not specify.	Studies included: 4<br>Documents included: 39<br>Many policies have not been revised since the publication of the 24-Hour Movement Guidelines.<br>The current 24-Hour Movement Guidelines for early years can provide impetus for meeting policy recommendations.	Movement behavior policies in the early childhood health education setting are often vaguely written, without a comprehensive evidence base, developmentally isolated and often not adapted for the 'real world'. Furthermore, movement behavior policies specific to early childhood education and care need to be proportionately aligned with the national/international 24-hour Early Years Movement Behavior Guidelines.
**Research aims: MEASUREMENT OF 24-HOUR MOVEMENT**				
Arts et al. 2022 [46]	Summarize the measurement properties of questionnaires that assess physical activity, sedentary behavior and/or sleep in children aged 0 to 5 years.	Original studies included in the review: 33 (37 questionnaires)<br><br>Studies that evaluated 24-hour Movement in children and/or adolescents: six.	Four questionnaires assessed constructs of all 24-hour movement behaviors for preschoolers (3-5 years of age), two in toddlers and preschoolers. These six questionnaires mostly used very few items per movement behavior, which questions the scope of the items (for example, a single item to assess sedentary behavior).	None of the questionnaires was considered sufficiently valid and/or reliable to assess one or more movement behaviors in children aged zero to five years. The lack of high-quality methodological studies evaluating all relevant measurement properties of the developed questionnaires hampers the ability to draw definitive conclusions about the best available questionnaires. Questionnaires to assess 24-hour movement behaviors in children aged zero to five years are scarce. Therefore, high-quality studies are needed with the objective of developing *proxy-report questionnaires* for this age group and evaluating their measurement properties, based on content validity.

Hartson et al. 2023 [21]	To describe the use of the electronic ecological momentary assessments methodology in physical activity, sedentary behavior, and sleep research among young adults.	Original studies included in the review: 37<br><br>Studies that evaluated 24-hour Movement in children and/or adolescents: none.	None studies included electronic ecological momentary assessment methodologies measures of physical activity, sedentary behavior, and sleep.	Recent technological advances have made electronic ecological momentary assessments an increasingly useful methodology for collecting data regarding movement behaviors and their correlates, particularly among young adults. As movement behavior research shifts toward acknowledging the interconnectedness of behaviors within the 24-hour period, electronic ecological momentary assessments research in this area with young adults is needed.
Leech et al. 2024 [19]	To explore analytic methods for understanding the temporal patterning of multiple dietary and 24-h movement behaviors. Additionally, we explored methods that have incorporated contextual factors in the examination of temporal behavior patterns.	Original studies included in the review: 14<br><br>Studies that evaluated 24-hour Movement in children and/or adolescents: Two	Physical activity, sedentary and sleep assessment tools included accelerometers ActiGraph.<br>Line graphs were the most common data visualization method used to present temporal patterns.<br>The contextual factors examined in the studies of 24-h movement behaviors varied and broadly included factors relating to the built, natural, socioeconomic environment, school hours, activity interests, parental modeling or co-participation, mode of transport to school, log diaries, and survey tools.	Future research examining existing microlongitudinal data or using study designs with daily assessments of behavioral and contextual factors may help elucidate the health implications of different temporal patterns and their determinants. However, this may require advancing data collection through use of wearables, smartphone, and geographic information system technology and the use of existing data on weather, green spaces and parks, food environments, and local infrastructure. Finally, this review identified only 1 study on the temporal patterning of dietary and 24-h movement behaviors, suggesting further research is needed to develop methods for analyzing dietary behaviors together with 24-h movement behaviors across the intensity spectrum.
Lettink et al. 2022 [15]	Comprehensively review all studies examining the test-retest measurement properties, interdevice reliability, criterion, and convergent validity of accelerometer-based methods assessing 24-hour	Original studies included in the review: 62<br><br>Studies that evaluated 24-hour Movement in children and/or adolescents: did not specify	In preschool children (3 to 5 years), valid hip and wrist cutoff points were identified to assess sedentary behavior, light physical activity, moderate to vigorous physical activity, and wrist cutoff points for sleep.<br>Given the lack of reliable and/or valid accelerometer-based methods and the lack of studies on 24-hour	For preschoolers, cutoff points and valid algorithms were identified for all movement behaviors. Multi-parameter methods appeared to have better measurement properties. In general, it is recommended that more high-quality

	movement behaviors in children ages 0 to 5 years, including an assessment of quality of evidence.		movement behaviors, future studies should develop and evaluate methods aimed at including all 24-hour movement behaviors, as well as exploring different sensor and axis positionings using raw acceleration data from modern accelerometers.	studies be carried out that evaluate accelerometer data over 24 hours, in different sensor positions and axes to evaluate movement behaviors. Standardized protocols focusing on the inclusion of well-defined movement behaviors in different contexts representative of the child's developmental stage are needed. Large heterogeneity and methodological limitations preclude definitive conclusions about the best available accelerometer-based methods that assess all 24-hour movement behaviors combined in young children.
Rodrigues et al. 2023 [39]	To systematically review the literature on measurement properties of self-report and proxy questionnaires that measure 24-hour movement behaviors in children and adolescents	Original studies included in the review: 29<br><br>Studies that evaluated 24-hour Movement in children and/or adolescents: none	In the 29 studies, 37 questionnaires were identified.<br>Two questionnaires assessed physical activity, sedentary behavior and sleep, one proxy and the other self-reported. However, none were designed to assess movement behaviors considering the 24-hour movement behavior paradigm.	Existing questionnaires have insufficient measurement properties, and none have considered the 24-hour movement behavior paradigm. These results highlight the need for new and better movement behavior combination questionnaires to improve 24-hour movement behavior monitoring and surveillance systems in this population.
Suc et al., 2024 [42]	To identify validated self-reported tools for assessment of movement behaviours across the whole 24-h day, and to review their atributes (movement behaviours being assessed including temporal and contextual information, accounting for a 24-h day, recall period, number of questions) and quantitative measurement properties (construct validity, testretest reliability, responsiveness).	Original studies included in the review: 16<br><br>Studies that evaluated 24-hour Movement in children and/or adolescents: none	No studies have found self-reported addressed a combination of all 24-hour movement behaviors in adolescents.	There is a need to develop new tools for assessment of 24-h movement behaviours for specific purposes and/or to adapt the existing physical activity and sedentary behavior self-reports in a way that they will resonate with the emerging 24-h movement paradigm. Future studies should examine measurement properties of 24-h movement behaviours estimates simultaneously and by using statistical methods that respect compositional nature of movement behaviours data.
**Research aims: ADHERENCE TO 24-HOUR MOVEMENT GUIDELINES**				

Fortinum et al. 2024 [50]	To synthesize data pertaining to the 24-hour movement behaviors (ie, physical activity, sedentary behavior, sleep) of LGBTQA+ young people (aged < 24 years) and compare these with population-level estimates of meeting the 24-hour movement guidelines.	Original studies included in the review: 56<br><br>Studies that evaluated 24-hour Movement in children and/or adolescents: zero.	There was not found results specifically for the combination of the three movement behavior in children and/or adolescents.	The 24-movement behaviors of LGBTQA+ young people may therefore be contributing to the health disparities seen between LGBTQA+ and non-LGBTQA+ young people. However, the limited number of studies for some outcomes and methodological constraints evident within the available literature contribute to difficulties in synthesizing results and contextualizing the 24-hour movement behaviors—especially sedentary (screen) time, and sleep—of LGBTQA+ young people.
Hao et al. 2024 [54]	To synthesize the existing literature on adherence to the 24-hour movement behaviour among children and adolescents with disabilities, bridging a research gap and offering evidence-based insights for fostering optimal health and well-being in this underserved population.	Original studies included in the systematic review and meta-analysis: 15<br><br>Studies that evaluated 24-hour Movement in children and/or adolescents: 15	16% of children and adolescents with disabilities did not meet any of the 24-hour movement behaviour.<br>The overall compliance with the 24-hour movement behaviour of children and adolescents with disabilities was 7%. There are significant differences in adherence to individual movement guidelines, more children and adolescents with disabilities meet recommendations for sleep (59%) than for physical activity (22%) and sedentary behavior (49%).	The children and adolescents with disabilities were less likely to meet the recommended guidelines for physical activity, screen time, and sleep duration. Furthermore, the majority of them did not fulfil the requirements of all three components outlined in the 24-hour movement behaviour. These findings strongly emphasize the urgent need to tailor the 24-hour movement behaviour for children and adolescents with disabilities. Future research should prioritize the development of integrative the 24-hour movement behaviour specifically designed for the unique needs of this population.
Huang et al. 2024 [14]	To map the evidence on 24-h movement behaviours in Chinese population.	Original studies included in the review: 53<br><br>Studies that evaluated 24-hour Movement in children and/or adolescents: 34	Regarding sample age groups, 32 studies involved children and adolescents on the generally healthy population, and two studies each targeted children with autism spectrum disorder and children and adolescents with intellectual disability. Ten studies targeted preschool children.<br>Specifically, the prevalence of adherence to 24-h movement guidelines varied from 2.9 % to 16.4 % in preschool children (n = 6), 0.3 %–26.1 % in children and adolescents (n = 25), 16.1% in children with autism	This review reveals an increasing trend in the number of articles published on 24-h movement behaviours in recent years, with a predominant focus on children and adolescents, self-report measure, cross-sectional design, guidelines adherence, and health outcomes.

			spectrum disorder (n = 1) and 17.5% chinese children and adolescents with intellectual disabilities (n= 1).<br><br>The design of two were longitudinal and 32 were cross-sectional. Self-report questionnaires were the predominant measure (n=34).	
Tapia-Serrano et al. 2022 [1]	Examining general (non)adherence to the 24-Hour Movement Guidelines among preschoolers, children and adolescents around the world.	Original studies included in the systematic review and meta-analysis: 63<br><br>Studies that evaluated 24-hour Movement in children and/or adolescents: 37	Of the studies analyzed, 30 had a cross-sectional design, five were longitudinal and two were cohort studies. Of these, 13 studies were carried out with children (76,928 aged 6 to 12 years), 17 with adolescents (298,741 aged 13 to 18 years) and seven with children and adolescents.<br>The overall 24-Hour Movement Guidelines meeting stratified by sex was reported in 27 studies, nine of which were studies with children, 11 with adolescents, and seven with children and adolescents.<br>*Measuring behaviors:*<br>**Physical activity** – in 14 studies using accelerometer, in 25 self-reported and in one through parental report, with three studies using an accelerometer and self-reported questionnaire.<br>**Sedentary behavior** – in 35 studies it was self-reported and in two through parental report.<br>**Sleep** – in 9 studies using an accelerometer, in 29 self-reported studies and in two through parental reports, with three studies using an accelerometer and a self-reported questionnaire.<br>*The overall grip to the 24-hour Movement Guidelines* was 10.31% in children and 2.68% in adolescents, that is, children had significantly greater adherence than adolescents.<br>Depending on the region, global adherence, including children aged three to five, as well as children and adolescents aged six to 18, was 17.20% for Africa, 3.80% for Asia, 9.62% for Europe, 7.88% for North America, 10.87% for Oceania and 2.93% for South America. Furthermore, the random effects meta-regression model showed that general adherence to the 24-Hour Movement was positively associated with the country's Human Development Index.	The majority of young people in the 23 countries surveyed did not comply with all three components of the 24-Hour Movement Guidelines. The fact that about one in five children and adolescents do not meet any of the recommendations for healthy movement behaviors is a public health concern. Although interventions aimed at improving adherence to the 24-Hour Movement Guidelines are necessary for all young people, older children, adolescents and girls (especially those from countries with a medium Human Development Index) should be prioritized. Thus, these findings emphasize the critical need to integrate sex- and age-specific strategies to support youth populations in developing and maintaining healthy movement behaviors over time. Special attention should be paid to the South American region due to its low adherence to the 24-Hour Movement Guidelines.<br>In line with sustainable development goals, a new global action plan is needed to not only increase the proportion of young people meeting physical activity recommendations, but also the Screen Time and Sleep Duration Guidelines.

			General adherence to the Movement Guidelines was significantly lower in female children (6.89%) than in male children (11.05%) (p<0.001), but among adolescents no statistically significant differences were reported significant between sexes (girls 6.92 and boys 8.61%, p = 0.110).<br>*Failure to comply with any of the three Guidelines 24-Hour Movement Rate* was significantly lower among children (15.57%) than among adolescents (28.59%) (p<0.001). Clear evidence of publication bias was found (LFK index = 4.52).<br>By region, global adherence to not complying with any of the three 24-Hour Movement Guidelines, including children aged three to five, as well as children and adolescents aged six to 18, was 9.99% in Africa, 25. 77% in Asia, 13.48% in Europe, 17.70% in North America, 11.06% in Oceania, and 31.72% in South America. The pooled prevalence was slightly higher in girls than in boys, although there were no significant differences between the sexes, in female children it was 12.20%, while for males it was 13.91%. In relation to female adolescents it was 14.79% and male 10.16%). The random-effects meta-regression model also showed that adherence to any of the three 24-Hour Movement Guidelines was negatively associated with the country's Human Development Index.	
Zhang et al. 2023 [44]	To synthesize the evidence concerning research using the 24HMG during the COVID-19 pandemic.	Studies and documents in review: 16<br><br>Studies that evaluated 24-hour Movement in children and/or adolescents: five	The five studies with children and/or adolescents showed the prevalence of meeting the 24HMG was under 5% during the COVID-19 pandemic, of which two studies reported a percentage less than 1%.<br>One study reported that during the pandemic, the lowest prevalence of meeting the 24HMG was 0.0%.<br>Four studies reported that the percentage of the population meeting the 24HMG was decreased, of which three of the studies reported a significant decrease from prior to and during the COVID-19 pandemic.<br>Two studies showed a decrease of more than 3%. One study reported a percentage of 0.0% with no change. Nonsignificant increases were reported both in children and youth (only girls).	The COVID-19 may tend to have a negative impact on the prevalence of meeting 24HMG among different age-group populations. According to the study characteristics and research domains, studies using the 24HMG have a large space for improvement in terms of study design, measurement protocols and study domains (e.g., correlates and health outcomes).

Research aims: CHANGES IN TIME SPENT IN 24-HOUR MOVEMENT BEHAVIORS

Chong et al. 2020 [24]	The purpose of this study was to systematically identify and review studies that report changes in time spent in physical activity, sedentary behavior, and sleep, individually and collectively, across the transition from primary to secondary school, to promote further understanding of changes in children's 24h movement behaviors during this critical development period.	Original studies included in the review: six.<br><br>Studies that evaluated 24-hour Movement in children and/or adolescents: none.	No studies were identified that assessed changes in the time use composition of the three movement behaviors across the transition from primary to secondary school, to promote further understanding of changes in children's 24h movement behaviors during this critical development period. Existing studies have primarily focused on components of physical activity and/or sedentary behavior, but outcomes for sleep or whether the 24-hour composition of all three movement behaviors changes during this critical developmental period remain unknown.	Additional high-quality studies using an integrated approach are needed to explore changes in children's general movement behavior patterns and associated factors that accompany the school transition. This information would facilitate the development of effective interventions by identifying and prioritizing risk behavior(s) to address during this critical developmental period and subsequently promote the importance of practicing an optimal pattern of movement behavior 24 hours for the general health and well-being of children and adolescents.
Feng et al. 2024 [49]	To examined the overflow effects of interventions targeting a single movement behaviour on other non-targeted movement behaviours in children and adolescents aged under 18 years. The secondary purpose was to investigate whether the overflow effects vary across different characteristics of the intervention (e.g. age group, setting, duration of intervention).	Original studies included in the review: 102<br>Studies included in meta-analysis: 60<br><br>Studies that evaluated 24-hour Movement in children and/or adolescents: five.	The studies which assessment the three-movement behavior reported:<br>Physical activity interventions in sedentary behavior showed no significant differences between the intervention and control group (two studies).<br>Physical activity interventions in sleep time showed no significant differences between both groups (two studies).<br>Screen time interventions in physical activity showed mixed results. Two studies found no difference at post-intervention, and one reported increased physical activity in the intervention group compared with the control group.<br>Regarding the effects of screen time interventions on sleep, no significant changes were reported in any of the three studies.	Overflow effects on non-targeted behaviours were observed in interventions aimed at increasing physical activity or reducing sedentary behaviour although the effect size was small. These findings shed light on the importance of holistic consideration of a 24-h framework for evaluating movement behaviours.
Miatke et al. 2024 [13]	To provide an overview of studies that have used CoDA to model how reallocating time between different	Original studies included in the review: 103	Across different outcomes and populations, reallocations of time between sleep, sedentary behavior, light physical activity and movement vigorous physical activity were	Reallocating time towards movement vigorous physical activity from any behaviour(s) is generally associated

	time-use components is associated with health.<br>The three specific objectives of the scoping review were to: (1) review and summarise findings from such studies; (2) describe their study designs, samples, health outcomes, time-use behaviours and types of reallocations investigated; and (3) identify research gaps.	Studies that evaluated 24-hour Movement in children and/or adolescents: 31	similarly associated with health outcomes. Was relatively consistent across age groups that reallocating time towards moderate vigorous physical activity was favourably associated with most health outcomes, but it appeared to be weaker in young children than in other age groups.<br>Reallocating time to movement vigorous physical activity was generally favourable and reallocating time away from movement vigorous physical activity was generally unfavourable for health, no matter which behaviour was substituted or being used as a substitute.<br>Health associations for reallocations involving sleep, sedentary behavior and light physical activity (but not movement vigorous physical activity) were inconsistent, and they varied depending on the health outcome. However, even in instances where results were relatively consistent (e.g., favourable association of reallocating sedentary behavior to light physical activity for adiposity), the magnitude of the associations were generally much smaller than for reallocations involving movement vigorous physical activity.<br>Reallocations that did not report confidence intervals or significance levels were generally consistent with those that did, both regarding the magnitude and direction of associations.<br>The finding that reallocating time towards movement vigorous physical activity was favourably associated with most health outcomes was relatively consistent across age groups, but it appeared to be weaker in young children than in other age groups.	with the strongest benefits to health, while reallocating time away from movement vigorous physical activity to any behaviour(s) is associated with the strongest detriment to health. Some beneficial associations were seen when reallocating time from sedentary behavior to both light physical activity and sleep; however, the strength of the association was much lower than for any reallocations involving movement vigorous physical activity. Some evidence suggested that researchers should consider information on where, how and when activity occurs when investigating reallocations of time in future studies.<br>Future studies may benefit from investigating associations with other health outcomes (e.g., cognitive, social and developmental outcomes), which may be particularly relevant to other populations. More research is also needed using longitudinal data, which will give insight into how people actually reallocate their time under different conditions in real life. To determine the health effects of real (as opposed to hypothetical) reallocations of time, more isotemporal substitution studies should be conducted using data from intervention trials.
Neville et al. 2024 [37]	To estimate whether changes in one domain of child and adolescent movement behaviors from before to during the COVID-19 pandemic were directionally associated with change in another domain.	Documents included in the review: 26<br><br>Studies that evaluated 24-hour Movement in children and/or adolescents: 18	There was not found results specifically for the combination of the three movement behavior in children and/or adolescents.	In combination with evidence-based practical resources to guide the implementation of media planning in the home, reactivation strategies are required to support families to reflect on and rehabituate to 24-hour movement behavior goals in this new era of living with COVID.

Ocvirk, Kovaÿ & Jurak et al. 2021 [38]	To present an overview of the impact of movement restrictions on changes in physical activity, sedentary behavior and sleeping habits of children and adolescents during the COVID-19 epidemic.	Original studies included in the review: 34<br><br>Studies that evaluated 24-hour Movement in children and/or adolescents: did not specify.	During the epidemic, children and adolescents were less physically active, mainly reducing physical activity of moderate to vigorous intensity, due to restrictions on movement and limited opportunities for structured exercise in sports clubs. As a result, they became more sedentary and increased screen time. Additionally, students extended their total sleep time, as the change in school hours allowed them to wake up later in the morning and be to bed later at night. Overall, children and adolescents were less compliant with Canadian 24-hour movement behavior recommendations, which may affect negatively affect their physical fitness and health outcomes.<br>Parents must be educated about the harmful effects of a sedentary lifestyle and inactivity, as their influence is extremely important in achieving the 24-hour movement recommendations for children.	When looking at health as a whole, you need to consider holistic 24-hour behavior: physical activity, sedentary behavior and sleep quality. New habits established by children during the pandemic can become deeply integrated into their lifestyle and remain even after the pandemic, which can have a negative impact on quality of life in the future.
**Research aims: HEALTH AND 24-HOUR MOVEMENT**				
Alanazi et al. 2021 [18]	To investigate the relationship between 24-hour movement behaviors and health indicators in school-age children in Arab countries.	Original studies included in the review: 16<br><br>Studies that evaluated 24-Hour Movement in children and/or adolescents: none	Existing Arabic studies have assessed movement behaviors in isolation from each other. Low levels of physical activity and sleep and high sedentary behavior have been associated with higher levels of body adiposity, behavioral problems, depression and low self-esteem.	The majority of included studies reported favorable associations between movement behaviors and health outcomes. Low levels of physical activity, sleep and/or high sedentary behavior were unfavorably associated with outcomes of body adiposity, behavioral problems, depression and low self-esteem.<br>Further studies to address the literature gap in Arabic-speaking countries, to understand the role of 24-hour movement behaviors and their positive influence on health outcomes in the early years of primary school are urgently needed.
Chaput et al. 2017 [47]	Present evidence that examines how combinations of physical activity, sedentary behavior, and sleep are related to childhood obesity.	Original studies included in the review: did not specify.	The following topics of interest are presented in this narrative review:<br>Definition and operationalization of the terms physical activity, sedentary behavior and sleep; Importance of all	The interactions and clusters between sleep, movement, and non-movement behaviors suggest that all components of the 24-hour movement continuum

	Studies that evaluated 24-hour Movement in children and/or adolescents: did not specify.	movement behaviors in a 24-hour period for optimal health; Combinations of physical activity, sedentary behavior and sleep - relationships with body adiposity in children and adolescents; Knowledge gained from new statistical analysis methods to better understand the combined health effects of sleep, movement and non-movement behaviors; Future research directions.<br>Some highlights of the narrative:<br>“Ignoring these other components of the movement continuum (i.e., sleep, sedentary behavior, and physical activity), which account for approximately 95% of the day, and focusing efforts on moderate to vigorous physical activity, which accounts for approximately 5% of the day, limits optimizing the health benefits associated with movement behaviors.”<br>“Movement behaviors can be combined in many ways”<br>“In addition to optimal balance over 24 hours, movement behaviors also interact with each other.”<br>“It is time to adopt a more inclusive paradigm of all movement behaviors, if we want to maximize health benefits.”<br>“(...) individuals with the ideal combination of behaviors (e.g. high physical activity, low sedentary behavior and long sleep) have less body adiposity and/or risk of overweight than those with a worse combination of movement behaviors (e.g. example, low physical activity, high sedentary behavior, short sleep).”<br>“(...) there are seven potential combinations of the three movement behaviors and, to date, the impact of intermediate combinations (e.g., high physical activity, low sedentary behavior, and insufficient sleep vs. low physical activity, high sedentary behavior, and enough sleep) has not yet been examined.”<br>“Cluster and latent class analysis, isotemporal substitution modeling, and compositional analysis are methods that have recently been introduced as part of the integrated approach.”<br>“Recently, 24-hour monitoring study protocols with devices such as accelerometers have increased the ability to collect data on the three motion and non-motion	should be targeted to optimize health benefits and prevent childhood obesity. In this context, healthy sleep hygiene and reallocating sedentary time to physical activity has also been shown to provide important and valuable positive health benefits. Furthermore, emerging statistical approaches using a compositional paradigm open the door to finding the optimal distribution of time spent in different movement behaviors throughout the day and to inform integrated guidelines.<br>Therefore, public health interventions, guidelines and messages accompanying these actions are expected to target all movement behaviors synergistically to optimize the health of children and adolescents.

			behaviors throughout the 24-hour day, which provides an ideal opportunity for exploration through compositional and other new statistical methods.”	
De Melo et al. 2024 [48]	To explore the clustering profiles of physical activity, sedentary behavior, sleep, and diet in children and adolescents. Additionally, this study aims to explore these clusters according to biological sex and to examine which cluster types are associated with a variety of mental and physical health indicators.	Original studies included in the review: 23.<br><br>Studies that evaluated 24-hour Movement in children and/or adolescents: Three.	Were identified in mixed-sex samples only and the most frequent clusters were “Low Physical Activity, High Sedentary Behavior, Satisfactory Sleep” (n = 3).<br>Most studies in this review analyzed adiposity indicators in youth, which clusters “Low Physical Activity, Sedentary Behavior Satisfactory Sleep” and “Low Physical Activity, High Sedentary Behavior, Satisfactory Sleep” were associated with overweight/obesity and increase in adiposity.	The findings from this review suggest that the co-occurrence of unhealthy behaviors may amplify health risks for youth and the presence of unhealthy behaviors alongside healthy ones highlights the importance of using an integrative approach to understanding how these behaviors collectively influence health.
Fournier et al. 2023 [51]	To analyze data exploring the association between adherence to the 24 h Movement Guidelines and dietary habits in both children and adults, considering the particular effects of the COVID-19 lockdowns and circumstances.	Original studies included in the review: 12<br><br>Studies that evaluated 24-hour Movement in children and/or adolescents: five.	Most young people do not meet the 24 h Movement Guidelines, particularly adolescents, girls and those from countries with a lower human development index. Lifestyle behaviors characterized by low physical activity levels, high sedentary behavior levels and insufficient sleep may lead to numerous health issues. The adherence to none of the recommendations had drastically increased from 28.9% before the pandemic to 66.3% after one year of the pandemic.<br>Limiting screen time habits (and consequently sedentary behavior) was the guideline most strongly associated with a healthier dietary pattern scores both alone and in combination with movement vigorous physical activity recommendations.<br>Adolescents who met all three 24 h Movement Guidelines obtained higher adherence to the Mediterranean diet, were more likely to consume a fruit and vegetables once a day, consume fish regularly, and eat cereal or grains for breakfast and less likely to consume commercially baked goods or pastries for breakfast and to eat sweets and candies several times a day, than those who did not comply with the three 24 h Movement Guidelines.<br>Approximately 6.5% of children and 2.2% of adolescents met all recommendations of the combined movement guidelines.<br>In children, children with overweight or obese fathers and girls had lower odds of adherence to both movement guidelines.	Therefore, combining the 24 h movement recommendations with the dietary recommendations could produce a stronger and better impact on overall adult and youth health than having them separately.

			If children reported regular fruit and vegetables intake, participated in organized physical activity, or if their fathers had a university degree, they had higher odds of meeting specific two-recommendation combinations. Lower odds of meeting specific combinations of recommendations was associated with paternal overweight and obesity and with high sleep efficiency. In adolescents, a correlation between meeting specific combinations of any two recommendations and sex, fruit and vegetables intake, organized physical activity, and active play was highlighted.<br>The median frequency of fruits and vegetable intake was identical between girls and boys. However, compliance with only the screen time recommendation was not associated with fruits and vegetable intake.	
Grgic et al. 2018 [52]	Review and summarize the findings of studies that employed the isotemporal model in research on sleep, sedentary behavior and physical activity. In addition to evaluating the methodological quality of the studies and suggesting future research directions in this area.	Original studies included in the review: 56<br><br>Studies that evaluated 24-hour Movement in children and/or adolescents: three.	Of the total number of studies, three evaluated isotemporal changes in the three movement behaviors in children and adolescents and health outcomes.<br>The most commonly studied health outcomes in relation to isotemporal replacements in children and adolescents were body adiposity and physical fitness.<br><br>The results of studies using the isotemporal substitution model are of great value as they contribute evidence to the construction of meaningful recommendations for increasing physical activity, reducing sedentary behavior, and optimizing sleep duration, considering all behaviors across the entire spectrum. Context of energy expenditure and not just focusing on one behavior in isolation from the others.	There is a notable increase in interest in the associations between the reallocation of time between movement-related behaviors and their relationship with health outcomes. And current evidence suggests that reallocating time between sleep, sedentary behavior, light physical activity, and moderate-to-vigorous physical activity may be associated with health outcomes.<br>The results of this review suggest that it would be more appropriate to shift the focus to the importance of reallocating time spent in sedentary behavior to time spent in physical activity. However, despite current evidence indicating that the reallocation of time between sleep, sedentary behavior, light physical activity, and moderate-to-vigorous physical activity may be associated with a range of health outcomes, longitudinal studies that consider 24-Hour Movement and explore health,

				psychological, social, economic and environmental outcomes, broadly, are needed.
Groves et al. 2024 [53]	To conduct a systematic review to examine how combinations of 24-h movement behaviors relate to indicators of mental ill-being and well-being across the lifespan.	Original studies included in the review: 73<br><br>Studies that evaluated 24-hour Movement in children and/or adolescents: 38	*Cross-sectional studies of children and youth:*<br>*Total guideline adherence* Among cross-sectional studies, 24/38 (63%) of the associations showed that meeting all three guidelines concurrently was correlated with favorable scores for indicators of mental health, whereas 22/38 (58%). When decomposed into indicators of well-being and ill-being, findings revealed 11/14 (79%) associations were favorable effects when meeting three guidelines for indicators of mental well-being. For indicators of mental ill-being, 13/24 (54%) associations demonstrated favorable effects for meeting all three guidelines.<br>*Compositional data analysis* A significant relationship between the 24-h movement composition and indicators of mental health was observed in 11/20 (55%) associations. Examining indicators of mental well-being and ill-being independently revealed a significant relationship for the 24-h movement composition with indicators of mental well-being in 4/7 (57%) associations and 7/13 (54%) associations for indicators of mental ill-being.<br>Isotemporal substitution Replacing sedentary behavior with moderate to vigorous physical activity (14/17 associations; 82%) or sleep (13/17 associations; 76%) were most consistently found to be correlated with more favorable mental health scores. Reallocating sleep to moderate to vigorous physical activity was correlated with more favorable mental health scores in 4/17 (26%) associations, whereas in contrast, reallocating moderate to vigorous physical activity to sleep was correlated with more favorable mental health scores in 5/17 (29%) associations. When decomposed into indicators of well-being and ill-being, results revealed similar patterns. For mental well-being, replacing sedentary behavior with moderate to vigorous physical activity (7/8 associations; 88%) or sleep (6/8 associations; 75%) had favorable effects. For mental ill-being, replacing sedentary behavior	The findings reinforce the notion that time spent engaging in physical activity and sleep need to be prioritized for promoting mental health and well-being, especially when replacing sedentary pursuits such as recreational screen time. Nevertheless, moving forward public health messaging should continue to shift away from a siloed approach focused on individual behaviors to instead emphasize that the whole day counts when it comes to the importance of movement for mental health and well-being.

with moderate to vigorous physical activity (8/9 associations; 89%) or sleep (7/9 associations; 78%) was correlated with favorable scores. Reallocating time from sleep to moderate to vigorous physical activity was beneficial in 3/8 associations (38%) for mental well-being compared to 1/9 associations (11%) for mental ill-being. Replacing moderate to vigorous physical activity with sleep was only beneficial for indicators of mental ill-being (5/9 associations; 56%).

Latent profile or cluster-based analyses. The healthiest combination of movement behaviors (adequate sleep, high moderate to vigorous physical activity, low sedentary time) was correlated with the most favorable mental health scores across 5/5 associations (100%). These findings were consistent for 3/3 (100%) associations investigating indicators of mental well-being and 2/2 (100%) associations investigating mental ill-being.

Longitudinal studies of children and youth

Total guideline adherence For total guideline adherence, meeting one, two or three guidelines concurrently was correlated with favorable scores for indicators of mental health in 3/12 (25%) associations. None of the associations were significant for indicators of mental well-being (0/5; 0%), whereas 3/7 (43%) associations showed favorable effects for indicators of mental ill-being.

Specific combinations of guideline adherence Favorable mental health scores were found in 2/4 (50%) associations for meeting all three guidelines. All associations investigated indicators of mental ill-being.

Compositional data analysis No associations (0/5; 0%) demonstrated a significant relationship for the 24-h movement composition with indicators of mental well-being (0/2 associations; 0%) or mental ill-being (0/3 associations; 0%).

			Isotemporal substitution Akin to the cross-sectional studies among children and youth, replacing sedentary behavior with MVPA (8/8 associations; 100%) or sleep (7/8 associations; 89%) were most consistently found to be correlated with more favorable mental health scores. Comparatively, 5/8 (63%) associations found that replacing moderate to vigorous physical activity with sleep was correlated with better scores for mental health, whereas only 2/8 (26%) associations showed replacing sleep with moderate to vigorous physical activity was correlated with more favorable mental health scores. All associations investigated indicators of mental ill-being.<br><br>Latent profile or cluster-based analyses. The healthiest combination of movement behaviors (adequate sleep, high moderate to vigorous physical activity, low sedentary time) was associated with the most favorable mental ill-being scores in 1/1 (100%) associations, whereas no differences were observed across the other behavioral combination profiles. Associations with indicators of mental well-being have not been examined.	
Huang et al. 2024 [20]	To assess the prevalence of meeting the overall and individual 24-h movement guidelines, and examine the associations between meeting 24-h movement guidelines and health indicators in people with disabilities.	Original studies included in the review: 24<br><br>Studies that evaluated 24-Hour Movement in children and/or adolescents: 18	Regarding health indicators, 10 studies reported at least one health indicators (e.g., quality of life, obesity, mental health).<br>Subgroup analyses revealed that the 6–17 years group had a significantly lower overall prevalence (5.1%) than that of the 18–65 years group (16.49%).<br>Ten studies investigated the associations between meeting different combinations/number of the 24-h movement guidelines and health indicators in children and adolescents with disabilities.<br>Of these, three studies found that meeting all three guidelines was significantly associated with lower odds of having overweight or obesity than meeting none.<br>Another two studies found that meeting 24-h movement guidelines was not significantly associated with the odds of being overweight or obese among children and adolescents with intellectual disability or attention deficit/hyperactivity disorder.	There is some evidence showing that the prevalence of meeting all three 24-h movement guidelines in people with disabilities is low; meanwhile, age and disability type are moderators influencing the overall guidelines adherence. There is preliminary evidence suggesting that people with disabilities meeting all three guidelines have better psychosocial health than those meet none.

			One study using compositional analysis found that reallocating time from physical activity and sedentary behavior to sleep duration decreased body mass index in children and adolescents with autism spectrum disorders, and vice versa.<br>Eight studies examined the relationship between meeting 24-h movement guidelines and psychosocial health (e.g., quality of life, depression, social relationship).<br>Two studies found that participants with disabilities meeting all three guidelines had significantly higher quality of life than those meeting none of the guidelines.<br>one study showed that meeting all three guidelines was associated with lower odds of having cognitive and social difficulties in relation to meet none of the guidelines in children and adolescents with intellectual disability or attention deficit/hyperactivity disorder. Similarly, a final study found that meeting all three guidelines was significantly associated with higher flourishing level compared to meeting none or only one guideline in youth with intellectual disability or attention deficit/hyperactivity disorder.	
Julian et al. 2022 [28]	The present narrative review proposes a specific focus on movement behaviors, the available evidence linking these behaviors to general health, how movement behaviors are associated with eating patterns/appetite control, and the potential mechanisms elucidated to date in children and adolescents. with obesity.	Original studies included in the review: did not specify.<br><br>Studies that evaluated 24-hour Movement in children and/or adolescents: did not specify	Although many studies have considered energy expenditure and intake separately, more recent work has begun to uncover interactions between activity levels and eating behaviors. In this context, early identification of behavioral phenotypes and a comprehensive approach addressing all key behaviors that directly affect energy balance will enable individual strategies to prevent or treat obesity and its comorbidities.<br>Children's 24-hour movement behaviors, combining measures of physical activity, sedentary behavior, and sleep, have attracted increasing interest in both public health research and clinical practice, allowing scientists and practitioners to unravel the independent and combined effects of these behaviors.<br><br>"It is important to highlight that the behavior associated with overweight and obesity continues throughout life."	This review presents current definitions and recommendations for movement behaviors, including physical activity, sedentary behavior, and sleep, in children and adolescents. It also summarizes current evidence on the negative impact of undesirable movement behavior patterns in childhood that carry over into adolescence and adulthood on key childhood health outcomes that extend into adolescence and adulthood.
Kracht et al. 2024 [57]	To characterize the breadth, and scope of systematic reviews and	Original studies included in the review: 32.	Physical activity, sedentary behavior, and sleep were clearly defined in less than a third of the reviews.	This umbrella review revealed that the breadth and scope of existing literature

	meta-analyses examining at least two of the physical activity, sedentary behavior, and sleep concurrently. To examine prevalence estimates for 24-hour movement guideline adherence. To examine associations with health outcomes by various approaches.	Studies that evaluated 24-hour Movement in children and/or adolescents: 20	In general, reviews found around half of children met the physical activity guideline (estimate range: 22.3–67%), less than a third met the screen-time guideline (estimate range: 10-28.3%), and half or more met the sleep guideline (estimate range: 57-83.5%).<br>Differing approaches to 24-hour movement behavior research provide an opportunity to answer unique questions regarding the collective influence of these behaviors on health, further deepening our understanding of the implications of behavioral time-use across the course of a 24-hour day. High amounts of movement vigorous physical activity, reallocating sedentary behavior to movement vigorous physical activity, and meeting all three 24-hour movement guidelines demonstrated clear health benefit, with less certainty for sedentary behavior and sleep. This review demonstrates that systematic reviews, and hence our understanding, of the influence of 24-hour movement behaviors on health is in its nascency, with opportunities to increase future review's representation, rigor, and reporting.<br>Despite the many approaches used, these 24-hour movement behavior reviews consistently found high amounts of movement vigorous physical activity and meeting all three guidelines were beneficial for various indicators of health. The current yield of systematic reviews also found more reviews investigated physical health outcomes relative to mental health outcomes.	on 24-hour movement behaviors is wide; this literature spans all ages and regions in different capacities. Included reviews permitted many definitions and approaches to analyzing associations between 24-hour movement behaviors and health outcomes and were overall low quality; both qualities hindered harmonized synthesis. Amongst these weaknesses, a consistent finding was improved health benefit from additional movement vigorous physical activity and meeting all three guidelines, with inconsistent findings for sedentary behavior and sleep. Given the collective and individual benefit of these behaviors, the next decade should focus on harmonized rigorous research using a multi-behavior approach to improve existing evidence.
Lannoy et al. 2023 [8]	Identify what evidence there is on the association between 24-hour movement behaviors and mental health in children/adolescents and what integrated knowledge mobilization applications/tools exist.	Original studies included in the review: 55<br><br>Studies that evaluated 24-hour Movement in children and/or adolescents: 42	Of the total number of studies, 36 were carried out with children (from five to 11 years old) and 36 with adolescents (from 12 to 17 years old), a total of 48 studies (41 cross-sectional, 6 longitudinal and 1 longitudinal and cross-sectional).<br>*Measurement of movement behaviors:*<br>**Physical activity** – in 13 studies using an accelerometer, in 25 by self-report and 12 by proxy report.<br>**Sedentary behavior** – in seven studies using an accelerometer, in 26 by self-report, 19 by proxy report and in one by a *Global Positioning System*.	Considerable evidence of the association between combined movement behaviors and mental health was identified, with the majority suggesting a positive (beneficial) association between 24-hour movement behavior guidelines among children and young people and mental well-being, and a negative/inverse association (but still beneficial) with indicators of mental illness. However, only one instrument focused on combined movement behaviors and mental health

			**Sleep** – in nine studies using an accelerometer, in 22 by self-report, 22 by prosecutor report and one by application linked to the phone.<br>Of the 48 articles, 38 reported associations (positive or negative) between all three movement behaviors combined and mental health indicators. Regarding mental well-being indicators, 17 reported a positive association between combined movement behaviors and mental well-being, while five reported no association. Regarding mental illness indicators, 21 reported an inverse association between combined movement behaviors and mental illness (i.e., compliance with the three guidelines was associated with lower scores on mental illness indicators), and two articles reported no association.<br>Through environmental scanning, only one article/tool was identified that explored a composite score of combined movement behaviors in relation to mental health.	was identified. Future efforts are needed to monitor population and individual 24-hour movement behaviors through a user-friendly index to assist individuals and families in recalibrating their movement behaviors for the promotion of mental health and disease prevention, guaranteed. Such a tool can lead to understandings and interventions, personalized management and modification of human movement to promote health, prevent disease and preserve well-being.
Lee et al. 2024 [56]	To examine the relationships between climate change, 24-hour movement behaviors, and health.<br>Given the potential bidirectionality between climate change and movement behaviors, this systematic review focused on: (1) clarifying the potential role of 24-hour movement behaviors in mediating/moderating the relationship between climate change and health and (2) clarifying the potential impact of climate change on the relationship between 24-hour movement behaviors and health.	Original studies included in the review: 79<br><br>Studies that evaluated 24-hour Movement in children and/or adolescents: none	No studies have addressed a combination of all 24-hour movement behaviors in children and/or adolescents.	This review did not concluded about 24-hour movement behavior in children and/or adolescents.
Lópes-Gil et al. 2023 [45]	To determine the association between meeting all three 24-hour movement recommendations and obesity-related indicators among young people aged 3 to 18 years.	Original studies included in the review: 29<br><br>Studies that evaluated 24-hour Movement in children and/or adolescents: 21	For each obesity-related indicator separately, meeting all 24-hour movement recommendations was associated with a lower likelihood of both overweight/obesity and obesity alone. Additionally, there was a negative relationship between 24-hour movement recommendations and body mass index, z body mass index, waist circumference, and body fat percentage.	Meeting all 24-hour movement recommendations may be a crucial factor in maintaining a healthy weight status in the young population. Our findings highlight that all components of the 24-hour movement continuum should be addressed jointly to prevent

			Regarding subgroup analyses, the association between 24-hour movement recommendations and overall obesity-related indicators was similar regardless of sex and comparison (meeting all three vs. not meeting). In contrast, that association was higher in children and adolescents in comparison with preschoolers and using self-reported measures in comparison with accelerometer-based measures to assess 24-hour movement recommendations.<br>Regarding longitudinal design, meeting all three 24-hour movement recommendations was not negatively associated with obesity-related indicators. Additionally, the sensitivity analyses indicated no modifications in the results after removing one study at a time. The Luis Furuya–Kanamori index for the Doi plots showed asymmetry, verifying the presence of publication bias. There was no significant association between 24-hour movement recommendations and body mass index z-score.	overweight and obesity from early childhood to adolescence, which is one of the greatest challenges to public health in the 21st century.
Marques et al. 2023 [36]	To evaluate the results of published studies that analyse the relationship between compliance with the 24-h movement guidelines and overweight and obesity in toddlers, children and adolescents.	Original studies included in the review: 24<br><br>Studies included in meta-analysis: 12<br><br>Studies that evaluated 24-hour Movement in children and/or adolescents: 14	For toddlers, two studies identify associations between meeting the 24-h movement guidelines and body mass index z-score were null in both studies that analysed this relationship.<br><br>Seven studies analysed the relationship between compliance with the 24-h movement guidelines and overweight and obesity among preschool children.<br>Only one study found that those who did not meet all the 24-h movement guidelines had higher odds of being overweight or obese than those who met the guidelines.<br><br>Two studies found that children and adolescents who met the 24-h movement guidelines were more likely to have lower risks of overweight and obesity.<br><br>Six articles showed no differences in overweight and obesity levels in children and adolescents who comply and do not comply with all 24-h movement guidelines.	Most included studies have not observed a significant relationship between compliance with the 24-h movement guidelines and overweight and obesity in toddlers, children and adolescents. This is more evident in toddlers and preschool children.<br>Two elements are critically needed to understand better the relationship between compliance with the 24-h movement guidelines and overweight and obesity: future longitudinal research design and the integration of more accurate methodologies to assess screen time and sleep time (including technological sensors).

			In one of the articles, the analyses were stratified by age, analysing children, early adolescents and adolescents.	
Patience et al. 2023 [22]	Conduct a systematic review of quantitative and qualitative studies to comprehensively investigate 24-hour movement behaviors (individual and/or combined) and their impact on primary metrics (glycated hemoglobin (HbA1c) and continuous glucose monitoring (CGM) and quality of life (QoL)) and secondary outcomes (depressive symptoms, anxiety, stress/distress, self-management, coping, self-efficacy in diabetes, family functioning, social competence) in adolescents with Type 1 Diabetes Mellitus (DM1).	Original studies included in the review: 84<br><br>Studies that evaluated 24-hour Movement in children and/or adolescents: none	Only 2.38% (n = 2) of studies investigated all three movement behaviors, but none combined.	No studies to date have investigated how combinations of behaviors collectively interact and impact any of the primary or secondary outcomes in adolescents with type I diabetes. Monitoring the full spectrum of 24-hour movement behaviors would allow a comprehensive understanding of how the accumulation and the weighting of each behavior can interact and impact important outcomes for adolescents with type I diabetes. Future research should investigate the association between 24-hour movement behaviors (measured via accelerometer), glycemic control and psychosocial outcomes. Furthermore, measuring glycemic control through glycated hemoglobin glucose metrics and continuous glucose monitors would aid in comprehensive investigation by providing a detailed objective and continuous pattern of glucose throughout the 24-hour period. Finally, qualitative studies investigating knowledge, awareness, and feasibility of a 24-hour behavioral movement approach would help understand how adolescents with type I diabetes could adopt this type of approach.
Rollo, Antsygina & Tremblay, 2020 [7]	Synthesize and critique current evidence on adherence to 24-hour movement guidelines, correlates and associations with health indicators across the lifespan, as well as relationships between the composition of 24-hour movement behaviors and health indicators.	Original studies included in the review: 51 (14 with children, four with young people and eight with children and young people).<br><br>Studies that evaluated 24-hour Movement in children and/or adolescents: 26	Of the total original studies, 19 studies examined adherence (meeting vs. non-meeting) to 24-hour movement guidelines (seven with children, four with youth, and eight with children and youth) and seven studies used compositional analyzes to explore the composition of 24-hour time use of movement behaviors (six with children and one with children and young people).	The composition of movement behaviors within a 24-hour period may have important implications for health across all ages, and meeting current 24-hour movement guidelines is associated with a number of desirable health indicators in children and youth. In summary, the findings demonstrate that

Measurement of movement behaviors:

Physical activity – in eight studies it was measured objectively by accelerometers and in one study by pedometers, in addition to being self-reported in nine studies and reported by parents in one study.

Sedentary behavior and/or screen time – were self-reported in 17 studies and parent-reported in three studies. One study included self-reported and parent-reported measures due to the age of the participants.

Sleep – objectively measured in five studies, self-reported in 12 studies, and parent-reported in three studies. Only two studies included objective, self-reported measures of sleep, likewise, two studies included self-reported and parent-reported measures due to the age of the participants.

*The remaining seven studies carried out compositional analyzes (six with children and one with children and young people).

Associations between the composition of movement behaviors and health indicators (six studies):

Adiposity (five studies with children and one with children and young people) – These studies ranged from fair to good quality. Three studies reported unfavorable differences in indicators of adiposity (e.g., body fat percentage) when time was reallocated from moderate-to-vigorous physical activity to any other movement behavior. In one study, the composition of 24-hour movement behaviors was associated with body mass index, waist circumference and fat mass index. And in another study, the use of weather composition explained between 9% and 35% of the increase in the risk of obesity in children, depending on ethnicity.

One study included a sample of children and young people, and reported that the composition of movement behaviors was significantly associated with body mass index z-scores and waist circumference.

Cardiometabolic Health (a study of children and adolescents) – In this high-quality study, the composition of movement behaviors was associated with blood

school-aged children and youth who meet all 24-hour movement guideline recommendations generally have more favorable measures of adiposity; mental, social, emotional and cardiometabolic health, physical fitness, perceived health and cognitive development and have healthier eating patterns than those who do not meet recommendations.

There is a need for additional high-quality investigations employing longitudinal and experimental study designs, using valid and reliable movement behavior measures (physical activity, sedentary behavior, and sleep), and examining a wide range of health indicators across all ranges. ages. Such studies would confirm the mostly cross-sectional evidence and further advance our understanding of the relationships between 24-hour movement behaviors and health.

pressure, triglycerides, high-density lipoprotein, C-reactive protein, and insulin.

Physical fitness (one study of children and one study of children and adolescents) - Among children, replacing moderate to vigorous physical activity with any other movement behavior predicted lower cardiorespiratory fitness. Among children and adolescents, the composition of movement behaviors was associated with aerobic fitness.

Mental, social and emotional health (a study of children and adolescents) – in this high-quality study the composition of movement behaviors was associated with behavioral strengths and difficulties.

Perceived health (a study with children) – in this good quality study, with an international sample of children, those from countries with higher human development indices, the composition of movement behavior was significantly related to health-related quality of life, where moderate to vigorous physical activity related to sleep and sedentary behavior was positively associated with this health indicator.

Summary: In children, consistent evidence of adiposity and preliminary evidence of health-related quality of life and cardiorespiratory fitness were found, in which the composition of movement behaviors, specifically greater moderate to vigorous physical activity relative to other behaviors, were associated with favorable measurements. For both children and youth, there was initial evidence that the composition of 24-hour movement behaviors, specifically greater moderate-to-vigorous physical activity, more sleep, less light physical activity, and/or sedentary behavior, relative to other behaviors, was associated with favorable indicators of adiposity and aerobic fitness, as well as cardiometabolic, social and emotional health.

Associations between meeting the 24-Hour Movement Guidelines and health indicators (six studies):

Body adiposity (two studies of children) – both studies reported favorable associations between meeting all three

recommendations and indicators of adiposity (body mass index z-score and odds of obesity). One study reported that compared to meeting all three recommendations, meeting two, one, or none of the guidelines was associated with a 2.5-, 4.5-, and 8.0-fold increased odds of obesity, respectively. One study examined the relationship between adherence to 24-hour movement guidelines and indicators of adiposity in adolescents and reported that boys and girls who did not meet any of the recommendations were 4.0 and 3.8 times more likely to be obese, respectively, compared to those who met the three guideline recommendations. Finally, relationships between adherence to 24-hour movement guidelines and indicators of adiposity in samples including children and youth were examined in three studies, all of which reported favorable associations between adherence to all 3 recommendations and indicators. Adiposity or risk of overweight/obesity.

Mental, social, and emotional health (one study of children, one of adolescents, and two of children and adolescents) – Of these good-quality studies, one reported that adherence to all three recommendations was associated with lower scores of positive urgency, negative urgency and behavioral inhibition system, higher perseveration scores and better delay discounting scores in children. One study reported that meeting all three recommendations was significantly associated with being happy and not feeling stressed in adolescents. Furthermore, when the overall mix of movement behaviors was considered, complying with more recommendations, compared with none, was significantly and incrementally associated with being happy.

Furthermore, compliance with all three recommendations was associated with better scores on strengths and behavioral difficulties, lower scores on emotional problems and higher scores on life satisfaction and prosocial behavior in children and young people.

Cardiometabolic health (two studies with children and adolescents) – One fair-quality study and one good-quality study reported that meeting the three

recommendations was associated with lower insulin levels, lower triglyceride levels (in both), and lower glucose levels. One study of the studies reported favorable associations with blood pressure and high-density lipoprotein cholesterol, but the other study found no associations.

Cognitive development (a study of children) – This study was of fair quality, and reported that each additional recommendation met, as well as meeting all three recommendations, was positively associated with global cognition.

Perceived health (a study of children) – This good quality study reported that meeting all three recommendations of the 24-hour movement guidelines was associated with significantly better health-related quality of life when compared to meeting none of the recommendations. Furthermore, differences between countries have been reported in the relationships between health-related quality of life and adherence to 24-hour movement guidelines.

Aerobic fitness (a study of children and adolescents) – This good quality study reported that those who met all three recommendations had greater aerobic fitness than those who met none, one and two recommendations.

Dietary patterns (a study of children) – This fair quality study reported that a healthier dietary pattern was observed when more movement behavior recommendations were met.

Summary: For children and youth, there was consistent evidence that those who adhered to all three 24-hour Movement Guidelines had lower adiposity and lower odds of obesity, as well as favorable indicators of mental, social, and emotional health (e.g., impulsivity, psychological well-being, prosocial behavior). Compliance with integrated guidelines was also associated with greater aerobic fitness and favorable cardiometabolic health. Specifically for children, there was preliminary evidence that those who met guideline recommendations reported better global cognition, better health-related quality of life, and healthier eating patterns.

Sampasa-Kanyinga et al. 2020 [41]	To examine how combinations of physical activity, sedentary time, and sleep duration relate to depressive symptoms in children and adolescents. Secondary objectives examined associations with a broader spectrum of mental health indicators.	Original studies included in the review: 10<br><br>Studies that evaluated 24-hour Movement in children and/or adolescents: three	The 10 cross-sectional studies that reported mental health outcomes were published in 2016 or later. The sample totaled 84,004 children and adolescents.<br>The proportion of children and adolescents who met the three recommendations ranged from 2.6 to 17.1%.<br>*Measurement of movement behaviors:*<br>**Physical activity** – in five using accelerometers and self-reported or reported by parents in another five.<br>**Sedentary time** – in three studies it was measured objectively using accelerometers and self-reported in seven studies.<br>**Sleep duration** – objectively measured in two studies and self-reported or parent-reported in eight studies.<br>Three studies examined the association between a combined measure of meeting all three movement behavior recommendations and depressive symptoms and identified the following results: Depressive symptoms were associated with lower odds of meeting all three 24-Hour Movement Guidelines in male adolescents and feminine. Meeting all three 24-hour movement guidelines was associated with lower odds of anxiety and depression among adolescents (i.e., ages 12 to 17) compared to meeting none of the recommendations. Compliance with all three recommendations was associated with significantly lower odds of depressive symptoms among adolescents than compliance with none, one, or any combination of two recommendations among adolescents. However, among children (i.e., ages six to 11), meeting only the screen time recommendation, both the screen time and sleep duration recommendations, or both the physical activity and sleep duration recommendations, was associated with lower odds of depressive symptoms than not meeting any or all three guidelines. For other mental health indicators, studies were consistent in showing better mental health indicators in participants who met all three recommendations compared to those who met none of them. **Collectively, the results indicate favorable associations between compliance with the three recommendations and better mental health indicators among children and adolescents	This review provides evidence that adherence to the 24-Hour Movement Guidelines for children and adolescents is associated with better mental health status. These findings reinforce the need to encourage children and adolescents to comply with the 24-Hour Movement Guidelines. It is important that all interested parties, including parents, schools, caregivers, health professionals, political actors and children and adolescents themselves, are informed about the potential benefits of adhering to the 24-Hour Movement Guidelines. However, the available evidence is of very low quality as it is based on cross-sectional studies using self-reported measures of physical activity, screen time and sleep duration. Higher quality research is desired to determine whether a dose-response gradient exists between the number of movement behavior recommendations met and mental health to better support the 24-Hour Guideline paradigm.

			when compared with non-compliance with the recommendations.	
Saunders et al. 2016 [3]	Determine how combinations of different levels of physical activity, sedentary behavior and sleep were associated with health indicators among children and young people aged 5 to 17 years.	Original studies included in the review: 14<br><br>Studies that evaluated 24-hour Movement in children and/or adolescents: four.	The four studies included a total sample of 2,598 children and adolescents. Of these studies, 10 were on adiposity; three cardiometabolic risk factors, three on cardiorespiratory or musculoskeletal fitness and two reported more than one indicator. No articles were identified that reported the relationship between combinations of movement behaviors and emotional regulation/psychological distress, behavioral conduct/prosocial behavior, cognition, quality of life/well-being or injuries.<br>*Measuring behaviors:*<br>**Physical activity** – in one study pedometer and accelerometer in three studies.<br>**Sedentary behavior** – self-reported questionnaire in two studies and accelerometer in three (two studies used two forms of measurement)<br>**Sleep** – in three accelerometer studies and in one self-reported.<br><br>**Adiposity** – Three studies reported that children and adolescents with a combination of high physical activity, low sedentary behavior and high sleep duration had lower adiposity and/or risk of overweight than those with a combination of low physical activity, high sedentary behavior and low sleep duration. A study of the studies also reported that compared to meeting the three recommended guidelines for steps/day, screen time, and sleep, meeting 2, 1, or none of the guidelines was associated with 2.6, 4.7, and 8. .2 times more likely to be obese, respectively.<br>**Cardiometabolic health** – Evaluated in a longitudinal study with 200 days of follow-up of 632 Danish children aged between 8 and 11 years. Compared to children who increased moderate to vigorous physical activity and sleep and reduced sedentary behavior, children who reduced physical activity and sleep while increasing sedentary behavior had a 3.31 unit increase in metabolic syndrome score (calculated as the sum of z-scores for waist	The results suggest that school-age children and adolescents, characterized by reduced time in sedentary behavior and high levels of physical activity and sleep duration, generally present more desirable measures of adiposity and cardiometabolic health, when compared to those who report a combination of longer time in sedentary behavior and lower level of physical activity and sleep duration. The overall quality of available research evidence was low. Longitudinal and intervention studies, and those using new statistical methodologies, are needed to further clarify these relationships and determine the potential health benefits of various combinations of movement behaviors.

			circumference, mean arterial pressure, homeostasis model assessment of insulin resistance, triglycerides, and high-density lipoprotein cholesterol) during the 200-day follow-up.<br>The evidence for both indicators was considered to be of low quality.	
Wilhite et al. 2023 [17]	To evaluate combinations of physical activity, sedentary behavior, and sleep duration (defined as “movement behaviors”) and their associations with physical, psychological, and educational outcomes in children and adolescents.	Original studies included in the review: 141 (2 conference abstracts).<br><br>Studies that evaluated 24-hour Movement in children and/or adolescents: 16	All studies investigating combinations of all three movement behaviors were cross-sectional.<br>**Results related to physical health:**<br>1) Body adiposity: in 11 cross-sectional studies, the combination of high physical activity, low sedentary behavior and high sleep duration had the best association with adiposity, while the remaining five studies had mixed results.<br>2) Cardiometabolic risk factors: a (cross-sectional) study found different results when combining the three behaviors, depending on the specific result of replacing sedentary behavior with sleep.<br>3) Cardiorespiratory fitness: two studies, one longitudinal and one cross-sectional, indicated that children who were more active and slept more than their peers had better cardiorespiratory fitness. No study included sleep in the composition of behaviors for adolescents.<br>4) Muscular fitness: a cross-sectional study reported that children who combined physical activity and sleep recommendations had more strength, better endurance and flexibility than their peers, compared to any other combination of movement behaviors.<br><br>**Psychological results**<br>1) Well-being and socio-emotional outcomes: four cross-sectional studies reported a positive association for the combination of high physical activity, low sedentary behavior and high sleep duration with well-being.<br>2) Health-related quality of life: a cross-sectional study reported that the combination of meeting the recommendations of the three movement behaviors had the best result for health-related quality of life.<br>3) Mental health: five cross-sectional studies reported that movement behaviors are associated with better mental	Evidence suggests that physical activity, sedentary behavior and sleep duration should be investigated in combination, rather than in isolation. Due to sleep's consistent positive associations with a variety of outcomes, researchers are encouraged to consider sleep in their studies of movement behaviors. Public health guidelines, interventions, and campaigns must look beyond promoting single movement behaviors and move toward targeting all three. The needs of children and adolescents could be better considered. Children, adolescents, parents and schools must be informed that physical activity, sedentary behavior and sleep affect physical health as well as psychological health and educational development.

health, and that high sleep duration exerts a protective function for this outcome.

Education-related results

1) Academic performance: A (cross-sectional) study reported that meeting the recommendations for the combination of the three movement behaviors was the most beneficial for the association with academic performance.

2) Executive/cognitive function: Two studies considered the three behaviors in association with cognitive function and impulsivity, and meeting screen time and sleep recommendations was considered to have the most significant association.

Zhao et al. 2024 [35]	To synthesize existing evidence for children and adolescents 1) to assess adherence and conduct a meta-analysis of global 24-h movement guidelines and 2) to determine the association between meeting both the general combination and the specific combination of 24-h movement guidelines with health outcomes.	Original studies included in the review: 61<br><br>Studies that evaluated 24-hour Movement in children and/or adolescents : 39	The total of 39 studies reported overall adherence to 24-h movement guidelines. Regarding age group, 26 studies focused on children and 22 studies focused on adolescents. In terms of gender, 19 studies included both boys and girls. In terms of geographical region, 18 different countries were identified, including 3 in Africa, 20 in Asia, 10 in Europe, 4 in Oceania, and 22 in North America.<br>The proportions of children and adolescents the general combination of 24-h movement guidelines were 7.1 ± 1.0%.<br>Adherence to guidelines was higher in male subjects than in female subjects and in children than in adolescents. Regarding geographical regions, were noted variations in overall adherence, with lower rates observed in South America (3.2%) and higher in Europe (14.3%).<br>Of these studies, 10 of 14 reported that meeting the general combination was associated with a lower risk of body mass index or body mass index z-score or being obese or overweight.<br>Three of four studies reported that adherence to general combinations was associated with lower waist circumference.<br>Three of five studies conducted on meeting general combinations presented an association with lower body fat, and two of five studies showed no association.<br>Three longitudinal studies conducted on meeting all three guidelines at baseline were inversely associated with adiposity at 2-year follow-up.<br>Four studies suggested that meeting either the individual or combined guidelines did not show significant associations with most cardiometabolic biomarkers, such as blood pressure, subcutaneous adipose tissue, visceral adipose tissue, triglycerides, high density lipoprotein cholesterol, insulin, and glucose levels.<br>One longitudinal study revealed that meeting all three guidelines at baseline was inversely associated with insulin and C-reactive protein, with no association found for glucose, triglycerides, high density lipoprotein cholesterol, systolic blood pressure, or diastolic blood	None of the questionnaires was considered sufficiently valid and/or reliable to assess one or more movement behaviors in children aged zero to five years. The lack of high-quality methodological studies evaluating all relevant measurement properties of the developed questionnaires hampers the ability to draw definitive conclusions about the best available questionnaires. Questionnaires to assess 24-hour movement behaviors in children aged zero to five years are scarce. Therefore, high-quality studies are needed with the objective of developing *proxy-report questionnaires* for this age group and evaluating their measurement properties, based on content validity.

pressure at 2-year follow-up. The specific combination emphasized moderate vigorouse physical activity and moderate vigorouse physical activity and screen time guidelines.

Four studies suggested that meeting guidelines were associated with a higher level of general fitness, cardiorespiratory fitness, muscular strength, speed, and agility; it was not associated with grip strength, sit-up, sit-andreach, 20-m shuttle run, or flexibility.

Eight studies showed that meeting all three guidelines was associated with lower emotional problems, not feeling stressed, fewer internalizing and externalizing behaviors, decreased loneliness and sadness, higher perceived self-efficacy, fewer suicidal ideation and suicide attempts, higher positive psychosocial health, higher prosocial behavior, higher satisfaction, lower depressive symptoms, and anxiety.

Five studies indicated that non-compliance with any of the guidelines was associated with higher scores in strengths and difficulties, increased prosocial behavior, lower life satisfaction, little happiness, higher risk of internet addiction, elevated levels of anxiety, and depression.

Three studies reported that adherence to meeting all three guidelines had better health-related quality of life.

One study revealed adolescents who met all three guidelines at baseline displayed lower anxiety and depression symptoms at six months.

One study revealed adolescents who met all three guidelines at baseline displayed lopes at six months. Another study emphasized that movement vigorous physical activity and sleep at baseline was inversely associated with cognition, psychosocial, and gray matter volumes at 2-year follow-up.

Two studies reported that meeting all three guidelines was associated with higher academic achievement, and one study showed that meeting at least two out of the three guidelines was associated with better academic achievement.

			Regarding perceived health two studies showed that meeting all three guidelines did not show any association with perceived health when compared to meeting none.<br>The associations between adherence to the 24-h movement guidelines and dietary patterns revealed that meeting a higher number of guidelines was linked to improved dietary patterns.	
**Research aims: OTHER 24-HOUR MOVEMENT ASSOCIATIONS**				
Bao et al. 2024 [23]	To quantitatively synthesise the association between adherence to the 24-h movement guidelines and academic-related outcomes in children and adolescents.	Original studies included in the review: ten.<br><br>Studies that evaluated 24-hour Movement in children and/or adolescents: ten.	The meta-analysis suggests a small relationship between adherence to all three recommendations and academic achievement compared to those who did not adhere to any recommendations.<br>Not adhering to the 24-hour movement guidelines was associated with poor executive functions, shifting efficiency, and non-preservative errors compared to those who did adhere to the 24-hour movement guidelines. And adhering to the 24-h movement guidelines, when compared to not adherence to none, was positively associated with composite cognition scores in cross-sectional study, and composite cognitive function at baseline, but not during follow-up in longitudinal study.<br>Due to the limited number of studies included and their low quality, this association should be interpreted with caution.	The meta-analysis suggests a small but positive association between adherence to all three recommendations of the 24-h movement guidelines and greater academic achievement in children and adolescents. However, due to the limited number of studies and their heterogeneity, this finding should be interpreted with caution.
Jiang et al. 2024 [55]	To assess the effectiveness of eHealth interventions on 24-hour movement behaviors (improving physical activity and sleep duration and decreasing sedentary time), and to examine the moderating effects of study characteristics (eg, intervention duration, intervention type, and outcome measurement tools) on intervention effectiveness.	Original studies included in the review: 24<br><br>Studies included in meta-analysis: 13<br><br>Studies that evaluated 24-hour Movement in children and/or adolescents: none.	No studies examined all three movement behaviors.	As a result, there is a pressing need for rigorous and high-quality research endeavors to develop eHealth interventions capable of effectively enhancing both the quantity and quality of 24-hour movement behaviors simultaneously. These interventions should strive to maintain their effects over extended periods.
Maddren et al. 2024 [25]	To examine the associations between pollution measures (air, water, land, and noise), (1) 24-hour movement behaviours (physical activity, sleep,	Original studies included in the review: 18	No studies have addressed a combination of all 24-hour movement behaviors in children and/or adolescents.	A holistic approach that considers environmental factors (such as weather conditions) and the child's socioecology can provide further

	and sedentary behaviour), and (2) motor development outcomes (fine and gross motor skills), among children from birth to 12 years of age.	Studies that evaluated 24-hour Movement in children and/or adolescents: none.		insight into the associations between postnatal pollution exposure, the 24-h movement behaviours. There is limited evidence regarding associations between pollution measures, 24-h movement behaviours. Future research should pay more attention to postnatal exposure to different types of pollution and its impact on healthy levels of physical activity, sedentary behaviour, sleep, and consider confounders such as geographic location, weather conditions, and country income level.

*LGBTA+: lesbian, gay, bisexual, transgender, queer (or questioning), and asexual.
