# Supplementary material for: Mapping Evidence on Integrated 24-Hour Movement Behaviors in Children and Adolescents: A Scoping Review of Reviews
Source: Children (Basel). 2025 Feb 20;12(3):260. doi: 10.3390/children12030260 (PMC11940917; doi:10.3390/children12030260)
Supplement: Supplementary file 1 [file children-12-00260-s001.zip › Table S5 Quality and risk of bias.pdf]

Table S5. Quality and risk of bias in the systematic review studies and systematic reviews with meta-analysis of included studies (n = 28).

Author(s)	Q1	Q2	Q3	Q4	Q5	Q6	Q7	Q8	Total score
Alanazi et al. 2021 [18]	✓	✓	✓	✓	✓	✓	×	NA	6
Arts et al. 2022 [46]	✓	✓	✓	✓	✓	✓	×	NA	6
Bao et al. 2024 [23]	✓	✓	✓	✓	✓	✓	✓	✓	8
Chong et al. 2020 [24]	✓	✓	✓	✓	✓	✓	×	NA	6
De Melo et al. 2024 [48]	✓	✓	✓	✓	✓	✓	×	NA	6
Feng et al. 2024 [49]	✓	✓	✓	✓	✓	✓	✓	✓	8
Fortnum et al. 2024 [50]	✓	✓	✓	✓	✓	✓	×	NA	6
Groves et al. 2024 [53]	✓	✓	✓	✓	✓	✓	×	NA	6
Hao et al. 2024 [54]	✓	✓	✓	✓	✓	✓	NA	✓	7
Hartson et al. 2023 [21]	✓	✓	✓	✓	✓	✓	×	NA	6
Huang et al. 2024 [20]	✓	✓	✓	✓	✓	✓	✓	✓	8
Jiang et al. 2024 [55]	✓	✓	✓	✓	✓	✓	✓	✓	8
Jurakiÿ & Pedišiÿ et al. 2019 [11]	✓	✓	✓	✓	×	✓	×	NA	5
Lee et al. 2024 [56]	×	✓	✓	✓	✓	✓	✓	✓	7
Lettink et al. 2022 [15]	✓	✓	×	✓	✓	✓	×	NA	5
Lópes-Gil et al. 2023 [45]	✓	✓	✓	✓	✓	✓	✓	✓	8
Maddren et al. 2024 [25]	✓	✓	×	✓	✓	✓	×	NA	5
Marques et al. 2023 [36]	✓	✓	✓	✓	✓	✓	×	✓	7
Neville et al. 2024 [37]	✓	✓	×	✓	✓	✓	✓	✓	7
Patience et al. 2023 [22]	✓	✓	✓	✓	×*	✓	×	NA	5
Rodrigues et al. 2023 [39]	✓	✓	✓	✓	✓	✓	×	NA	6
Rollo, Antsygina & Tremblay, 2020 [7]	✓	✓	✓	✓	×*	✓	×	NA	5
Sampasa-Kanyinga et al. 2020 [41]	✓	✓	✓	✓	×*	✓	×	NA	5
Saunders et al. 2016 [3]	✓	✓	✓	✓	×*	✓	×	NA	5
Suc et at., 2024 [42]	✓	✓	✓	✓	✓	✓	×	NA	6
Tapia-Serrano et al. 2022 [1]	✓	✓	✓	✓	✓	✓	✓	✓	8
Wilhite et al. 2023 [17]	✓	✓	✓	✓	✓	✓	×	NA	6
Zhao et al. 2024 [35]	✓	✓	✓	✓	×	✓	×	✓	6

Note: ✓ = Yes; × = No; NA = not applicable. *: The analysis of the methodology quality of the study was carried out using a specific instrument for an evaluator and checked by others member (s) of the research team. Item 1 : Is the review based on a focused question that is adequately formulated and described?; Item 2 : Were eligibility criteria for included and excluded studies predefined and specified?; Item 3 : Did the literature search strategy use a comprehensive, systematic approach?; Item 4 : Were titles, abstracts, and full-text articles dually and independently reviewed for inclusion and exclusion to minimize bias?; Item 5 : Was the quality of each included study rated independently by two or more reviewers using a standard method to appraise its internal validity?; Item 6 : Were the included studies listed along with important characteristics and results of each study?; Item 7 : Was publication bias assessed?; Item 8 : Was heterogeneity assessed? (This question applies only to meta-analyses).

The studies included in the scoping review were analyzed regarding the methodology of the studies that met the inclusion criteria, this was carried out independently by two reviewers/authors (AFS and PCM). The non-agreement of these reviewers/authors regarding the evaluation of a study was resolved through a consensus meeting, and if the non-agreement persisted, it was decided by the third author (DASS). To assess the risk of bias/methodological quality of the studies, the *Quality Assessment of National Systematic Reviews and Meta- Analyses* (NHLBI) *Institutes of Health* (NIH) for reviews, with the aim of identifying the main points of weakness in the internal validity of review studies (QUALITY ASSESSMENT TOOL FOR OBSERVATIONAL COHORT AND CROSS-SECTIONAL STUDIES, 2021) .

This study quality assessment tool helps reviewers focus on concepts essential to the internal validity of review studies (research question, eligibility criteria, search strategy, peer review, study quality and characteristics, bias publication and heterogeneity, in cases of meta-analyses). The instrument consists of eight criteria to determine risk of bias/methodological quality, including whether the literature search strategy used a comprehensive approach. The following scores were assigned for each evaluation criterion: “yes” (✓), “no” (×) or “other” (when it was not possible to determine, not reported or not applicable). At the end of the evaluation of each study, a total score was assigned to it, based on the number of positive responses to the questionnaire in relation to the total number of questions (QUALITY ASSESSMENT TOOL FOR OBSERVATIONAL COHORT AND CROSS-SECTIONAL STUDIES, 2021).

Regarding the results of the methodological quality of the studies, six reviews presented the maximum total score (eight) [1,20,23,45,49,55]. Seven review studies obtained a score of five, which was the lowest score [3,7,11,15,22,25,41] (Supplementary Table 5).

Considering the eight criteria that analyzed the risk of bias/methodological quality, refered the question 7, whose described about publication bias, had less criteria attempt by the reviews. The total of review analyzed (28 reviews), 19 reviews did not assess the publication bias [3,7,11,15,17,18,21,22,24,25,35,36,39,41,42,46,48,50,53]. Besides, in two reviews, the methodological quality of each study included in the review was not assessed independently by two or more reviewers using a standard method to assess internal validity [11,35], and in four reviews the analysis of methodological quality was carried out using a specific instrument by one assessor and checked by another member(s) of the research team [3,7,22,41] (Question 5) (Supplementary Table 5).

It is noteworthy that Lettink et al. [15] carried out the search only in the Pubmed database (MEDLINE) and Maddren et al. [25] and Neville et al. [37] did not carry out manual searches of references, that is, they did not use a literature search strategy with a comprehensive and systematic approach as highlighted by the NIH instrument (Question 3). Only one review did not based on a focused question that was adequately formulated and described [56] (Question 1). Therefore, for the other items assessing the methodological quality of systematic reviews, all criteria were met (Supplementary Table 5).
